# The transcriptome of metamorphosing flatfish

**DOI:** 10.1186/s12864-016-2699-x

**Published:** 2016-05-27

**Authors:** Ricardo N. Alves, Ana S. Gomes, Kurt Stueber, Mbaye Tine, M. A. S. Thorne, H. Smáradóttir, Richard Reinhard, M. S. Clark, Ivar Rønnestad, Deborah M. Power

**Affiliations:** Comparative Endocrinology and Integrative Biology Group, Centro de Ciências do Mar - CCMAR, University of Algarve, Campus de Gambelas, 8005-139 Faro, Portugal; Department of Biology, University of Bergen, 5020 Bergen, Norway; Max Planck-Genome Centre, Max Planck-Institute for Plant Breeding Research, Carl-von-Linné-Weg 10, D-50829 Köln, Germany; British Antarctic Survey, Natural Environment Research Council, High Cross, Madingley Road, Cambridge, CB3 0ET UK; Samherji hf., Glerárgötu 30, 600 Akureyri, Iceland; Current address: Molecular Zoology Laboratory, Department of Zoology, University of Johannesburg, Auckland Park, 2006 South Africa

**Keywords:** Development, Flatfish, RNA sequencing, Thyroid hormone responsive, Tissue-remodelling, Transcriptome

## Abstract

**Background:**

Flatfish metamorphosis denotes the extraordinary transformation of a symmetric pelagic larva into an asymmetric benthic juvenile. Metamorphosis in vertebrates is driven by thyroid hormones (THs), but how they orchestrate the cellular, morphological and functional modifications associated with maturation to juvenile/adult states in flatfish is an enigma. Since THs act via thyroid receptors that are ligand activated transcription factors, we hypothesized that the maturation of tissues during metamorphosis should be preceded by significant modifications in the transcriptome. Targeting the unique metamorphosis of flatfish and taking advantage of the large size of Atlantic halibut (*Hippoglossus hippoglossus*) larvae, we determined the molecular basis of TH action using RNA sequencing.

**Results:**

*De novo* assembly of sequences for larval head, skin and gastrointestinal tract (GI-tract) yielded 90,676, 65,530 and 38,426 contigs, respectively. More than 57 % of the assembled sequences were successfully annotated using a multi-step Blast approach. A unique set of biological processes and candidate genes were identified specifically associated with changes in morphology and function of the head, skin and GI-tract. Transcriptome dynamics during metamorphosis were mapped with SOLiD sequencing of whole larvae and revealed greater than 8,000 differentially expressed (DE) genes significantly (*p* < 0.05) up- or down-regulated in comparison with the juvenile stage. Candidate transcripts quantified by SOLiD and qPCR analysis were significantly (r = 0.843; *p* < 0.05) correlated. The majority (98 %) of DE genes during metamorphosis were not TH-responsive. TH-responsive transcripts clustered into 6 groups based on their expression pattern during metamorphosis and the majority of the 145 DE TH-responsive genes were down-regulated.

**Conclusions:**

A transcriptome resource has been generated for metamorphosing Atlantic halibut and over 8,000 DE transcripts per stage were identified. Unique sets of biological processes and candidate genes were associated with changes in the head, skin and GI-tract during metamorphosis. A small proportion of DE transcripts were TH-responsive, suggesting that they trigger gene networks, signalling cascades and transcription factors, leading to the overt changes in tissue occurring during metamorphosis.

**Electronic supplementary material:**

The online version of this article (doi:10.1186/s12864-016-2699-x) contains supplementary material, which is available to authorized users.

## Background

Metamorphosis describes the “change in form” associated with the transition between life cycle stages in a wide range of animal taxa [1–8]. This transition can be accompanied by modifications in morphology, physiology, behavior, habitat and feeding mode. The endocrine system and in particular the thyroid hormones (THs), thyroxin (T4) and triiodothyronine (T3), play a central role in vertebrate metamorphosis acting as transcription factors (TFs) when they bind to their receptors. In amphibian metamorphosis, it is well established that THs directly or indirectly stimulate apoptosis and resorption of larval tissue and also promote growth, differentiation and remodelling of tissues that are crucial for the adult life form [8]. For instance, THs are involved in the control of muscle fibre apoptosis in the amphibian tail during its regression and also promote development of the limbs [9]. In amphibians the change in feeding habit from herbivore to carnivore during the transition from tadpole to frog is associated with TH driven remodelling of the intestine that changes from a long coiled tube into a complex differentiated organ [10–12]. Similarly, THs modulate the change in the amphibian integument from a simple to a stratified structure that is better adapted to terrestrial life [13].

Teleost fish also undergo a TH driven metamorphosis that marks the larval to juvenile transition [14, 15]. However, the term is more generally applied to the profound modifications associated with the change from bilateral symmetry to asymmetry during the larval-juvenile transition of flatfish (pleuronectiformes) [7, 16–20]. In flatfish metamorphosis the external morphology is dramatically transformed and they change from symmetric pelagic larvae to asymmetric benthic juveniles with both eyes on the upper, ocular side of the head (reviewed in Power et al. [7]). The external transformation in flatfish morphology is accompanied by a plethora of changes in the structure and function of tissues and organs. Chemical disruption of the thyroid axis using thiourea or methimazole (MMI) delays or stops stomach development in the Japanese flounder (*Paralichthys olivaceus*) [21] and otolith mineralization in the Southern flounder (*Paralichthys lethostigma*) [22]. The importance of THs during metamorphosis is further emphasized in other flatfish species where they have been shown to be important for the maturation of the muscle, stomach and skin [23–29].

Flatfish have a high economic value and include species such as the Atlantic halibut (*Hippoglossus hippoglossus*), common sole (*Solea solea*), Senegalese sole (*S. Senegalesis*), turbot (*Scophthalmus maximus*) and the half-smooth tongue sole (*Cynoglossus semilaevis*). Overfishing and high consumer demand for flatfish has made them an interesting target for aquaculture production and a better understanding of metamorphosis is of direct relevance for their efficient and successful production. Specific problems linked to failures during metamorphosis include feeding difficulties, reduced growth rate, arrested metamorphosis, abnormal pigmentation (albinism, ambicoloration or mosaicism), failed migration of the eye and skeletal deformities (reviewed in Power et al. [7]). Control of hatchery production requires an understanding of fish biology but also a comprehension of the mediators of metamorphosis, such as the THs and potentially other endocrine factors. Although there are numerous studies of flatfish metamorphosis, the endocrine and molecular basis of the tissue-specific modifications and the timing of the cascade of events that lead to metamorphosis are still largely unknown. Moreover, experiments that have blocked the thyroid axis during metamorphosis with drugs such as MMI do not significantly modify larval viability suggesting thyroid dependent [22, 30–32] and independent processes underpin this event. A complex task now lies ahead in establishing which developmental processes during metamorphosis are fully TH dependent and which genetic pathways and endocrine systems cross-talk with THs. Furthermore, it remains to be established how profoundly different processes such as skin maturation, eye migration and craniofacial remodelling or gastrointestinal tract (GI-tract) development can be regulated by the same endocrine factors.

One of the challenges of studying metamorphosis in fish larvae is their relatively small dimension, which means pools of larvae rather than individuals or tissues have generally been used which significantly reduces the resolution of such studies. The advantage of the biggest of flatfish, the Atlantic halibut, is the large size of the larvae and their slow metamorphosis (occurring over approx. 58 days), which means it is possible to analyze individuals or individual tissues. This is advantageous as pools of larvae contain a mixture of tissues and frequently contain individuals at different developmental stages making resolution of tissue specific changes in transcripts and proteins during metamorphosis difficult or impossible.

The working hypothesis of the present study is that since THs exert their action by binding to thyroid receptors that are ligand activated TFs the overt change in flatfish during metamorphosis will be preceded by significant modifications in the transcriptome of responsive tissues. For this reason, large scale analysis of tissue-specific transcriptional changes in responsive tissue should provide insight into the underlying molecular changes of tissue specific maturation. A 454 pyrosequencing approach was used to survey the tissue specific transcriptomes in the skin, GI-tract and head of metamorphosing Atlantic halibut and to also generate a reference transcriptome. SOLiD technology was then used to map the transcriptional changes in individuals (n = 3/stage) at different stages of metamorphosis. Differentially expressed (DE) transcripts during metamorphosis were identified by comparing the transcriptome at metamorphic stages (stage 7, 8 and 9 [33]) with juvenile (benthic) stages. Subsequently, genes of the thyroid axis, TH-responsive transcripts and candidate genes that underpin remodelling and maturation of tissues during metamorphosis were identified and analyzed by quantitative PCR (qPCR).

## Results

### 454 transcriptome sequencing

#### Transcriptome annotation

In spite of stringent quality control of the RNA used for 454 library construction, the number of reads resulting from the stage-specific libraries for skin, GI-tract and head of Atlantic halibut post-embryonic larvae at different metamorphic stages was highly variable. A total of 134 Mbp were produced for the tissue assemblies using MIRA V3 (http://sourceforge.net/projects/mira-assembler/files/) and they assembled into 65,530, 38,426 and 90,676, contigs for skin, GI-tract, and head, respectively. The contigs from the skin, GI-tract and head tissue assemblies were submitted to an iterative stringent four-step local Blast approach (Additional file 1). The tissue assemblies were successfully annotated and for the GI-tract library 60 % of the initial contigs had a good Blast match after the first 3 annotation steps and 57 % of the contigs were annotated for the skin and head libraries (Table 1). Most of the contigs were successfully annotated in the first step of Blastx against the zebrafish refseq protein db.

**Table 1:** Summary of the Blast results used for annotation of the head, skin and GI-tract assembly

	Skin	GI-tract	Head
Total number of contigs	65,530	38,426	90,676
Blastx refseq protein zebrafish	27,312	12,845	42,040
Blastx vertebrate swissprot db	3,560	1,141	5,209
Blastx protein bony fish db	6,791	9,166	4,559
Percentage of contigs with annotation	57 %	60 %	57 %
Additional blast matches to non-annotated ESTs (%)	11,418 (17 %)	12,354 (32 %)	14,460 (13 %)
Percentage of contigs with no database match	26 %	8 %	30 %

#### Gene Ontology and KEGG analysis

**Fig. 1:**
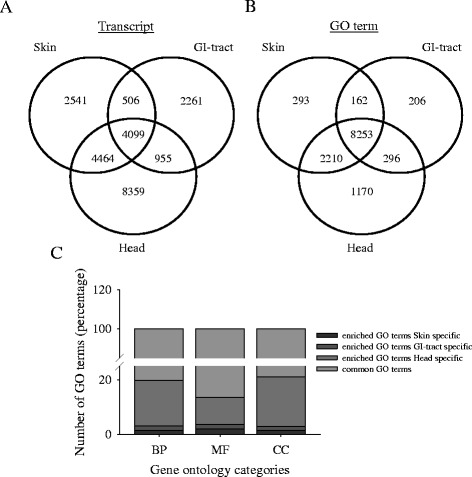
Atlantic halibut skin, gastrointestinal tract and head transcriptome annotation. **a** Venn diagram of common and unique tissue transcripts (using the transcript name); **b** Venn diagram representing the common and specific tissue gene ontology (GO) terms (using the unique GO terms); **c** Diagram representing the relative abundance of shared and tissue specific enriched GO terms by GO category (using the over/under-represented GO terms from the Fisher’s exact test)

The active transcriptome in each tissue analyzed was assumed to be equivalent to the number of contigs identified (Fig. 1a). Comparative analyses between the transcriptomes revealed that 2,541, 2,261 and 8,359 transcripts were unique to the skin, GI-tract and head assembly, respectively. In addition, 4,099 transcripts were common between the three tissues. The head and skin, head and GI-tract and GI-tract and skin shared a further 4,464, 955 and 506 transcripts, respectively (Fig. 1a).

The complete functional (GO) annotation for the skin, GI-tract and head transcriptomes (Additional files 2, 3 and 4, respectively) assigned a total of 12,577 different GO terms to the three tissue assemblies. Comparisons of the assigned GO terms for the skin, GI-tract and head transcriptome assemblies revealed 8,253 common GO terms and 293 identified only in the skin, 206 only in the GI-tract and 1,170 only in the head (Fig. 1b). The most abundant GO terms (level 2) for biological process (BP), molecular function (MF) and cellular component (CC) showed no major differences between skin, GI-tract and head (Additional file 5). Cellular process (17 %), metabolic process (16 %) and biological regulation (10 %) were the most representative GO terms in the category biological processes. Other key biological processes linked to development (7 %), localization (6 %), signaling (5 %), cell proliferation (3 %) and death (3 %) were also found (Additional file 5).

For molecular function, the most abundant GO terms (level 2) in the skin and head transcriptomes were binding, catalytic activity and structural molecule activity (Additional file 5). For the GI-tract transcriptome, binding and nucleic acid binding transcription factor activity were the most frequent MF GO terms. The exception was the GI-tract for which GO terms specific for DNA binding transcription factor activity (~20 %) were more highly represented, when compared to the other tissues (Additional file 5). Within the cellular component category, the most represented GO terms in the three tissues transcriptomes were cell, organelle, macromolecule complex and membrane (Additional file 5).

Fisher’s exact tests were applied to detect significantly over/under-represented GO terms resulting from analysis of the tissue-specific transcriptomes (FDR < 0.05). Comparison of the overall GO enrichment for the tissue specific transcriptome revealed that the highest enrichment was associated with the BP category (approx. 60–73 %), followed by the MF category (16–31 %). Of the 2,316 enriched GO terms identified for the tissue transcriptomes, 8.2 % (190), 9.2 % (214) and 82.6 % (1,912) were from skin, GI-tract, and head, respectively. Figure 1c shows the representative enriched GO terms for each tissue assembly for BP, MF, and CC gene ontology categories. The halibut head transcriptome had the highest enrichment of BP GO terms (1,395) and they corresponded to 16.7 % of the overall GO terms for this tissue, followed by the GI-tract (148 GO terms) and the skin (114 GO terms) (Fig. 1c). Similarly, MF GO terms were most enriched in the head (303 GO terms and 9.9 % of overall enriched terms) relative to the skin (58 GO terms) and the GI-tract (51 GO terms).

In the skin transcriptome, GO terms related to the muscle system, development and morphogenetic processes, including epidermis development, appendage morphogenesis, and transcripts involved in cellular response to hormone stimulus were overrepresented along with immune development and pigmentation (Additional file 6). Significant GO categories in the GI-tract that were overrepresented included digestion, proteolysis and lipid metabolism such as the cholesterol metabolic process and triglyceride mobilization (Additional file 7). In the head transcriptome, significantly overrepresented GO terms included development of the nervous system, spinal cord and otoliths, cartilage and endochondral bone. In addition, head-specific GO terms, such as pituitary gland development and thyroid hormone metabolic processes were also significantly overrepresented (Additional file 8).

REVIGO clustering of enriched Biological Process GO terms for the skin transcriptome identified phosphocreatine metabolism and response to lipopolysaccharides and biotic stimulus. In REVIGO clustering of enriched BP GO terms in the GI-tract transcriptome identified digestion, cell proliferation, rRNA transcription, lipid storage and immune system and response (e.g. foam cell differentiation, positive regulation of macrophage derived foam cell differentiation, low density lipoprotein particle remodeling, lipoproteins transport). In the head, transcriptome REVIGO clustering of enriched BP GO categories identified nervous system development (e.g. glutamate receptor and neuropeptide signaling pathways, proliferation and apoptosis of neural precursor cells, regulation of synaptic plasticity and synaptic vesicle transport), blood vessel morphogenesis, and immune and defense response (T cell activation).

More than 125 metabolic pathways were identified via KEGG mapping comprising ~1,250 different enzyme codes and more than 9,000 of the Atlantic halibut contigs matched an enzyme code (EC) (Additional file 9). Overall, there was considerable similarity in the metabolic pathway enrichment between the three tissues but this was unsurprising since many of the pathways were linked to cellular metabolism. Of note was the lower representation of sphingolipids, inositol lipid and phospholipid pathways in the GI-tract. The GI-tract, a soft tissue, had a notable reduction compared to the skin and head of metabolic pathways involved in chondrogenic matrix generation but had an increase in starch and sucrose metabolic pathways relative to the head (Additional file 9).

#### Tissue development/morphogenesis – identification of putative tissue-specific genes

Blast of the skin transcriptome against the *in-house* skin-specific database that contained genes characteristic of skin in other vertebrates identified 33 transcripts for skin development and morphogenesis and 40 for pigmentation (Table 2). Abundant transcripts included the collagens (col1a1, col1a2), genes involved in pigmentation, melanocyte differentiation and melanosome transport (e.g. apoptosis regulator bax, dedicator of cytokinesis 7, dopachrome tautomerase, lysosomal-trafficking regulator), (Table 2). Several signal transduction pathways were identified in the skin transcriptome including Notch (>70 transcripts), Wnt (>100 transcripts) and Sonic Hedgehog (Shh) (>30 transcripts) (Additional file 2).

**Table 2:** Genes in vertebrate skin development and pigmentation identified in the Atlantic halibut skin transcriptome

Contig ID	Protein name	Acronym	Accession no.	Species	E-value	Biological role
lcst_c59341	Transcription factor ap-2 alpha (activating enhancer binding protein 2 alpha)	tfap2c	A2APA8	*Mus musculus*	3E-38	*Skin development*
lcst_c6882	Epithelial discoidin domain-containing receptor 1	ddr1	B0V2H8	*Mus musculus*	2E-71	
lcst_c52199	Adenomatosis polyposis coli, isoform CRA_a	apc	B2RUG9	*Mus musculus*	8E-28	
lcst_c5213	T-cell factor-4 variant L	tcf7l2	E2GH26	*Homo sapiens*	1E-39	
lcst_c348	Desmoplakin	dsp	E9Q557	*Mus musculus*	0	
lcst_c611	Collagen, type V. Alpha 2	col5a2	F1LQ00	*Rattus norvegicus*	1E-124	
lcst_rep_c10540	Keratin, type I cytoskeletal 9	krt9	F1M7K4	*Rattus norvegicus*	2E-59	
lcst_c88	Sratifin (14-3-3 protein sigma)	sfn	O70456	*Mus musculus*	4E-74	
lcst_c38806	Steryl-sulfatase	sts	P15589	*Rattus norvegicus*	2E-61	
lcst_c1342	Gap junction beta-3 protein	gjb3	P28231	*Mus musculus*	2E-56	
lcst_c5669	Macrophage migration inhibitory factor	mif	P30904	*Rattus norvegicus*	3E-30	
lcst_c7497	Cellular tumor antigen p53	Trp63	Q569E5	*Mus musculus*	8E-48	
lcst_c26088	Transcription factor 15	tcf15	Q60756	*Mus musculus*	4E-31	
lcst_c32091	Copper-transporting atpase 1	atp7a	Q64430	*Mus musculus*	8E-47	
lcst_c62343	Calcium release channel (Ryanodine receptor 1)	crc1, ryr1	Q6LAA3	*Sus scrofa*	3E-26	
lcst_c54854	Lethal(2) giant larvae protein homolog 2	llgl2	Q7SZE3	*Danio rerio*	8E-58	
lcst_c28836	Extracellular matrix protein FRAS1	fras1	Q80T14	*Mus musculus*	1E-70	
lcst_c6455	Alanine-trna ligase, cytoplasmic	aars	Q8BGQ7	*Mus musculus*	0	
lcst_c50366	Nerve growth factor receptor (TNFR superfamily, member 16)	ngfr	Q8CFT3	*Mus musculus*	7E-34	
lcst_c1016	Delta(24)-sterol reductase	dhcr24	Q8VCH6	*Mus musculus*	0	
lcst_c60747	Long-chain fatty acid transport protein 4	slc27a4	Q91VE0	*Mus musculus*	2E-121	
lcst_c28754	Platelet-derived growth factor subunit A	pdgfa	Q99L56	*Mus musculus*	1E-29	
lcst_c4164	Collagen, type I, alpha 2	Col1a2	Q91VL4	*Mus musculus*	9E-91	
lcst_c27614	B-cell lymphoma/leukemia 11B	bcl11b	Q99PV8	*Mus musculus*	2E-58	
lcst_rep_c10461	Junction plakoglobin	jup	Q9PVF7	*Danio rerio*	4E-180	
lcst_c57261	Suppressor of fused homolog	sufu	Q9Z0P7	*Mus musculus*	3E-64	
lcst_c27807	Serum response factor	srf	Q9JM73	*Mus musculus*	9E-58	*Skin morphogenesis*
lcst_c6845	Collagen, type I, alpha 1	col1a1	Q99LL6	*Mus musculus*	1E-117	
lcst_c4164	Collagen, type I, alpha 2	col1a2	Q91VL4	*Mus musculus*	9E-91	
lcst_c25461	Fibroblast growth factor receptor 1-A	fgfr1a	Q90Z00	*Danio rerio*	6E-112	
lcst_c7432	Transformation related protein 63	trp63	Q5CZX0	*Mus musculus*	2E-131	
lcst_c34953	v-erb-b2 erythroblastic leukemia viral oncogene homolog 3b	erbb3b	F1Q4T5	*Danio rerio*	2E-83	*Developmental pigmentation*
lcst_c23922	ATPase, H+ transporting, V0 subunit B	atp6v0b	F1QYM3	*Danio rerio*	1E-82	
lcst_c4704	Melanocyte protein pmel-like	pmela	Q4VW61	*Danio rerio*	1E-56	
lcst_c40663	Macrophage colony-stimulating factor 1 receptor	csf1r	Q9I8N6	*Danio rerio*	1E-40	
lcst_c24693	Vacuolar protein sorting-associated protein 18 homolog	vps18	P59015	*Danio rerio*	5E-139	*Endosome to pigment granule transport*
lcst_c52806	Mediator of RNA polymerase II transcription subunit 12	med12	Q2QCI8	*Danio rerio*	3E-122	*Iridophore differentiation*
lcst_c7916	Forkhead box D3	foxd3	Q502Q4	*Danio rerio*	2E-107	
lcst_c467	Rab escort protein 1	chm	Q6RFG0	*Danio rerio*	1E-114	
lcst_c8298	Mindbomb E3 ubiquitin protein ligase 2	mib2	A0AR23	*Danio rerio*	1E-138	*Melanosome differentiation*
lcst_c2918	Transcription factor Sox-10	sox10	A4IIJ8	*Xenopus tropicalis*	1E-49	
lcst_rep_c15027	Mindbomb E3 ubiquitin protein ligase 1	mib1	B3DGQ0	*Danio rerio*	9E-66	
lcst_c31755	Transient receptor potential cation channel, subfamily M, member 7	trpm7	B3DK48	*Danio rerio*	5E-82	
lcst_c8173	Glutamine-fructose-6-phosphate transaminase 1	gfpt1	Q3S344	*Danio rerio*	2E-117	
lcst_c8009	vacuolar protein sorting-associated protein 11 homolog	vps11	Q4G0A0	*Danio rerio*	1E-135	
lcst_c31151	RNA polymerase-associated protein LEO1	leo1	Q6NYV9	*Danio rerio*	1E-50	
lcst_c366	Histone deacetylase	hdac1	Q8JIY7	*Danio rerio*	0	
lcst_c1665	Transcription factor AP2 alpha 2	tfap2a	Q8UVE5	*Danio rerio*	3E-55	
lcst_c24989	Microphthalmia-associated transcription factor a	mitfa	Q9PWC2	*Danio rerio*	5E-24	
lcst_rep_c15543	Presenilin-1	psen1	Q9W6T7	*Danio rerio*	2E-62	
lcst_c2922	Adaptor-related protein complex AP-1, mu subunit 1	ap1m1	Q3UG16	*Mus musculus*	1E-81	*Melanosome organization*
lcst_c31194	Tyrosinase related protein	tyrp1	Q6DGE4	*Danio rerio*	1E-84	
lcst_c60613	ras-related protein rab-8a	rab8a	A4FVK4	*Danio rerio*	8E-99	*Melanosome transport*
lcst_c1994	Inositol-pentakisphosphate 2-kinase	ippk	Q4JL91	*Danio rerio*	2E-33	
lcst_c2227	RAB11a, member RAS oncogene family	rab11a	Q5U3E1	*Danio rerio*	7E-75	
lcst_c43921	Tetratricopeptide repeat protein 8	ttc8	Q6P5I7	*Danio rerio*	8E-66	
lcst_rep_c10145	Chaperonin containing TCP1, subunit 2 (Beta)	cct2	Q6PBW6	*Danio rerio*	0	
lcst_c6090	Synembryn-B	ric8b	Q6DRJ9	*Danio rerio*	3E-77	*Pigment cell development*
lcst_c1817	Cadherin-2	cdh2	Q90275	*Danio rerio*	0	
lcst_c2222	N-ethylmaleimide-sensitive factor	Nsfa	Q7ZU50	*Danio rerio*	0	*Pigment granule localization*
lcst_c31478	Dedicator of cytokinesis protein 7	dock7	A2A9M4	*Mus musculus*	9E-84	*Pigmentation*
lcst_c30539	Dopachrome tautomerase	dct	A3KDL7	*Sus scrofa*	1E-36	
lcst_c2174	RNA polymerase-associated protein Ctr9 homolog	ctr9	A3KDM3	*Danio rerio*	0	
lcst_c307	Phosphoribosylglycinamide formyltransferase	gart	Q9I9E6	*Danio rerio*	0	
lcst_c45538	Lysosomal-trafficking regulator	lyst	Q9Z2X9	*Rattus norvegicus*	6E-86	
lcst_c2408	Apoptosis regulator BAX	bax	Q07813	*Mus musculus*	6E-34	*Positive regulation of developmental pigmentation*
lcst_c8805	A disintegrin and metalloproteinase with thrombospondin motifs 9	adamts9	E9PYV8	*Mus musculus*	4E-67	*Positive regulation of melanocyte differentiation*
lcst_c546	Ras-related protein ralb-A	ralb-a	Q9YH09	*Xenopus laevis*	3E-65	*Regulation of developmental pigmentation*
lcst_c6473	Guanine nucleotide binding protein, alpha 11	gna11	Q3UPA1	*Mus musculus*	1E-131	*Regulation of melanocyte differentiation*
lcst_c4062	Beta-adrenergic receptor kinase 2	adrbk2	P26819	*Rattus norvegicus*	6E-144	*Rhodopsin metabolic process*

Transcripts identified in the Atlantic halibut skin transcriptome that are involved in vertebrate skin development/morphogenesis and pigmentation. The contig ID of the skin transcriptome assembly, Protein name, acronym, accession number (no.), organism and e-value are shown for each protein and they are grouped by biological function (when identified)

When the GI-tract transcriptome was compared with the *in-house* GI-tract specific database, 72 genes were identified. Identified gene transcripts included those with sequence similarity to signal transduction pathways, such as Sonic Hedgehog (Shh), Wnt and bone morphogenic protein (Bmp), as well as genes involved in gastric function (Table 3, Additional file 3).

**Table 3:** Genes of the vertebrate digestive system identified in the Atlantic halibut GI-tract transcriptome

Contig ID	Protein name	Acronym	Accession no.	Organism	E-value	Biological role
lcgut_c14148	Adenosine deaminase	Ada	Q4FK28	*Mus musculus*	1E-41	*Embryonic digestive tract development*
lcgut_c24687	Protein kinase domain-containing protein, cytoplasmic	Pkdcc	Q5RJI4	*Mus musculus*	2E-46	
lcgut_c25681	Transforming growth factor beta receptor type 3 isoform b precursor	Tgfbr3	Q90998	*Gallus gallus*	4E-36	
lcgut_c26069	Sal-like protein 1	Sall1	Q6P5E3	*Mus musculus*	1E-67	
lcgut_c31048	Forkhead box protein F1	Foxf1	Q28BS5	*Xenopus tropicalis*	1E-55	
lcgut_c34421	Proprotein convertase subtilisin/kexin type 5	Pcsk5	Q04592	*Mus musculus*	1E-152	
lcgut_c17559	Ribosomal protein S6 kinase	Rps6ka3a	Q7ZVH8	*Danio rerio*	5E-75	*Digestive tract development*
lcgut_c33030	TGF-beta receptor type-2	Tgfbr2	P38438	*Rattus norvegicus*	9E-40	
lcgut_c35109	G2/mitotic-specific cyclin-B1	Ccnb1	P24860	*Mus musculus*	7E-43	
lcgut_c901	Cytochrome P450 family 1 subfamily a polypeptide 1	Cyp1a1	Q05A20	*Mus musculus*	3E-156	
lcgut_c17435	Retinoid X nuclear receptor alpha	Nr2b1	F1D8Q5	*Homo sapiens*	1E-76	*Midgut development*
lcgut_c186	Retinal dehydrogenase 1	Aldh1a1	P24549	*Mus musculus*	2E-179	
lcgut_c24118	Ornithine transcarbamylase, isoform CRA_a	Otc	Q543H3	*Mus musculus*	3E-72	
lcgut_c2518	Arginase-2, mitochondrial	Arg2	O08691	*Mus musculus*	2E-56	
lcgut_c2565	Proto-oncogene tyrosine-protein kinase receptor Ret	Ret	P07949	*Homo sapiens*	3E-35	
lcgut_rep_c3756	Hydroxymethylglutaryl-coa synthase, mitochondrial	Hmgcs2	P54869	*Mus musculus*	5E-171	
lcgut_c25668	Homeobox protein Nkx-3.2	Nkx3-2	P97503	*Mus musculus*	6E-35	*Intestinal epithelial cell development*
lcgut_c374	GATA-binding protein 6	Gata6	ENSDARP00000051997	*Danio rerio*	0	
lcgut_c981	Protein-tyrosine kinase 6	Ptk6	Q64434	*Mus musculus*	9E-64	
lcgut_rep_c3687	Anterior gradient protein 2 homolog	Agr2	Q5RZ65	*Danio rerio*	7E-66	
lcgut_rep_c15924	Polypyrimidine tract binding protein 1a	Ptbp1a	Q503D3	*Danio rerio*	7E-112	*Intestinal epithelial structure maintenance*
lcgut_c2805	Heart and neural crest derivatives expressed transcript 2	Hand2	Q5XJD8	*Danio rerio*	7E-49	*Determination of intestine left/right asymmetry*
lcgut_c17287	Platelet-derived growth factor receptor alpha	Pdgfra	P26618	*Mus musculus*	4E-86	*Embryonic digestive tract morphogenesis*
lcgut_c2545	Sonic hedgehog protein A	Shha	Q92008	*Danio rerio*	2E-41	
lcgut_c30553	DNA-binding protein inhibitor ID-2	Id2	Q6PBD7	*Xenopus tropicalis*	8E-44	
lcgut_c32097	GATA binding protein 4	Gata4	ENSDARP00000090333	*Danio rerio*	2E-42	
lcgut_c37022	Transcription factor 21	Tcf21	Q32PV5	*Danio rerio*	4E-69	
lcgut_c17569	Hepatocyte nuclear factor 1-beta-A	Hnf1ba	A1L1N5	*Danio rerio*	5E-66	*Digestive tract morphogenesis*
lcgut_c19328	Caudal type homeobox 1	Cdx1	ENSP00000367043	*Homo sapiens*	3E-31	
lcgut_c20926	Mib protein	Mib	B3DGQ0	*Danio rerio*	8E-45	
lcgut_c22447	Ephrin type-B receptor 3	Ephb3	P54754	*Mus musculus*	1E-39	
lcgut_c22825	Probable rna-binding protein 19	Rbm19	Q6DRI6	*Danio rerio*	3E-32	
lcgut_c25556	Vang-like 2 (Van gogh, Drosophila), isoform CRA_b	Vangl2	D3YY75	*Mus musculus*	4E-38	
lcgut_c26	Claudin 15 like	Cldn15a	Q7T2E7	*Danio rerio*	3E-82	
lcgut_c27711	Secreted frizzled-related protein 1	Sfrp1	Q8C4U3	*Mus musculus*	1E-49	
lcgut_c38245	Protein kinase C iota type	Prkci	Q90XF2	*Danio rerio*	2E-87	
lcgut_c14247	Beta-1,3-galactosyl-O-glycosyl-glycoprotein beta-1,6-N-acetylglucosaminyltransferase 3	Gcnt3	Q5JCT0	*Mus musculus*	2E-57	*Intestinal absorption*
lcgut_c14318	2-acylglycerol O-acyltransferase 2	Mogat2	Q80W94	*Mus musculus*	8E-73	
lcgut_c425	Sodium/glucose cotransporter 1	Slc5a1	F6XY79	*Mus musculus*	6E-142	
lcgut_rep_c3628	Fatty acid binding protein 2, intestinal	Fabp2	Q53YP5	*Mus musculus*	9E-47	
lcgut_rep_c38188	Fatty acid binding protein 1, liver	Fabp1	Q3V2F7	*Mus musculus*	9E-35	
lcgut_c2583	ATP-binding cassette sub-family G member 5	Abcg5	Q99PE8	*Mus musculus*	6E-168	*Intestinal cholesterol absorption*
lcgut_c280	Niemann-Pick C1-like protein 1	Npc1l1	Q6T3U4	*Mus musculus*	6E-140	
lcgut_c30941	Caveolin	Cav1	Q6YLH9	*Danio rerio*	3E-65	
lcgut_c3243	Pancreatic triacylglycerol lipase	Pnlip	Q6P8U6	*Mus musculus*	6E-99	
lcgut_c34406	Sterol O-acyltransferase 2	Soat2	O75908	*Homo sapiens*	1E-89	
lcgut_c508	ATP-binding cassette sub-family G member 8	Abcg8	Q9DBM0	*Mus musculus*	0	
lcgut_rep_c3552	Pancreatic lipase	pl	D4P6H2	*Sus scrofa*	4E-83	
lcgut_rep_c3840	Annexin	Anxa2b	Q6DHD8	*Danio rerio*	2E-133	
lcgut_c20773	Cholecystokinin receptor type A	Cckar	O08786	*Mus musculus*	1E-47	*Gastric acid secretion/Regulation*
lcgut_c14119	Pepsinogen A form iib precursor	Pep2b	AAD56284	*Pseudopleuronectes americanus*	0	
lcgut_c1099	Bone morphogenetic protein 2a	Bmp2a	ENSDARP00000013686	*Danio rerio*	3E-35	
lcgut_c17255	Histamine N-methyltransferase	Hnmt	ENSORLP00000025386	*Oryzias latipes*	1E-87	
lcgut_c17921	MAD homolog 9	Smad9	ENSDARP00000031108	*Danio rerio*	1E-85	
lcgut_c20823	Histamine receptor H2	Hrh2	ENSORLP00000004946	*Oryzias latipes*	2E-24	
lcgut_c22281	Epidermal growth factor receptor a	Egfra	ENSDARP00000125265	*Danio rerio*	4E-42	
lcgut_c25854	SRY-box containing gene 2	Sox2	ENSDARP00000095266	*Danio rerio*	2E-21	
lcgut_c28926	Forkhead box A1 (HNF3?)	Foxa1	ENSDARP00000002213	*Danio rerio*	7E-44	
lcgut_rep_c6868	Protein wntmber 5a	Wnt5a	F1Q8M2	*Danio rerio*	5E-26	

List of candidate genes in the Atlantic halibut GI-tract transcriptome assembly that are involved in vertebrate digestive system development and morphogenesis. The contig ID of the GI-tract transcriptome assembly, protein name, acronym, accession number (no.), organism and e-value are shown for each protein and they are grouped by biological function (when identified)

The head transcriptome contained genes involved in thyroid gland development and thyroid hormone physiology, such as enzymes involved in the activation or inactivation of THs (DIO1, DIO2, DIO3), TH receptors (TRαB and TR*b*) and other nuclear receptors (Table 4). The pigmentation genes identified in head were coincident with those found in skin. Signalling pathways associated with development were well represented and included in the head transcriptome: i) the Wnt receptor signalling pathway (including casein kinases, low-density lipoprotein receptor related proteins, frizzled-related proteins, spontins, wnt11, wnt7, wnt8, wnt9); ii) the transforming growth factor beta (TGFβ) receptor signalling pathway (including activins, bone morphogenetic proteins, collagens, latent-transforming growth factor beta-binding proteins and tgf-beta receptors); iii) the Notch signalling pathway; iv) the Hippo signalling cascade, and v) the Hedgehog signalling pathway (Additional file 4).

**Table 4:** Genes involved in the TH axis identified in the Atlantic halibut head, skin and GI-tract transcriptomes

Protein name	Acronym	Accession no.	Organism	Head	Skin	GI-tract	Biological role
Thyrotropin-releasing hormone	TRH	ACI68323	*Salmo salar*	lchead_c49703			*Hormone-mediated signalling pathway*
Bteb1 protein	Klf9	Q8CEC4	*Mus musculus*	lchead_c5876	lcst_c61990		*Cellular response to thyroid hormone stimulus*
Cathepsin B	Ctsb	P10605	*Mus musculus*	lchead_rep_c17340		lcgut_c971	
Cathepsin S	Ctss	Q3UD32	*Mus musculus*	lchead_c2408	lcst_c25054		
GAS2-like protein 1	Gas2l1	Q8JZP9	*Mus musculus*	lchead_c63583			
Mediator of RNA polymerase II transcription subunit 1	Med1	Q925J9	*Mus musculus*	lchead_c54375	lcst_c53801		
Rhombotin-2	Lmo2	A2BHP2	*Mus musculus*	lchead_rep_c19211			
Tyrosine-protein kinase receptor	Kit	Q63116	*Rattus norvegicus*	lchead_c88982		lcgut_c981	
GATA binding protein 3	GATA 3	Q0ZHH4	*Sus scrofa*	lchead_c10759			*Positive regulation of thyroid hormone generation*
Serine protease hepsin	Hpn	O35453	*Mus musculus*	lchead_c1056	lcst_c55583		
Fibroblast growth factor 10	Fgf10	O35565	*Mus musculus*	lchead_c59444			*Thyroid gland development*
Forkhead box E3	foxe3	B0UXI3	*Danio rerio*	lchead_c14669	lcst_c7916		
Heart and neural crest derivatives expressed transcript 2	hand2	Q5XJD8	*Danio rerio*	lchead_c14402			
Hematopoietically-expressed homeobox protein hhex	hhex	Q9IAV3	*Danio rerio*	lchead_c76036			
Homeobox protein Nkx2.1a	nkx2.1a	Q9I8L7	*Danio rerio*	lchead_rep_c28603			
MAD homolog 3	Smad3	A2CG44	*Mus musculus*	lchead_c8437	lcst_c2973	lcgut_c21904	
NK2 homeobox 1	Nkx2-1	Q6PFE0	*Mus musculus*	lchead_rep_c45357			
Sonic hedgehog protein	Shh	Q62226	*Mus musculus*	lchead_c10675		lcgut_c2545	
T-cell acute lymphocytic leukemia protein 1 homolog	tal1	O93507	*Danio rerio*	lchead_c1186			
Thyroglobulin	Tg	O08710	*Mus musculus*	lchead_c4966			
Transcription factor gata5	gata5	Q9W6U0	*Danio rerio*	lchead_c49264			
Transforming growth factor beta-2	Tgfb2	P27090	*Mus musculus*	lchead_rep_c18013	lcst_c97		
Vascular endothelial growth factor A-A	vegfaa	O73682	*Danio rerio*	lchead_rep_c38578			
Vascular endothelial growth factor receptor kdr-like	kdrl	Q8AXB3	*Danio rerio*	lchead_c15716			
Aldehyde dehydrogenase family 1 member A3	Aldh1a3	G3UWP3	*Mus musculus*	lchead_rep_c51532			*Thyroid hormone binding*
Cathepsin H	CTSH	B2D1T2	*Sus scrofa*	lchead_rep_c18375	lcst_c35788		
Retinaldehyde dehydrogenase 3	ALDH6	Q9DD46	*Gallus gallus*	lchead_c4855	lcst_c276		
Estrogen receptor alpha	-	Q2PUG8	*Hippoglossus hippoglossus*	lchead_c11863			*Thyroid hormone receptor activity*
Farnesoid X activated receptor	-	Q8SPF5	*Oryctolagus cuniculus*	lchead_c38827			
Nuclear receptor subfamily 1, group D, member 4	nr1d4a	B8A510	*Danio rerio*	lchead_c5000	lcst_c4716		
Nuclear receptor subfamily 1, group D, member 1	nr1d1	Q503Y6	*Danio rerio*	lchead_c56865		lcgut_c766	
Nuclear receptor subfamily 2, group E, member 3	nr2e3	A0FCT3	*Xenopus tropicalis*	lchead_rep_c19272	lcst_c50411		
Nuclear receptor subfamily 1, group H, member 4	nr1h4	Q6DGW7	*Danio rerio*	lchead_c38092			
Orphan nuclear receptor BXR-beta	nr1i2	Q9DF24	*Xenopus laevis*	lchead_c53830			
Orphan nuclear receptor HZF-2	Nr1d2	Q62702	*Rattus norvegicus*	lchead_c13737			
Rev-erbgamma-B	nr1d4b	Q1L683	*Danio rerio*	lchead_c801			
Thyroid hormone receptor alpha B	TRαB	B7XBZ0	*Solea senegalensis*	lchead_c73126	lcst_c51070		
Thyroid hormone receptor beta	TRβ	A8R655	*Solea senegalensis*	lchead_c15487	lcst_c29077		
Monocarboxylate transporter 10	MCT10	NP_001073497	*Danio rerio*	lchead_c4262	lcst_c309		*Thyroid hormone transmembrane transporter activity*
Solute carrier organic anion transporter family member 1A5	Slco1a5	E0CX25	*Mus musculus*	lchead_c60364			
Solute carrier organic anion transporter family member 4A1	Slco4a1	Q8K078	*Mus musculus*	lchead_c13235			
Thyroxine-binding globulin	Serpina7	P35577	*Rattus norvegicus*	lchead_c2058			
Monocarboxylate transporter 8	MCT8	NP_001245159	*Danio rerio*	lchead_c13478	lcst_c54175	lcgut_c2689	
Canalicular multispecific organic anion transporter 1	Abcc2	Q63120	*Rattus norvegicus*	lchead_c7363		lcgut_c1215	
Paired box protein Pax-8	PAX8	Q06710	*Homo sapiens*	lchead_c53444			*Thyroid-stimulating hormone receptor activity*
Thyroid stimulating hormone receptor	tshr	F1Q981	*Danio rerio*	lchead_c79782			
Iodothyronine deiodinase type I	DIO1	B1B569	*Takifugu rubripes*	lchead_c4984	lcst_c3609	lcgut_c27346	*Thyroxine 5-deiodinase activity*
Iodothyronine deiodinase type II	DIO2	B3Y056	*Oryzias latipes*	lchead_c13614			
Iodothyronine deiodinase type III	DIO3	B1B572	*Takifugu rubripes*	lchead_c39730	lcst_c58807		

List of candidate genes identified in the Atlantic halibut head transcriptome with a role in thyroid gland development and thyroid hormone (TH) synthesis, transport and activity. Protein name, symbol, accession number (no.) and organism are shown for each gene product and they are grouped by biological function (when associated). Contig ID from the head, GI-tract and skin transcriptome assembly is given

#### Identification of thyroid hormone responsive genes

**Fig. 2:**
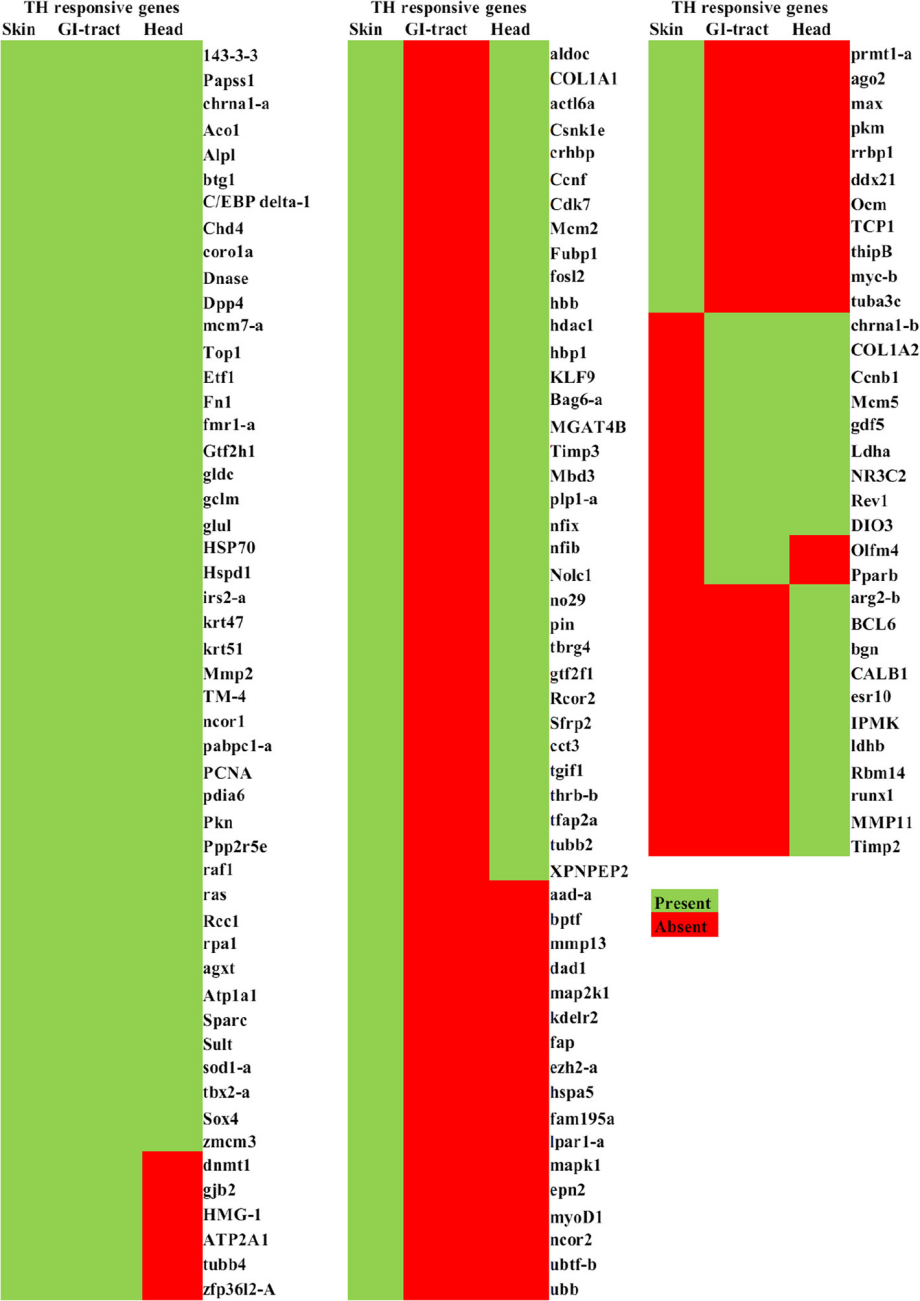
Putative thyroid hormone (TH) responsive genes identified in the Atlantic halibut transcriptomes. Heat map of putative thyroid hormone (TH) responsive genes identified in the transcriptomes of skin, GI-tract and head of metamorphosing Atlantic halibut. The acronyms of TH-responsive genes are indicated and full names are given in Additional file 18

Overall, 135 putative TH-responsive genes were identified in the skin, GI-tract and head transcriptome, which included TFs, genes involved in DNA replication, cell proliferation, cell growth and differentiation and collagen synthesis and degradation (Fig. 2). The skin transcriptome contained 113 putative TH-responsive genes that mainly corresponded to structural proteins, proteases, actins, transmembrane proteins, and several membrane transport proteins of the solute carrier group (Fig. 2). The GI-tract transcriptome contained 62 putative TH-responsive genes and included genes involved in DNA replication, cell cycle and metabolic pathways (Fig. 2). The head transcriptome was enriched with 99 putative TH-responsive genes of which 11 were specific to the head transcriptome and included TFs, DNA replication and ion binding proteins (Fig. 2).

### SOLiD transcriptome comparison between metamorphic stage transitions

#### Identification of differentially expressed transcripts during metamorphosis

**Fig. 3:**
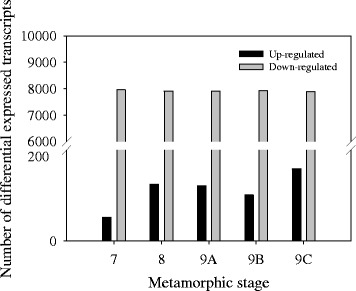
Differentially expressed transcripts between Atlantic halibut metamorphic stages and juveniles. Graphical representation of the relative number of DE transcripts (up- and down-regulated) identified when pro-metamorphic (stage 7), proclimax-metamorphic (stage 8) and metamorphic climax (9A, 9B and 9C) Atlantic halibut are compared with the juvenile post-metamorphic stage

Pairwise comparisons of whole larvae transcriptomes between metamorphic stages generated a very low number of DE genes (Additional file 10f–j). For the premetamorphic stage 5, 4,155 transcripts were DE when the transcriptome was compared with the transcriptome for the juvenile stage. In contrast, more than 8,000 transcripts were DE when the transcriptome of whole larvae of each metamorphic stage was compared with the transcriptome of whole juveniles. The majority of the 8,000 DE transcripts per stage were up-regulated in the juvenile stage relative to the metamorphic stages (Fig. 3) and 3,336 of the DE transcripts were common between the metamorphic stages (7, 8, 9A, 9B and 9C). The number of DE transcripts specific to each stage was 403, 365, 1,214, 446 and 362 for stages 7, 8, 9A, 9B and 9C, respectively. The number of DE transcripts common between stages 7 and 8 was 5,999, between stages 8 and 9A was 5,638, between stages 9A and 9B was 5,272 and between 9B and 9C was 5,972.

#### Expression of thyroid related transcripts during metamorphosis

**Fig. 4:**
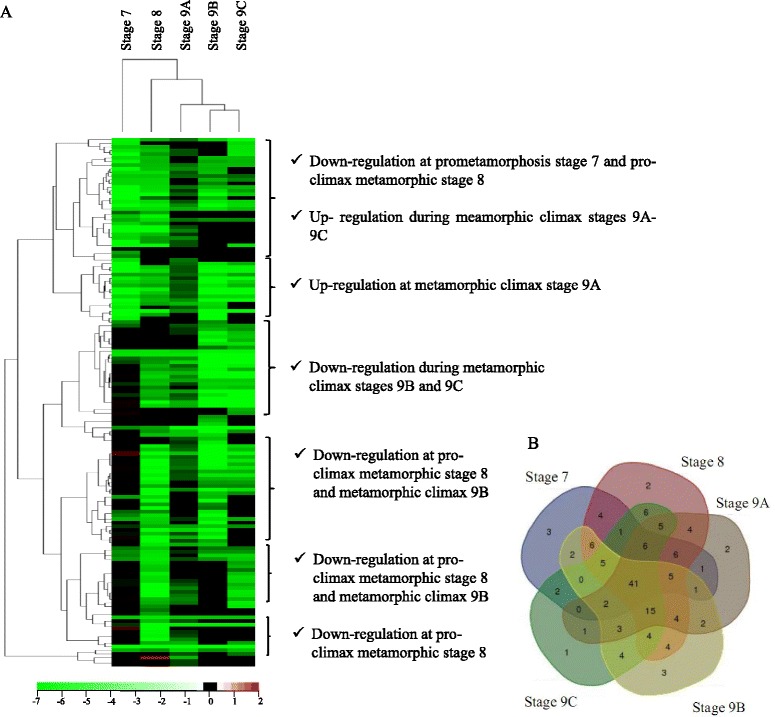
Putative TH-responsive transcripts with differential expression between Atlantic halibut metamorphic stages and juveniles. Clustering of the putative thyroid hormone (TH) responsive transcripts with differential expression between metamorphic stages and juveniles of Atlantic halibut. **a** Heat map of the DE TH-responsive transcripts clustered by expression pattern. Transcript expression is represented as log2 of fold change for metamorphic stages versus juveniles; **b** Venn diagram revealing the number of DE TH-responsive transcripts that are shared between stages or that have a stage specific expression

TH-responsive transcripts DE with SOLiD transcriptional profiling were identified by filtering all differential transcripts using the “in house” database. Overall, 145 putative TH-responsive transcripts were DE (log2 of the fold change of juvenile versus all metamorphic stages), (Fig. 4, detailed information regarding transcripts in Additional files 11 and 12). The majority of the putative TH-responsive transcripts were down-regulated in the metamorphic stages relative to the juvenile (Fig. 4a). The exception was stages 7 and 8 that had 10 and 2 up-regulated putative TH-responsive transcripts, respectively (Additional file 11). Comparison of the putative TH-responsive genes in each stage revealed 41 that were common. Stage 8 had the greatest number of putative TH-responsive transcripts (119), followed by stage 9B (101), stage 9A (98), stage 9C (96) and then stage 7 (85) (Fig. 4c). Enriched reactomes of TH-responsive transcripts during metamorphosis included cellular response to stress, the cell cycle, DNA repair, DNA replication, apoptosis, metabolism, and signal transduction (Additional file 13).

### Up-regulation of TH axis related genes during metamorphic climax

**Fig. 5:**
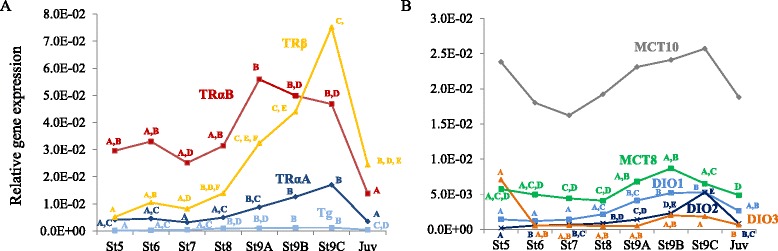
Expression pattern of transcripts involved in the TH cascade during halibut metamorphosis. Schematic representation of the relative gene expression by qPCR of **a** thyroid hormones action (TRαA, TRαB, TRβ) and production (Tg); and **b** thyroid hormones transport (MCT8, MCT10) and regulation of the cellular availability of THs (DIO1, DIO2, DIO3) during Atlantic halibut metamorphosis. Results are presented as relative gene expression (arbitrary units). For detailed information and significance between stages for each transcript, please see Additional file 14

Transcripts that were not DE in SOLiD analysis (presumably due to methodological limitations) but that are involved in the thyroid axis, such as, TH production (thyroglobulin - Tg), transport (monocarboxylated transporter 8 - MCT8, monocarboxylated transporter 10 - MCT10), metabolism (deiodinase 1 - DIO1, deiodinase 2 - DIO2, deiodinase 3 - DIO3) and action (thyroid hormone receptor alpha A - TRαA, thyroid hormone receptor alpha B - TRαB, thyroid hormone receptor beta - TRβ) were analyzed by qPCR using the same samples used for SOLiD analysis (Fig. 5, Additional file 14). Tg transcript levels were lower in the pre-metamorphic stage (stage 5), significantly (*p* < 0.05) higher during metamorphosis, and then decreased significantly (*p* < 0.05) in the post-metamorphic stage (Additional file 14). TRs had a variable expression during metamorphosis and the relative transcript abundance was TRβ > TRαB > TRαA (Fig. 5a). The transcript abundance of all the TRs increased significantly (*p* < 0.05) during metamorphosis. TRαA transcript abundance was significantly higher at metamorphic climax (stages 9B and 9C) relative to stages 5, 6 and 7 and TRαB transcript abundance was also significantly (*p* < 0.05) higher at metamorphic climax (stage 9A) compared to premetamorphic stage 7. The transcript abundance of TRβ throughout metamorphosis (stages, 8, 9A, 9B and 9C) was significantly (*p* < 0.05) higher than during pre-metamorphosis (stages 5 and 7). The transcript abundance of the three TRs was significantly (*p* < 0.05) lower in juveniles relative to metamorphic climax (9C), (Fig. 5a, Additional file 14). The relative gene expression of MCT10 was higher than that of MCT8 throughout metamorphosis, although it did not change significantly at any stage (Fig. 5b, Additional file 14). MCT8 gene expression increased significantly (*p* < 0.05) in stage 9A and 9B relative to stage 8. In juveniles, MCT8 mRNA expression levels were significantly (*p* < 0.05) lower than in metamorphic stages. The gene expression profile of the three deiodinases (DIO1, DIO2 and DIO3) during metamorphosis was similar, and all were significantly (*p* < 0.05) up-regulated during metamorphosis (stage 9A–9C) compared to pre-metamorphic (stage 7 and 8) and juvenile stages (Fig. 5b, Additional file 14).

### Confirmation of differentially expressed transcripts in SOLiD by qPCR

**Fig. 6:**
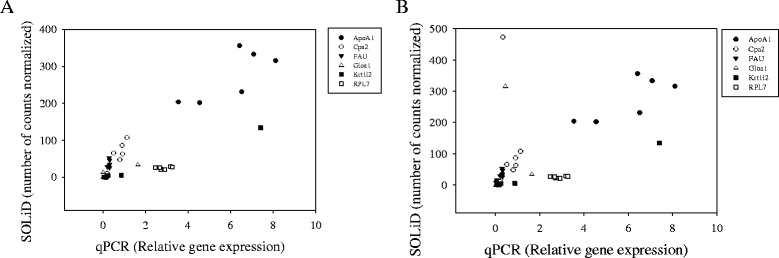
Correlation analysis between SOLiD and qPCR expression analysis. Correlation analyses between SOLiD and qPCR expression of transcripts with a constant expression and transcripts with a modified expression during Atlantic halibut metamorphosis. Comparison of normalized counts (SOLiD data) and relative gene expression profile (qPCR data) of six genes: apolipoprotein A-I (ApoAI), carboxypeptidase A2 (Cpa2), 40S ribosomal protein S30 (FAU), alpha-globin 1 (Gloα1), type I keratin isoform 2 (Krt1i2) and ribosomal protein L7 (RPL7). Different genes are represented by a specific symbol in the graph. Panel **a** Pearson Product Moment Correlation using metamorphic stages 5 to 9C (r = 0.843; p = 1.07 x 10^−7^). Panel **b** A Pearson Product Moment Correlation using metamorphic stages 5 to juvenile (r = 0.576; p = 1.23 x 10^−4^)

Six transcripts from SOLiD analysis of metamorphosing Atlantic halibut were also analyzed by qPCR (Additional file 15) and had a concordant expression pattern (Fig. 6). Transcripts with unchanged transcript abundance during metamorphosis in SOLiD: ribosomal protein L7 (RPL7) and 40S ribosomal protein S30 (FAU) were not significantly different in qPCR. Transcripts, alpha-globin 1 (Gloα1), carboxypeptidase A2 (Cpa2), apolipoprotein AI (ApoAI) and type I keratin isofom 2 (Krt1i2), significantly modified in SOLiD analysis were also significantly (*p* < 0.05) modified in the qPCR results during metamorphosis. A high and significant positive correlation (r = 0.843; p = 1.07 x 10^−7^) was obtained when results of SOLiD analysis for six genes (Gloα1, Cpa2, ApoAI, Krt1i2, RPL7, FAU) were compared with qPCR expression levels (relative to the geometric mean of 40S ribosomal protein S4 and Elongation factor 1 alpha - RPS4/EFIAI) for metamorphic stages 5 - 9C (Fig. 6a). A lower, but significant positive correlation (r = 0.576; p = 1.23 x 10^−4^) was obtained when the juvenile post-metamorphic stage was included in the correlation analysis between SOLiD and qPCR data (Fig. 6b).

## Discussion

Changes during flatfish metamorphosis are not limited to modifications in external morphology but include many structural and functional modifications. The role of TRs as ligand activated TFs means that a significant part of the action of THs on tissues is associated with tissue specific modifications in the transcriptome. In the present study, the large size of Atlantic halibut was utilized to establish for the first time in flatfish specific transcriptomes for larval skin, GI-tract and head during metamorphosis using 454 sequencing. SOLiD sequencing of individual larvae (n = 3/stage) and stage specific comparisons (e.g. 7 vs 8; 8 vs 9A, 9A vs 9B and 9B vs 9C) revealed a very low number of DE transcripts and no sudden or dramatic change between any particular stage. In contrast, pairwise comparisons of the juvenile transcriptome with stage specific transcriptomes revealed a high number of DE transcripts (>8,000/stage), with the majority highly up-regulated in the juvenile stage.

Comparisons of the approximately 8,000 differential transcripts per stage generated a stage specific molecular fingerprint. The large majority of DE transcripts (approx. 98 %) were not classified as TH-responsive and presumably represented transcripts underlying ontogenetic changes and belonging to gene networks that lead to the overt changes that accompany metamorphosis. The latter probably explains why blocking TH action with MMI during flatfish metamorphosis is not lethal and only modifies the development of some specific tissues [22]. In line with this observation THs maintain neoteny in only some of the tissues in salamanders [34–36]. The link between TH-responsive pathways and the numerous other gene networks that change during metamorphosis was not established in the present study, but in future studies will be explored. The majority of the putative DE TH-responsive genes clustered in specific metamorphic stages rather than over the duration of metamorphosis. The response of the majority of putative TH-responsive transcripts was none synchronous with the peak in whole body TH levels, previously reported to occur at stage 9 and 10 for Atlantic halibut [37]. The non-synchronous tissue specific response of putative TH-responsive genes during metamorphosis suggests the chronology of tissue responsiveness during metamorphosis may vary, presumably as a result of differences in cellular responsiveness to THs. Such a phenomenon was reported in a recent study of GI-tract development in the Atlantic halibut [28].

### Metamorphosis-specific tissue transcriptome

Next-generation pyrosequencing 454 technology has been used to characterize the transcriptome from several flatfish species. In turbot (*Scophthalmus maximus*) the study focused on immune related transcripts [38], in the common sole a pooled larval and adult liver and GI-tract transcriptome was established [39] and in Senegalese sole and common sole reference transcriptomes were derived by sequencing several tissue from juveniles and adults [40]. An oligo-array study of common sole development from larva to juvenile revealed a large variety of biological processes occurred during development and that some genes of the thyroid axis were associated with the initiation of metamorphosis [39].

To our knowledge, ours is the first next generation sequencing study analyzing individual tissues and larvae of a metamorphosing flatfish. More than 400,000 sequences per specific-tissue were obtained after filtering to remove poor quality sequences and contaminating transcripts (e.g. prey in the GI-tract *Artemia* sp., etc). This data substantially increases available molecular resources for Atlantic halibut [41, 42]. MIRA3 assembly of the tissue transcriptomes (head: 1,186,541; skin: 830,524 and GI-tract: 418,303) generated 90,676; 65,530 and 38,426 contigs for head, skin and GI-tract, respectively, which was similar to previous studies using the same sequencing strategy [43], but higher than the gene content of the genome of model teleost species e.g. 19,388 for *Takifugu rubripes* and 31,953 for *Danio rerio* (www.ensembl.org). Technical issues, read length and the heuristic nature of the assembly methods, no doubt explain the relatively high transcript number as was previously observed in a liver transcriptome study of *Zoarces viviparus* [44]. Transcript annotation levels in Atlantic halibut were similar to previous 454 studies in turbot, seabream, European eel and silver carp [38, 43, 45, 46]; the contribution of alternative splicing to the high number of assembled transcripts was not established in the study.

### Candidate biological processes and pathways during metamorphosis

Knowledge about the mechanisms underlying the global molecular and cellular changes during fish development, including functional gene annotation during metamorphosis, has significantly increased in the last decade due to genomics and transcriptomics technologies. Previous studies using microarrays, expressed sequence tags (ESTs) and candidate genes in Atlantic halibut identified genes involved in muscle, skin, immune system, signal transduction and transcription factor activity in adult and larvae, but information about larvae undergoing metamorphosis is limited [23, 26, 41, 47]. Studies in *Solea senegalensis* focusing more on metamorphosis generated 10,000 ESTs [48] that are enriched in transcripts involved in the reorganization of somatic tissues, such as, ribosomal proteins, elongation factors and cytoskeletal proteins. The global gene ontology of the individual tissue transcriptomes characterized in the present study, are far more detailed than previous EST studies, but where there is coincident sequence data the results are similar. The GO results of metamorphosing Atlantic halibut is similar to results for other developing teleosts (larval, juvenile and adult) [43, 49], but also between sexes (male and female), whole fish [50] and fish under diverse challenges (e.g. viral challenge) [38], suggesting maintenance of tissue, organ and organism function involves an overwhelming number of common genes that emerge irrespective of the experimental situation. To overcome this problem in the present study we applied a Fisher’s exact test to identify significantly over/under-represented GO terms for the tissue-specific transcriptomes of metamorphosing Atlantic halibut. The 454 transcriptome approach gave insight into tissue specific molecular changes and allied to SOLiD analysis of several individuals/stage revealed core TH-responsive genes responsible for the timing of stage specific responses of individual tissues.

*The metamorphosing Atlantic halibut skin transcriptome:* is enriched in GO terms related to epidermis and connective tissue development, appendage morphogenesis and pigmentation, which is concordant with the morphological modifications observed [51, 52]. Genes involved in vertebrate skin development and morphogenesis are also enriched and include components of the extracellular matrix [ECM, collagen type I (col1a1, col1a2) and type V (col5a2) [53, 54]], ECM remodeling [discoidin domain receptor 1 (ddr1) [55, 56]] and ECM degradation [stromelysin-3 (MMP11), collagenase 3 (mmp13), matrix metallopeptidase 2 (Mmp2) and metalloproteinase inhibitor 3]. Several of the enriched ECM proteins in skin are responsive to THs [13, 57–60] and in SOLiD analysis their DE pattern in stages is asynchronous presumably because of their association with opposing biological processes. The TH-responsive proteins together with the detected TH-independent growth factors, chemokines, adhesion molecules and proteoglycans have previously been identified in relation to tissue differentiation, development and morphogenesis in vertebrate skin [13, 54, 57, 61].

MMP genes identified for the first time in Atlantic halibut skin may be associated with larval-type cell apoptosis during ECM degradation as previously reported in *Xenopus*. For example, collagenase 3 (mmp13) has an important role in *Xenopus* body skin remodelling during metamorphosis [13] and this transcript is present in the halibut larval skin transcriptome and is DE in SOLiD analysis of several individuals/stage with a significant reduction post-metamorphosis. Pathways involved in “focal adhesion” and “tight junction” are also DE in pools of pre-metamorphic *Solea solea* [39]. The enriched transcripts identified during Atlantic halibut metamorphosis that contribute to pigmentation are of practical interest as abnormal pigmentation can have a significant impact on commercial production of flatfish [7, 20, 62, 63].

*The GI-tract in metamorphosing Atlantic halibut*: undergoes extensive remodeling to prepare it for the shift in habitat and diet of the juvenile [28, 64, 65]. SOLiD analysis revealed DE of digestive enzymes, such as trypsin, chymotrypsin and phospholipase A2 during Atlantic halibut metamorphosis, as previously reported in other fish species [29, 66, 67]. Our enriched GO results for GI-tract development in Atlantic halibut are similar to that reported in *Xenopus*, in which GO terms related to digestion are “shut down” at metamorphic climax, but increase again at the end of metamorphosis [68]. The results of the present study corroborate those of a detailed study of GI-tract development in Atlantic halibut that linked up-regulation of pepsinogen and H^+^/K^+^-ATPase α and β subunit with acquisition of a functional proteolytic stomach in early juveniles [28]. However our results diverge from those of an earlier Atlantic halibut microarray study in which genes involved in digestion are more abundant in larvae entering metamorphosis [69], and this may be a consequence of differences in staging, sample composition (pools of larvae were used in previous studies) and the more comprehensive results possible with NGS.

*The head transcriptome*: The enrichment in the head transcriptome of bone related genes ties in with experiments in *Paralichthys lethostigma* in which the development and growth of both sacculus and utricle otoliths are TH dependent during metamorphosis [22]. Alpha-tectorin, otolin and plasma membrane calcium ATPase are also enriched in the Atlantic halibut head transcriptome and a previous candidate gene study suggested they are TH-responsive during flatfish metamorphosis [70]. Several TFs specific for thyroid gland development, such as homeobox protein NK2.1, hematopoietically expressed homeobox (Hhex) and Pax8 are enriched in the Atlantic halibut head transcriptome [71], and suggests that modification of the thyroid tissue is essential for successful metamorphosis [17] and disruption of this process may explain failed metamorphosis in some cases.

*Enriched pathways in metamorphosing Atlantic halibut*: revealed as expected that the essential signaling pathways that trigger tissue development and cell proliferation and differentiation (e.g. Notch, Sonic hedgehog, Wnt, BMP) [72–77], are well represented in all three tissue transcriptomes. This fact lends support to the idea that in Atlantic halibut it is probably not remodeling that gives rise to juvenile tissue but rather *de novo* tissue development during metamorphosis, as has been demonstrated in *Xenopus* [78–80]. Several of the signaling pathways are regulated by THs and specific studies will be required to establish their precise mode of action and the tissue specific consequences of their up-regulation.

*The majority of DE genes detected by SOLiD analysis of several individual halibut larvae per stage:* during metamorphosis are not directly TH-responsive, suggesting that many of the TH effects may be indirect. Cross-referencing of putative TH-responsive genes in whole larvae with the tissue specific transcriptomes provides insight into core tissue changes during metamorphosis. The down-regulation of transcripts linked to the MAPK signalling cascade (c-raf, Kras and C-Jun) during metamorphosis suggests the coordination of TH actions may be via modulation of signalling pathways as has been suggested in *Xenopus* [81]. Similarly, modification in TFs may be another way in which THs bring about an indirect effect. Thus fos-related antigen-2 (fosl2 or FRA-2), sox4, TFPA2, TGFB, HMG1, CEBPD, GTF2H, NFIX and GTF2F that are all TH-responsive [82–85] peaked at metamorphic climax (stage 9A/9B). This is also reminiscent of what occurs during *Xenopus laevis* metamorphosis where TFs have a central role in tissue specific TH-induced programs [86–88].

The reliability of the results of SOLiD DE analysis is generally confirmed by comparison to the results of previous candidate gene studies in fish and amphibians. For example, osteonectin (Sparc), that plays an essential role in tissue morphogenesis [89–91] is strongly down-regulated in stage 7 (log2 fold change, −6.4) but its abundance increases at metamorphic climax stage 9A (log2 fold change, −1.6) and this is reminiscent of what occurs in the flatfish *Scophthalmus maximus* [92]. DE ECM transcripts (alpha2 Collagen type 1 and fibronectin) during Atlantic halibut metamorphosis linked with epidermal outgrowth [93, 94] are also modified in amphibian metamorphosis [86]. The stage specific fingerprint of DE TH-responsive and nonresponsive genes generated by SOLiD analysis of several individuals per stage during Atlantic halibut metamorphosis is a rich resource for future studies of the metamorphic process and its evolution [59]. Furthermore, although in general metamorphosis is comparable between fish and amphibians their divergent evolution, biology and physiology [95] makes flatfish specific data for this process a priority.

### Confirmation of the TH axis role in Atlantic halibut metamorphosis

In flatfish, initiation of metamorphosis is associated with a surge in T4 and T3, which increases up until the metamorphic climax and decreases in post-climax stages [17, 37, 96–99]. The failure to detect by NGS analysis DE genes of the thyroid axis in the present and previous studies of flatfish metamorphosis may be a result of: i) their generally low tissue abundance, which is further aggravated by, ii) the dilution effect caused by using mRNA from whole larvae (or pools of larvae) rather than discrete tissue and iii) the asynchronous temporal expression pattern in different tissues. Nonetheless, Tg transcript abundance detected by qPCR in the present study mirrored the TH profiles in metamorphosing Atlantic halibut [37] and is reminiscent of what occurs in Senegalese sole [100]. Unsurprisingly, transcription of deiodinases (DIO1, DIO2, DIO3), that encode selenoproteins that activate and inactivate THs [8, 101–105] changed during metamorphosis. The results for DIO expression agreed with previous studies of metamorphosis in the Atlantic halibut [26] and Senegalese sole [106]. A limitation of the present study is the impossibility of mapping the spatial and temporal pattern of deiodinase mRNA localization, which is known to be tightly controlled in flounder metamorphosis [107]. The spatial distribution of deiodinases probably contributes to the asynchronous pattern of DE genes during metamorphosis.

In the Atlantic halibut qPCR revealed that the recently identified TH transmembrane transporters (members of the solute carrier (Slc) proteins [108–110]), MCT8 and MCT10, that regulate TH availability in peripheral tissues, are expressed in metamorphosing Atlantic halibut. However, only MCT8, a specific TH transporter [111–113], is significantly up-regulated during the metamorphic climax (stages 9A and 9B) and significantly decreases in post-metamorphic juveniles. The results in Atlantic halibut suggest that in common with metamorphosis in the amphibian (*Xenopus tropicalis*) the tissue distribution and abundance of Slc proteins is one of the factors explaining the differential tissue responsiveness to THs [114–118].

In the Atlantic halibut, TRs had a variable expression pattern during metamorphosis as observed in other flatfish species [18, 37, 95, 119] and this is intriguing when placed in the context of the duality model of TR actions during vertebrate development [120]. In this model, TRα is the predominant TR form during the *Xenopus* larval phase and is associated with repression of TH-inducible genes. Repression of TH-responsive genes occurs when TR and retinoid X receptor (RXR) bind to thyroid response elements (T_3_RE) and in the absence of T3 recruit a co-repressor complex (e.g. Nuclear receptor CoRepressor (NCoR), Silencing Mediator for RAR and TR (SMRT), and other proteins). At metamorphosis the presence of T3 leads to substitution of the co-repressor complex by co-activator proteins and TH-responsive gene transcription is induced. This event is concomitant with TRβ up-regulation (reviewed by Grimaldi et al. [121] and Morvan-Dubois et al. [122]). In Atlantic halibut, our results suggest a dual TR activity model may also exist as TRαB is the main TR form expressed in premetamorphic stages, while TRβ is more abundant at the metamorphic climax. In addition, SOLiD analysis reveals DE of co-activator and repressor elements (NCoR, HDAC1, PRMT1) of TRs during metamorphosis.

In summary, although significant changes in transcripts of the thyroid axis are not detected using SOLiD transcriptome analysis, the temporal expression pattern of DIO, TH transporters and TRs varied dramatically between larvae at different stages confirming the importance of the TH axis in Atlantic halibut metamorphosis [99]. In metamorphosing frogs changes in DIOs, TH transporters and TRs are correlated with the timing of tissue specific changes during metamorphosis [10, 77, 103, 114, 121, 122]. The results of our study and those on frog highlight the importance of analysing individuals and tissues rather than pools of individuals if flatfish metamorphosis is to be understood.

## Conclusions

We report for the first time the tissues specific (skin, GI-tract and head) transcriptomes during metamorphosis of a flatfish species *Hippoglossus hippoglossus* with a high economic value. The study contributes substantially to the molecular resources available for this species and will be an important tool for identifying new potential molecular markers for solving problems related to Atlantic halibut production during metamorphosis. The Atlantic halibut skin transcriptome is a powerful resource for studying the asymmetric pigmentation pattern, as well as the putative cross-talk with the THs axis. Questions relating to the possible asymmetric responsiveness to the THs of both ocular and abocular (blind) sides of skin during metamorphosis remain to be resolved.

The candidate TH-responsive genes identified in the transcriptomes generated will be the subject of future studies to assess tissues responsiveness, and how it is correlated with temporal changes in elements of TH signaling and metabolism during flatfish metamorphosis. Further studies will be essential to identify the tissue specific mechanisms underlying the timing and programming of the developmental events occurring during metamorphosis. The involvement of THs in a late developmental event, metamorphosis, highlights an emerging research area: the regulatory role of hormones in early development.

## Methods

### Sampling

The samples of Atlantic halibut larvae for sequencing were donated by a commercial producer (Fiskeldi Eyjafjarðar Ltd., Iceland) in December 2009. Samples were collected from a standard commercial production cycle [37] undergoing normal metamorphosis by a qualified member of staff and were killed humanely. The samples for analysis were collected using established husbandry procedures and were obtained in the context of routine larval sampling protocols used by the commercial producer to verify the health, welfare and quality of the larvae. The legislation and measures implemented by the commercial producer complied with Directive 98/58/EC (protection of animals kept for farming) and production and sampling conditions were optimised to avoid unnecessary pain, suffering or injury and to maximise larval survival. The study was authorized in accordance with Portuguese legislation for the use of laboratory animals under a Group-1 license from the Direcção-Geral de Veterinária, Ministério da Agricultura, do Desenvolvimento Rural e das Pescas.

After collection the Atlantic halibut larvae (n = 6 per developmental stage) were photographed and staged using myotome height (MH) and standard length (SL) [33]. A further subdivision of stage 9 (9A, 9B, 9C) was made to account for differences in eye migration. Individual larvae were collected into RNAlater (Life Technologies, Carlsbad, USA), gently agitated for 24 h at 4 °C and then transferred to −20 °C for long term storage.

### Transcriptome sequencing

Larvae (stage 5 to 9A–C, n = 6 per stage) were dissected into skin, GI-tract and head. Stage 5 larvae were divided into head and body only. Total RNA was extracted from all tissues and whole individuals (n = 5 per stage) using a Maxwell®16 System (Promega, Madison, USA) following the manufacturer’s instructions. RNA integrity and concentration was verified with an Agilent 2100 Bioanalyzer (Agilent Technologies, Santa Clara, USA) and only samples with RIN values equal to, or above 8 were used. The 454 (GS FLX, Roche Life Sciences, Branford, USA) and SOLiD (AB 5500xl Genetic Analyser system, Applied Biosystems, USA) sequencing was performed at the Max Planck Genome Centre (Cologne, Germany).

#### 454 sequencing

cDNA libraries of the Atlantic halibut skin, GI-tract and head were prepared from pools of 6 samples per stage to obtain 5 μg of total RNA. Ribosomal RNA was depleted using RiboMinus™ Eukaryote Kit (Life Technologies, Carlsbad, USA) and following the manufacturer’s instructions. A cDNA Rapid Library Preparation Kit (Roche Life Sciences, USA) was used to construct sixteen cDNA libraries; head from stage 5 and head, skin and GI-tract from stages 7, 8 and 9A, 9B and 9C. Each library had a unique barcode and was amplified by emulsion PCR and sequenced on a GS-FLX platform (Roche Life Sciences, USA) following the manufacturers recommendations. The sequencing assigned quality scores are available at the NCBI Short Read Archive (SRA; Accession number: SRP044664).

#### SOLiD mRNA sequencing

SOLiD sequencing was carried out on cDNA libraries constructed using SOLiD™ Total RNA-Seq Kit (Applied Biosystems, CA). Ribosomal RNA was depleted from total RNA of whole individual Atlantic halibut extracted as described above. Sixteen cDNA libraries were prepared from individual larvae and included three stage 7, three stage 8, three stage 9A, three stage 9B and two stage 9C libraries from whole individual Atlantic halibut. For the non-metamorphic samples, stage 5 and juvenile, a pool of RNA from 3 individuals was used to prepare the libraries due to sequencing space constraints and cost. Each library had a unique barcode and cDNA was purified to eliminate contaminants, amplified by emulsion PCR and sequenced on a SOLiD AB 5500xl Genetic Analyser platform (Applied Biosystems, USA). The sequencing assigned quality scores are available at the NCBI SRA (Accession number: SRP073364).

### Assembly and annotation

#### 454 sequence reads

Raw sequence reads (.sff format) from the sixteen libraries were extracted and quality clipped and sequencing adapters, primers and poly-A tails were removed. Only sequences above 100 bp were retained for assembly and food source contamination was removed by screening against *Artemia* species available in NCBI (38,287 sequences at 04.2012) and *H. hippoglossus* mitochondrial RNA (27 sequences at 04.2012) was removed using BLASTn (settings: score > 100; e-value <1e-25). After quality filtering and removal of potential contaminants, approximately 70 % of the initial reads from skin (1,200,186) and head (1,556,954) and 43 % from GI-tract (888,165) were kept for assembly. As the number of reads obtained from the stage-specific tissue libraries was highly variable and did not yield robust stage specific comparisons they were combined to produce 3 tissue-specific assemblies. The filtered reads were assembled into contigs using MIRA V3 (http://sourceforge.net/projects/mira-assembler/files/) with the command: mira project = xyx –job = denovo, est, accurate, 454 –DI: trt =/dev/slim, where xyx represent the file extracted from the *sff files of each library [123]. All singletons were discarded. Files containing the reads have been submitted to the National Center for Biotechnology Information Short Read Archive (Accession number: SRP044664; [124]). Validation of library assemblies was performed by Blastx sequence similarity searching (Altschul et al. 1997) against all the available ESTs for Atlantic halibut (21,018 sequences at 07.2014) and by manual annotation of 1 % of randomly chosen contigs from each individual assembly using BLASTx against the Reference Protein database (refseq_protein; NCBI) for vertebrate species.

Annotation of putative function was performed using a multi-step stringent local blast approach adapted from Yúfera et al. [85] (Additional file 1). Sequences were first compared against NCBI *Danio rerio* non-redundant protein database (db) (Blastx; e-value < 1e-20), then against Swissprot (Blastx; e-value < 1e-10) and finally against the non-redundant bony fish protein database (Blastx; e-value < 1e-10). All remaining contigs were then subject to a final Blast search against all the available ESTs for bonyfish (1,191,154 sequences at 2012).

### Functional annotation of 454 tissue transcriptomes

Functional annotation of the assembled 454 tissue transcriptomes was performed using the Blast2GO program v.2.6.0 [125, 126]. Annotated sequences were mapped to gene ontology (GO) terms using the following settings: annotation cut-off: 55; minimum GO weight: 5; and e-value: 1e-06. To augment the GO annotation, ANNEX analyses that gives manually curated univocal relationships between GO terms were also used (https://www.blast2go.com/; [127]). Enrichment of GO terms between different tissues was established using a Fisher’s exact test and applying a False Discovery Rate (FDR) adjusted *p*-value of 0.05. Metabolic KEGG pathway analysis was performed based on the Enzyme Code (EC) obtained for each GO term during the functional annotation step. Each EC was mapped to the corresponding metabolic pathway. The unique and specific tissue GO terms resulting from the 454 tissue transcriptome was analysed with REVIGO (http://revigo.irb.hr/, [128]), which uses a simple clustering algorithm that relies on semantic similarity measures to find representative subsets of GO terms.

#### SOLiD sequence reads

A backbone assembly was created using Newbler and CAP3 (with default parameters) combining all the 454 reads from the different tissues and stages together with the 21,018 ESTs available in public databases (July 2014) to produce 37,073 contigs. The contigs are available at http://ramadda.nerc-bas.ac.uk/repository in the folder: NERC-BAS datasets/Genomics/Transcriptomes/Hippoglossus_hippoglossus. This was then used as the reference for mapping the SOLiD sequences. The paired reads obtained for halibut samples were as follows: 9,532,993 for the pooled stage 5 sample; 11,277,613, 10,533,234 and 9,765,750 for the three stage 7 larvae; 9,658,624, 6,121,383 and 9,518,793 for the 3 stage 8 larvae; 107,948,772, 6,509,721 and 6,343,735 for the 3 stage 9A larvae; 11,101,201, 10,357,984 and 5,282,230 for the three stage 9B larvae; 12,950,027 and 9,195,626 for the two stage 9C larvae and 11,744,701 for the pooled juvenile sample. The reads were mapped to the Atlantic halibut contigs with Maq [129], using default parameters.

Expression analysis was carried out using pairwise comparisons rather than a factorial design. SOLiD sequences that failed to map to contigs were excluded from the analysis. Normalisation was carried out by dividing counts by library size. Differential expression was established using two approaches to increase stringency: a two-fold expression level difference, and the use of a linear model in Bayseq [130], with a Benjamini-Hochberg adjustment for multiple testing [131] with a cut-off set at 0.05. For the linear model, a proxy replication for mapping variance consisted of the separate mappings of the paired end reads to the contigs. Only mappings in which both paired end reads mapped to the same contig were used to generate expression levels and calculate significance of expression. The probable identity of the genes to which SOLiD reads mapped was established by sequence similarity searches using Blast [132], http://blast.ncbi.nlm.nih.gov/Blast.cgi) against the GenBank non redundant database [133] with a threshold score of < 1e-10.

### Identification of tissue-specific and TH-responsive genes

Candidate genes underlying the tissue specific changes (454 transcriptomes and SOLiD analysis) that accompany metamorphosis, were identified by comparing the annotated contigs against 4 in-house databases of genes (setting: e-value ≤ 1e-20), identified using the QuickGO (http://www.ebi.ac.uk/QuickGO/) database and through literature searches. Databases included: 1) database enriched with skin-specific genes, eg. pigmentation, skin development and morphogenesis (141 sequences; Additional file 16); 2) a GI-tract-specific database enriched with genes involved in development, morphogenesis and acid secretion (179 sequences; Additional file 17); 3) genes involved in TH signalling and metabolism and genes involved in thyroid gland development (62 sequences; Additional file 18); 4) TH-responsive genes (189 sequences; Additional file 19).

SOLiD whole larval transcriptome data was used to identify transcripts with differential expression in three individuals per stage by carrying out pairwise comparisons. Two strategies were used for pairwise comparisons: i) pairwise comparisons between metamorphic stages (7, 8, 9A, 9B, 9C) or ii) pairwise comparisons between metamorphic stages (7, 8, 9A, 9B, 9C) and premetamorphic stage 5 larvae or juveniles. Since pairwise comparisons between metamorphic stages (7, 8, 9A, 9B and 9C) yielded very few differential transcripts only the results of the comparison against stage 5 or juveniles was analyzed in detail.

### Quantitative real-time RT-PCR (qPCR)

A set of 16 transcripts was assessed by qPCR, using a subsample of the RNA extracted from whole larvae used for SOLiD transcriptome sequencing. Correlation analysis was carried out between transcripts with differential expression in SOLiD analysis (n = 5) and their expression determined by qPCR. The selected transcripts included genes with a constant expression in all developmental stages, transcripts with modified expression during development and genes involved in the TH cascade (Additional file 20). Specific primers for the target genes were designed based on the cDNA contig sequences (Additional file 20) using Beacon Design and Primer Premier 5.0 software (Premier Biosoft Int., Palo Alto, CA). The reference gene transcripts used to normalize the cDNA used for the PCR reactions were elongation factor I alpha (EF1A1) and 40S ribosomal protein S4 (RPS4) (Additional file 20).

For cDNA synthesis total RNA (10 μg) was first treated with Turbo DNA-Free kit (Ambion, Life Technologies, Carlsbad, USA) to remove contaminating genomic DNA. cDNA synthesis was performed with 500 ng of DNAse treated total RNA, 200 ng of random hexamers (GE Healthcare, Amersham, UK), 100 U of RevertAid M-MuLV Reverse Transcriptase (Fermentas, St Leon-Rot, Germany), 8 U of Ribolock RNase inhibitor (Fermentas, St Leon-Rot, Germany), and 0.5 mM dNTP’s. qPCR reactions were performed in duplicate using SsoFast™ EvaGreen® Supermix (Bio-Rad, Marnes La Coquette, France) chemistry in a StepOnePlus™ Real-Time PCR System (Applied Biosystems, Foster City, USA). The qPCR cycling conditions were: 30 s at 95 °C; 45 cycles of 5 s at 95 °C and 10 s at the optimal temperature for primer pairs (Additional file 18). A final melting curve over a range of 60–95 °C was performed for all reactions. Standard curves relating initial template quantity to amplification cycle were generated from the target gene cloned in pGEM®-T Easy (Promega, Madison, USA) using a 10-fold stepwise serial dilution series (initial concentration, 10^8^ copies amplicon/μl).

The qPCR efficiency for primer pairs ranged from 85 % and 100 % with an R^2^ ≥ 0.99 (Additional file 20). The geometric mean of the reference genes RPS4 and EFIAI was used to normalize the qPCR data. Statistical analysis of the relative gene expression between stages was analyzed by one-way, ANOVA using SigmaPlot v10.0 (Systat Software, Inc., CA, USA) after checking for homogeneity. Tukey’s post-hoc test was used for pair wise multiple comparisons. The expression of transcripts in the halibut stages analyzed is presented as the mean ± standard error of the mean (SEM). Pearson correlation analysis was used to compare the qPCR relative gene expression levels and SOLiD differential count analysis. For correlation analysis six genes from SOLiD analysis with either a constant (RPL7 and FAU) or variable expression (Gloα1, Cpa2, ApoAI, and Krt1i2) during metamorphosis were selected. Statistical significance was established at *p* < 0.05.

### Ethics and consent to participate

All experimental procedures involving animals complied with the Directive 98/58/EC (protection of animals kept for farming) and were authorized in accordance with Portuguese legislation for the use of laboratory animals under a Group-1 license from the Direcção-Geral de Veterinária, Ministério da Agricultura, do Desenvolvimento Rural e das Pescas.

### Consent to publish

Not applicable

### Availability of data and material

The 454 sequences for Atlantic halibut obtained in this study are available at the NCBI SRA under the accession number: SRP044664 and the consensus sequences of the contigs are available at http://ramadda.nerc-bas.ac.uk/repository in the folder: NERC-BAS datasets/Genomics/Transcriptomes/Hippoglossus_hippoglossus. All SOLiD sequence data were submitted to the NCBI SRA with the accession number: SRP073364.

The gene acronyms and respective full names are given in Additional files 15-18.

## Additional files

Additional file 1: Scheme of the data processing pipeline for *de novo* transcriptome assembly, annotation and Gene Ontology analysis of Atlantic halibut skin, GI-tract and head transcriptomes. (PDF 263 kb) Additional file 2: Annotation of the skin transcriptome. Contig ID, transcript name, number of GOs and their description and enzymatic codes are shown for each contig. (XLSX 3090 kb) Additional file 3: Annotation of the GI-tract transcriptome. Contig ID, transcript name, number of GOs and their description and enzymatic codes are shown for each contig. (XLSX 1784 kb) Additional file 4: Annotation of the head transcriptome. Contig ID, transcript name, number of GOs and their description and enzymatic codes are shown for each contig. (XLSX 4280 mb) Additional file 5: Schematic representation of the functional annotation obtained after analysis of the transcriptomes for skin, GI-tract and head. The GO terms (level 2) used for classification were biological process (BP), molecular function (MF) and cellular component (CC). (PDF 107 kb) Additional file 6: Significantly overrepresented Biological Process GO terms identified for the skin transcriptome (FDR < 0.05). (DOC 89 kb) Additional file 7: Significantly overrepresented Biological Process GO terms identified for the GI-tract transcriptome (FDR < 0.05). (DOC 71 kb) Additional file 8: Significantly overrepresented Biological Process GO terms identified for the head transcriptome (FDR < 0.05). (DOC 116 kb) Additional file 9: List of the most representative metabolic pathways in the skin, GI-tract and head transcriptomes using KEGG analysis. (DOCX 16 kb) Additional file 10: The list of the top most significantly up-regulated genes between premetamorphic stage 5 and the Juvenile stage determined using a linear model in Bayseq with a Benjamin-Hochberg adjustment for multiple testing analysis with a cut-off set at 0.05 (FDR < 0.05). Contig name, Gene name, Accession number (no.), Organism and E-value are shown for each gene. (DOCX 75 kb) Additional file 11: Expression profile of putative TH-responsive transcripts that had a differential expression between Atlantic halibut metamorphic stages (7–9C) and the juvenile. Expression levels are represented as log2 of fold-change (juvenile/metamorphic stages expression levels). (DOCX 41 kb) Additional file 12: Heat map with the expression profile (log2 of fold-change) of putative thyroid hormones (TH) responsive transcripts with differential expression between juvenile and metamorphic stages of Atlantic halibut. (PDF 312 kb) Additional file 13: Reactome pathway analysis for the 145 differential expressed TH-responsive transcripts identified when Atlantic halibut metamorphic stages were compared with the juvenile. Reactome analysis was performed using INTREPROSCAN accession numbers obtained from the functional annotation of the Atlantic halibut transcriptome with Blast2GO. (DOCX 18 kb) Additional file 14: Relative gene expression analysis of transcripts involved in the TH cascade during Atlantic halibut development (stage 5 to juvenile; n = 5 per stage) using quantitative RT-PCR (qPCR). Thyroglobulin (Tg), TH receptor alpha A (TRαA), TH receptor alpha B (TRαB), TH receptor beta (TRβ), deiodinase 3 (DIO3), deiodinase 2 (DIO2), deiodinase 1 (DIO1), monocarboxylate transporter 8 (MCT8), and monocarboxylate transporter 10 (MCT10) gene expression. Results are presented as mean ± SEM of the candidate gene expression, normalized using the geometric mean of the reference genes RPS4 and EF1A1. Significant difference (*p* < 0.05; one-way ANOVA) of normalized transcript expression between stages are indicated by different letters. (PDF 200 kb) Additional file 15: Quantitative RT-PCR (qPCR) of the relative expression of ribosomal protein L7 (RPL7), 40S ribosomal protein S30 (FAU); alpha-globin 1 (Gloα1), carboxypeptidase A2 (Cpa2), apolipoprotein A-I (ApoAI) and type I keratin isoform 2 (Krt1i2). Analysis of the indicated transcripts was performed in whole Atlantic halibut larvae during development (stage 5 to juvenile; n = 5). The results are presented as mean ± SEM of the normalized expression, using the geometric mean of the reference genes RPS4 and EFIAI. Different letters represent significantly different mean values (p < 0.05; one-way ANOVA). (PDF 188 kb) Additional file 16: In-house skin-specific database enriched with candidate genes involved in vertebrate skin development, morphogenesis and pigmentation. Protein name, Symbol, Accession number (no.), Organism and Biological role are shown. (XLSX 19 kb) Additional file 17: In house GI-tract-specific database enriched with genes involved in GI-tract development, morphogenesis and acid secretion. Protein name, Symbol, Accession number (no.), Organism and Biological role (when associated) are shown. (XLSX 24 kb) Additional file 18: In-house database of candidate genes involved in thyroid gland development/thyroid hormone (TH) metabolism and signaling, including transcripts with a relevant role in TH synthesis, transport and activity. Protein name, Symbol, Accession number (no.), Organism and Biological role are shown. (XLSX 13 kb) Additional file 19: In-house database of candidate genes involved in TH signaling/metabolism identified in *Xenopus laevis* in previously published literature [84, 86, 87, 134]. Protein name, Symbol, Accession number (no.), Organism and References are shown. (XLSX 29 kb) Additional file 20: Specific primers used for qPCR gene expression analysis. Gene symbol, name and function are shown. The annealing temperature (Ta °C), amplicon length (bp), R^2^ and qPCR efficiency (%) are indicated for each primer pair. (DOCX 17 kb)

## Abbreviations

ASAB: Association of Animal Behaviour
Blast: Basic local alignment search tool
Bmp: Bone morphogenic protein
BP: Biological process
CC: Cellular component
DE: Differentially expressed qPCR, Quantitative real-time polymerase chain reaction
ECM: extracellular matrix
ESTs: Expressed sequence tags
FDR: False discovery rate
GI-tract: Gastrointestinal tract
GO: Gene ontology
KEGG: Kyoto encyclopedia of genes and genomes
MF: Molecular function
MH: Myotome height
MMI: Methimazole
MMP: Matrix metalloproteinase
NCBI: National Center for Biotechnology Information
NGS: Next-generation sequencing technology
REVIGO: Reduce + Visual Gene Ontology
RXR: retinoid X receptor
SEM: standard error of the mean
Shh: Sonic Hedgehog
SL: Standard length
SLC: solute carrier
SRA: Short Read Archive
T3: triiodothyronine
T4: Thyroxin
TFs: transcription factors
TGFβ: transforming growth factor beta
THs: Thyroid hormones
