# Supplementary material for: The transcriptome of metamorphosing flatfish
Source: BMC Genomics. 2016 May 27;17:413. doi: 10.1186/s12864-016-2699-x (PMC4884423; doi:10.1186/s12864-016-2699-x)
Supplement: Additional file 1: — Scheme of the data processing pipeline for de novo transcriptome assembly, annotation and Gene Ontology analysis of Atlantic halibut skin, GI-tract and head transcriptomes. (PDF 263 kb) [file 12864_2016_2699_MOESM1_ESM.pdf]

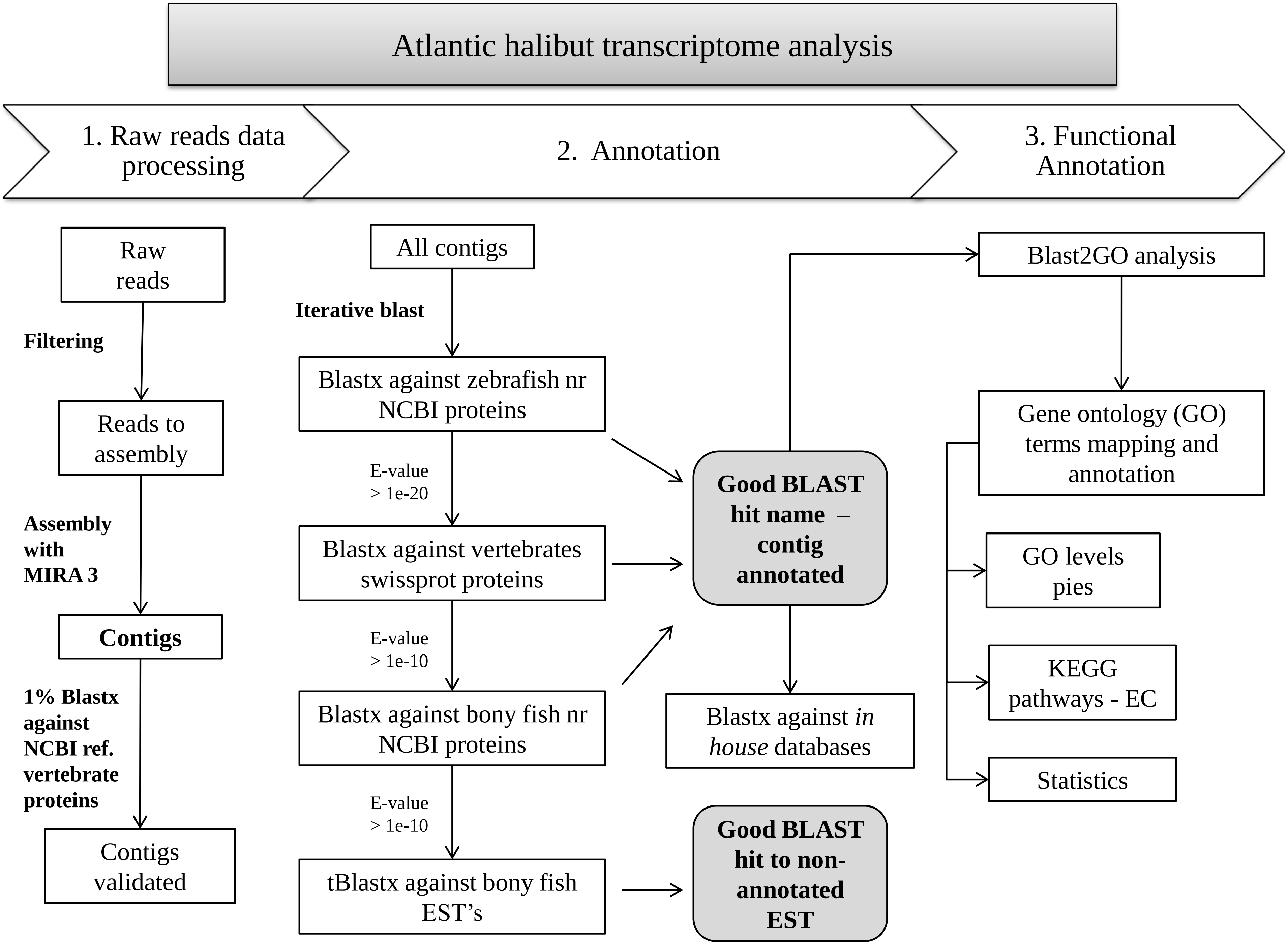
Atlantic halibut transcriptome analysis
1. Raw reads data processing
2. Annotation
3. Functional Annotation
Raw reads
Filtering
Reads to assembly
Assembly with MIRA 3
Contigs
1% Blastx against NCBI ref. vertebrate proteins
Contigs validated
All contigs
Iterative blast
Blastx against zebrafish nr NCBI proteins
E-value > 1e-20
Blastx against vertebrates swissprot proteins
E-value > 1e-10
Blastx against bony fish nr NCBI proteins
E-value > 1e-10
tBlastx against bony fish EST's
Good BLAST hit name – contig annotated
Blastx against in house databases
Good BLAST hit to non-annotated EST
Blast2GO analysis
Gene ontology (GO) terms mapping and annotation
GO levels pies
KEGG pathways - EC
Statistics
