# Supplementary figures and images for: The transcriptome of metamorphosing flatfish
Source: BMC Genomics. 2016 May 27;17:413. doi: 10.1186/s12864-016-2699-x (PMC4884423; doi:10.1186/s12864-016-2699-x)

Biological Process (BP)

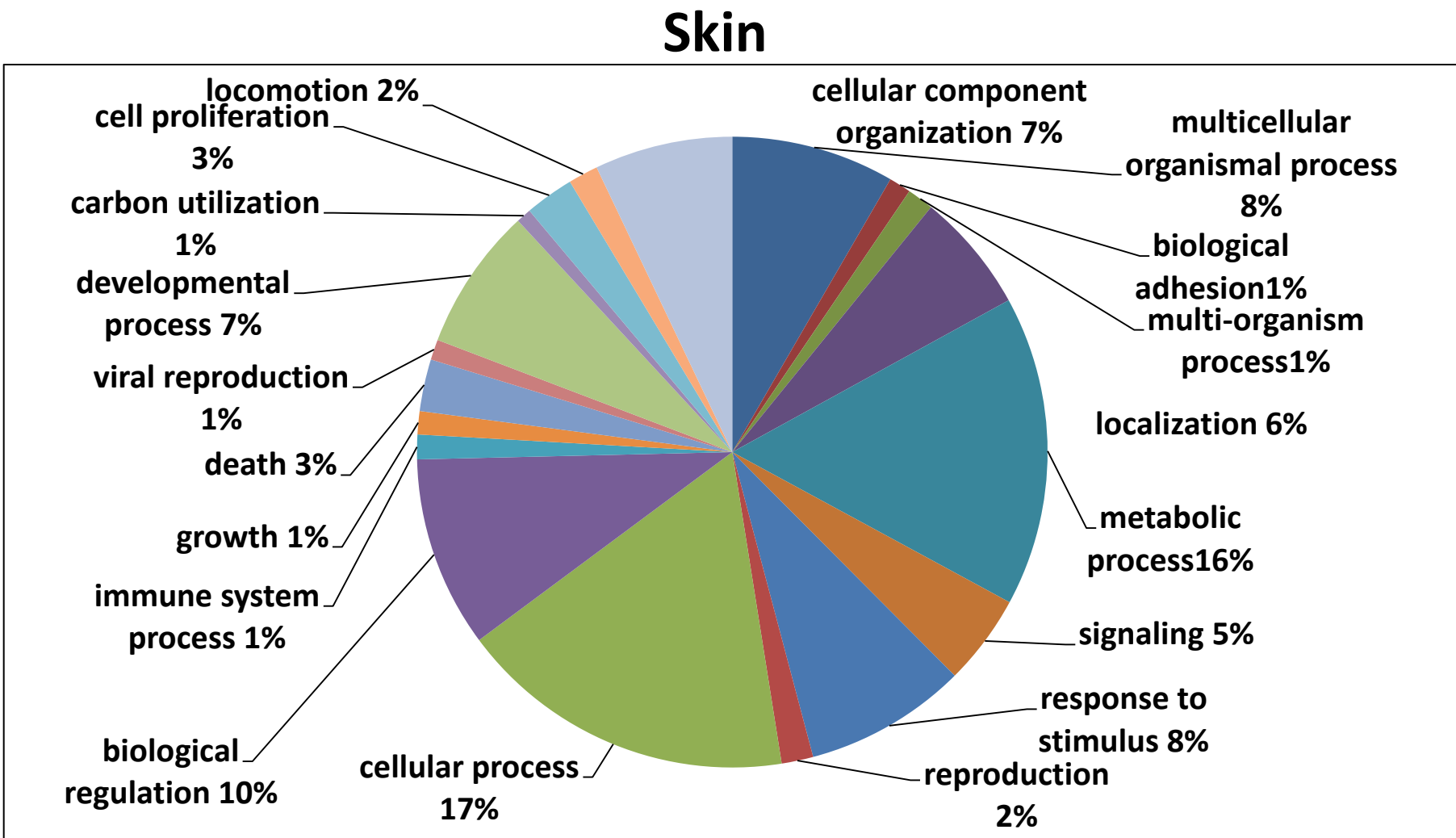

GI-tract

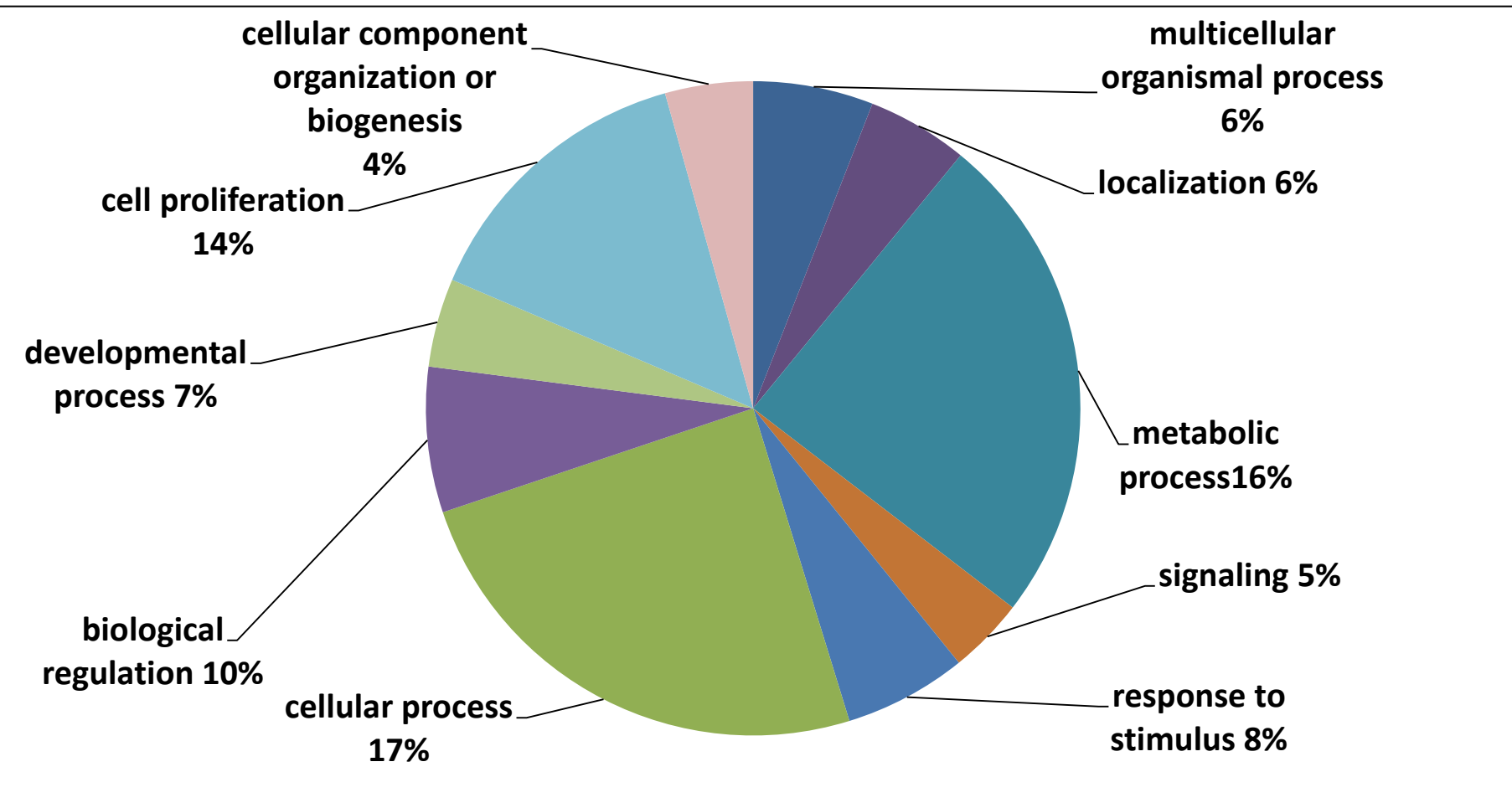

Head

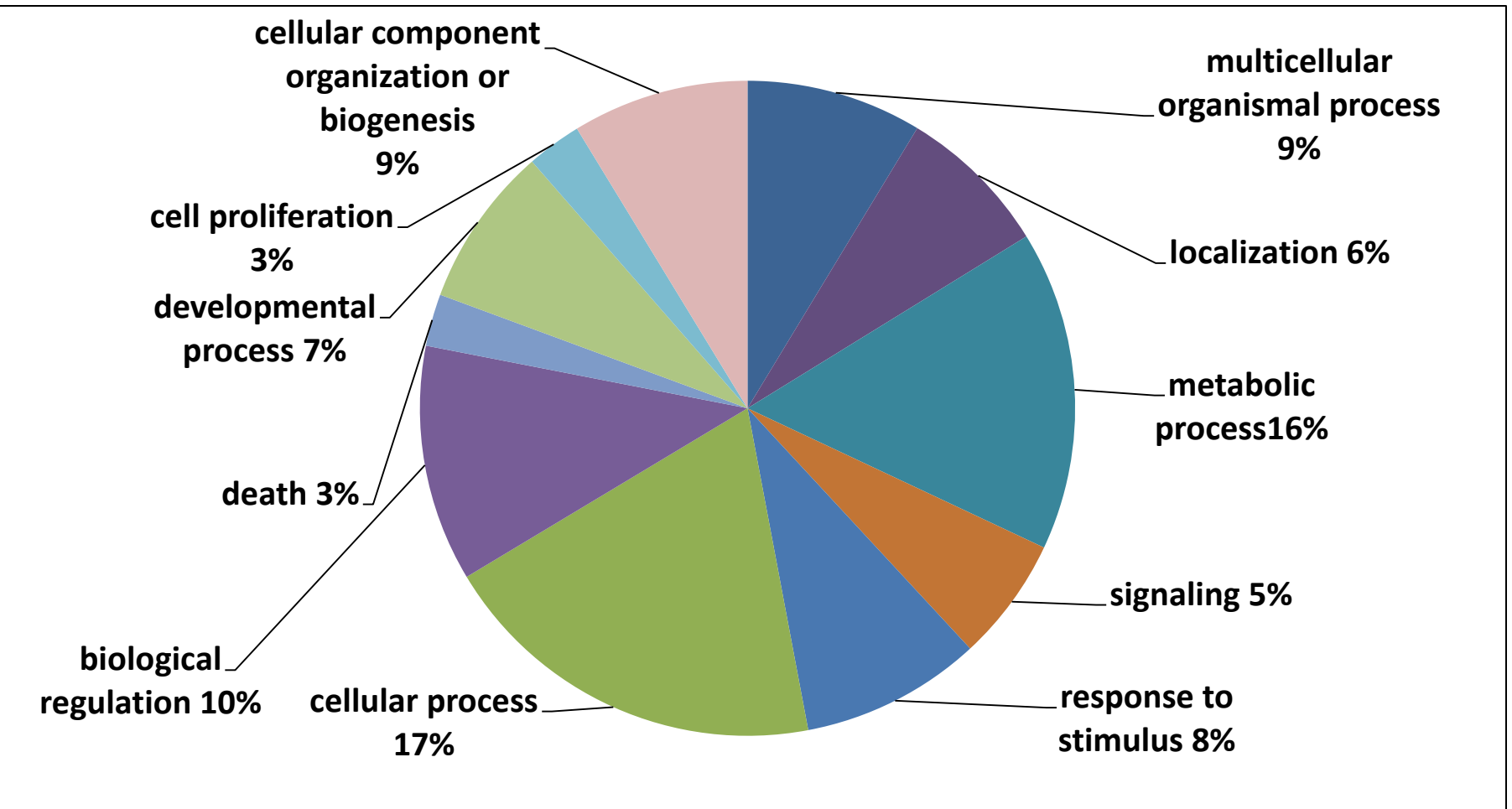

Molecular Function (MF)

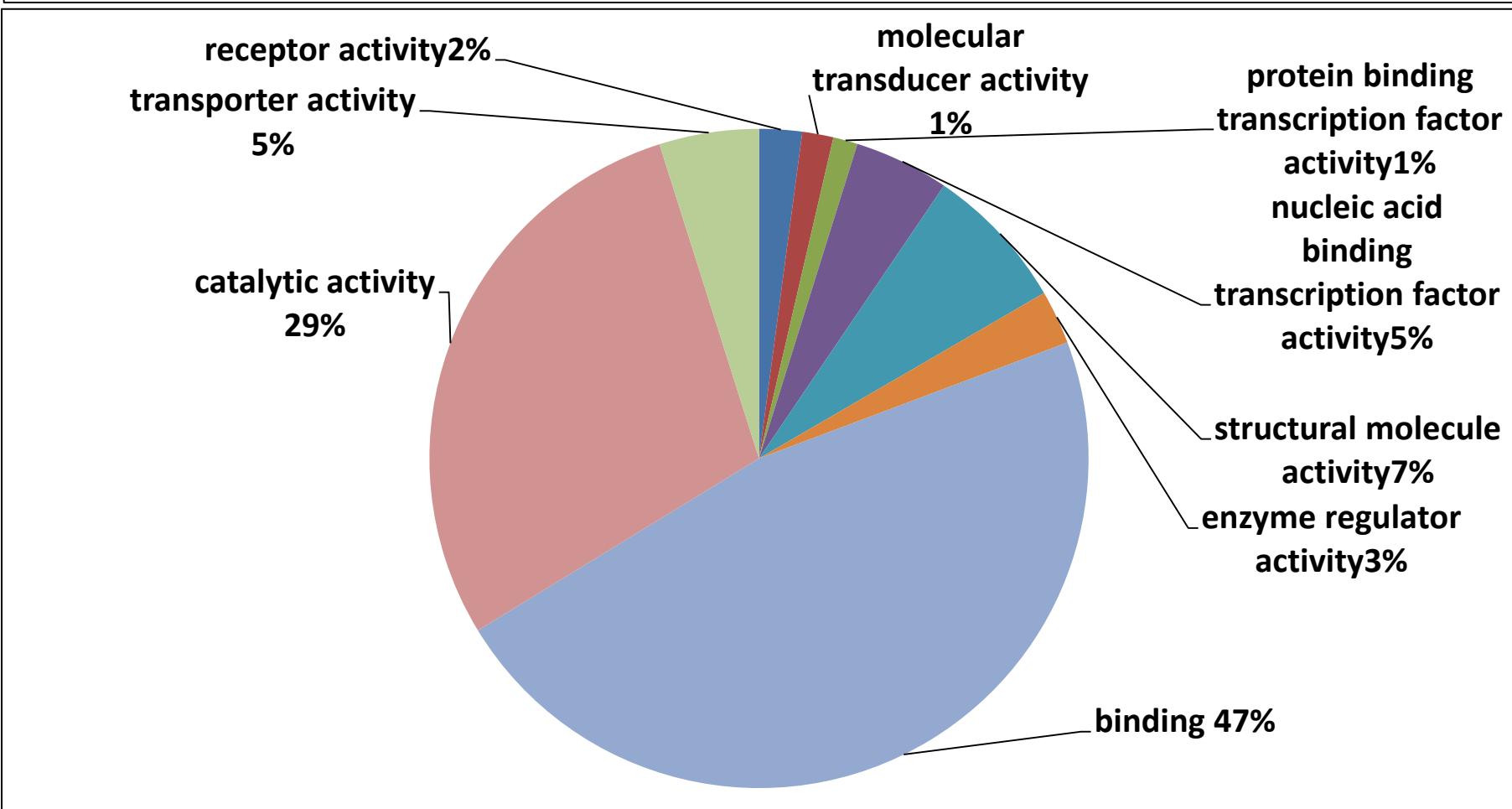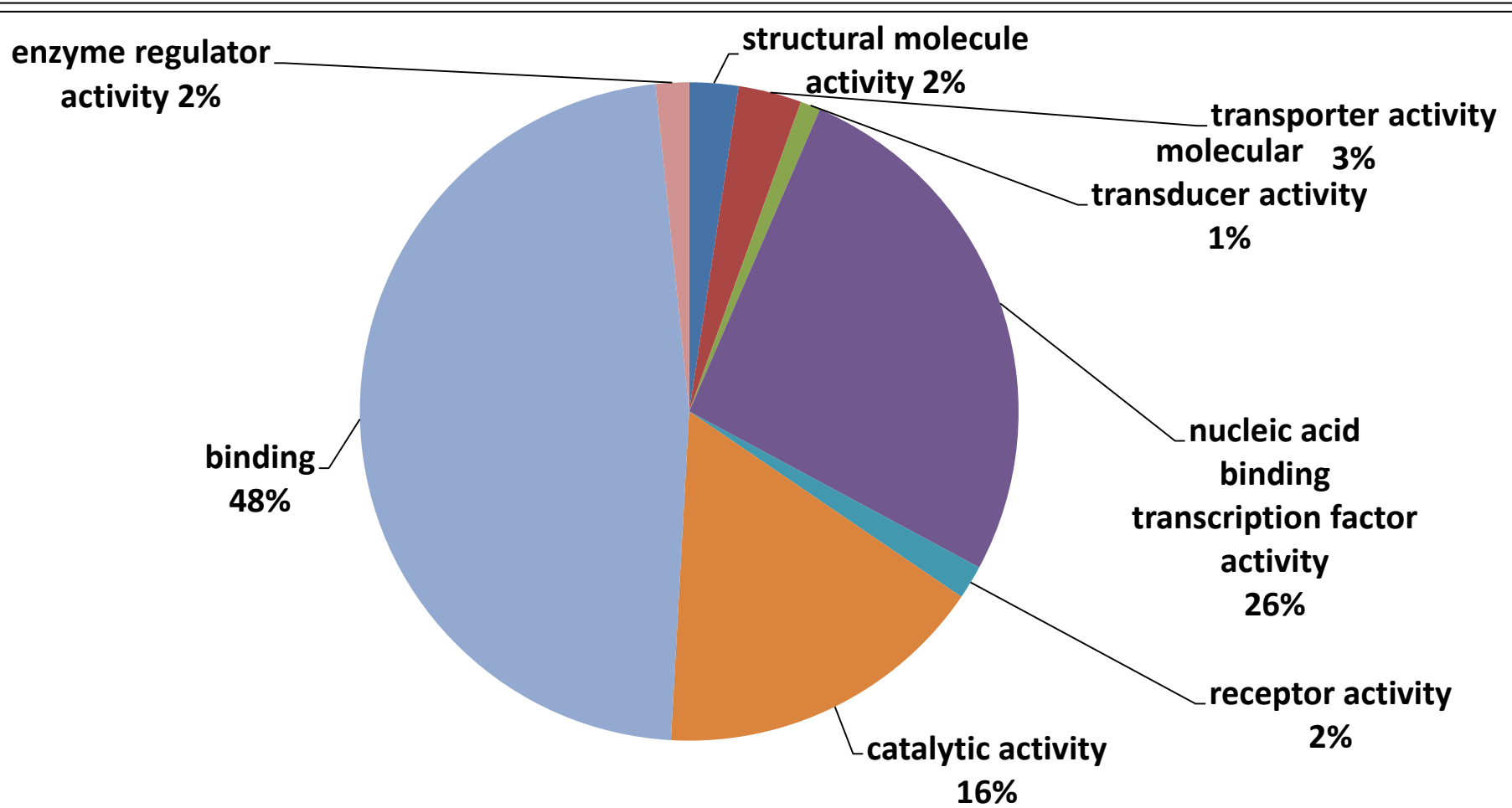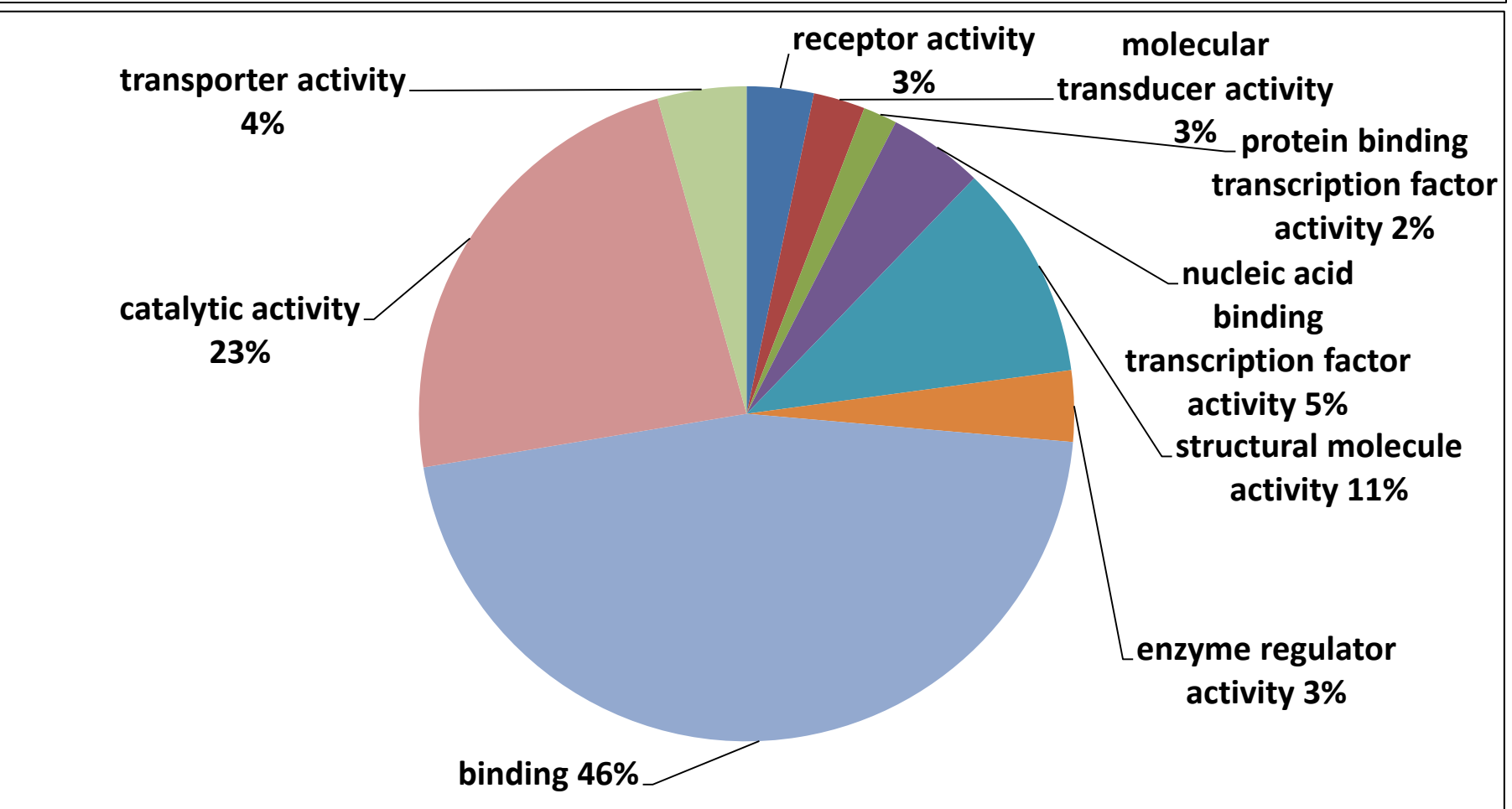

Cellular Component (CC)

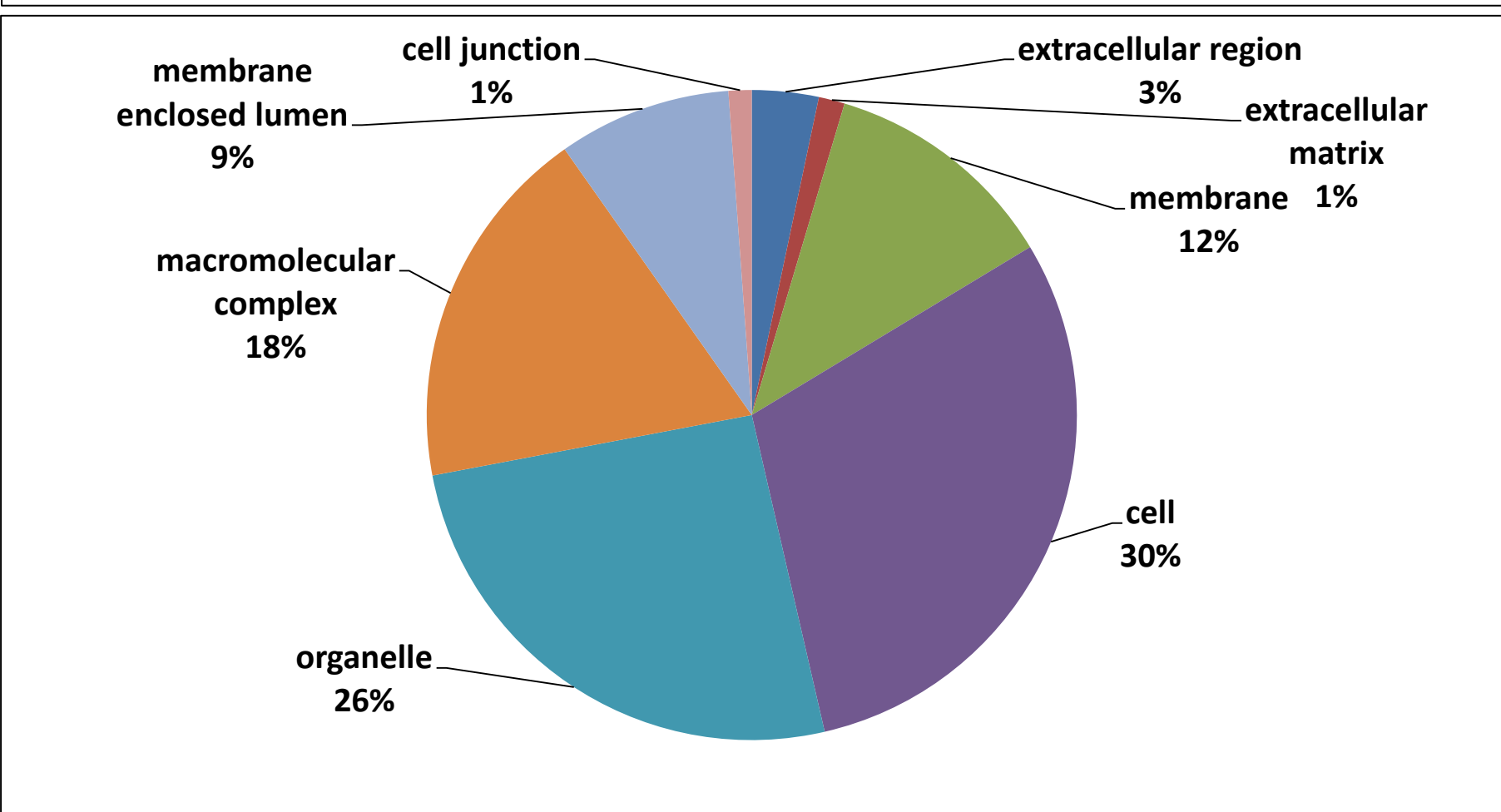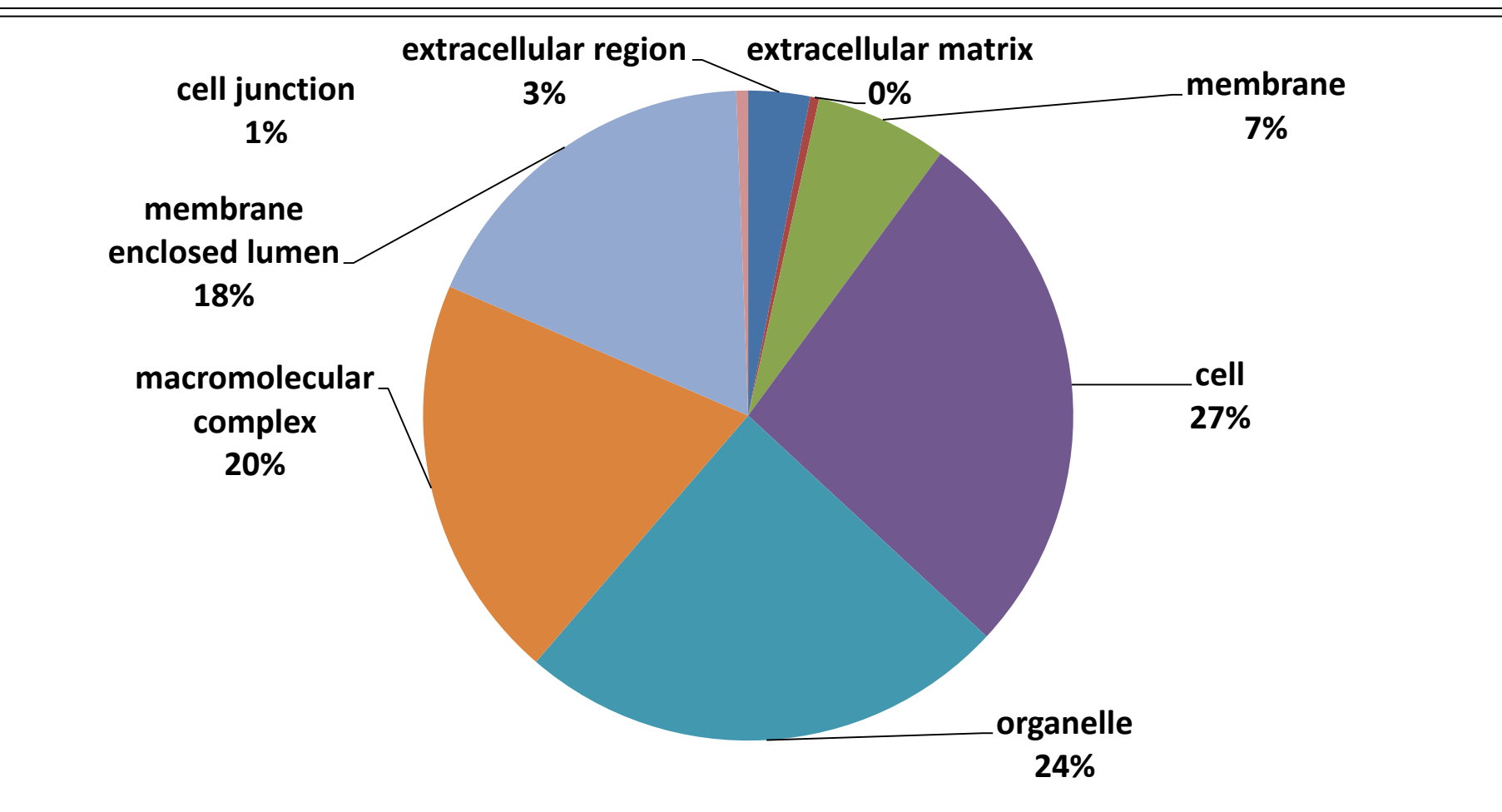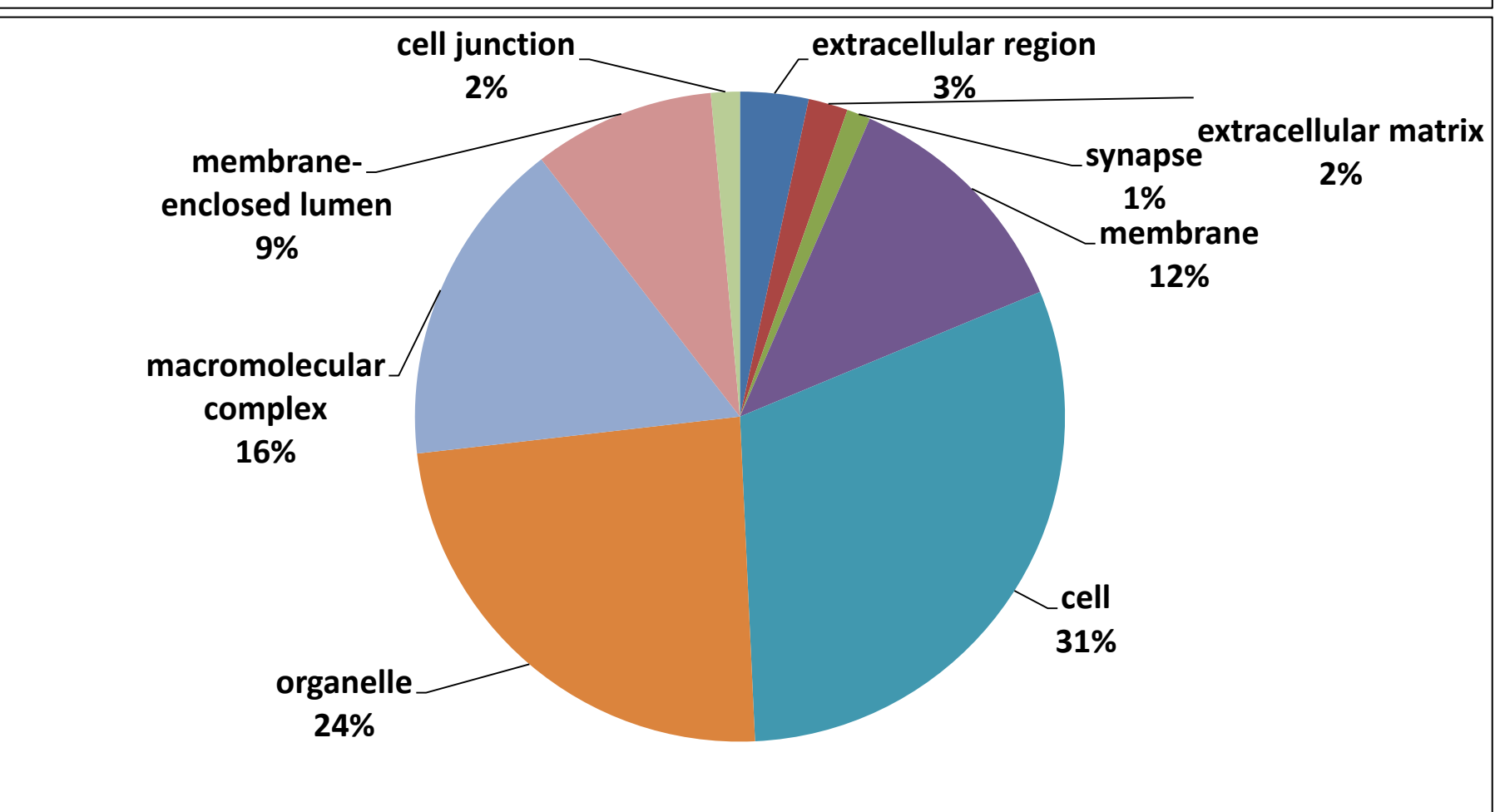

Supplement: Additional file 5: — Schematic representation of the functional annotation obtained after analysis of the transcriptomes for skin, GI-tract and head. The GO terms (level 2) used for classification were biological process (BP), molecular function (MF) and cellular component (CC). (PDF 107 kb) [file 12864_2016_2699_MOESM5_ESM.pdf]

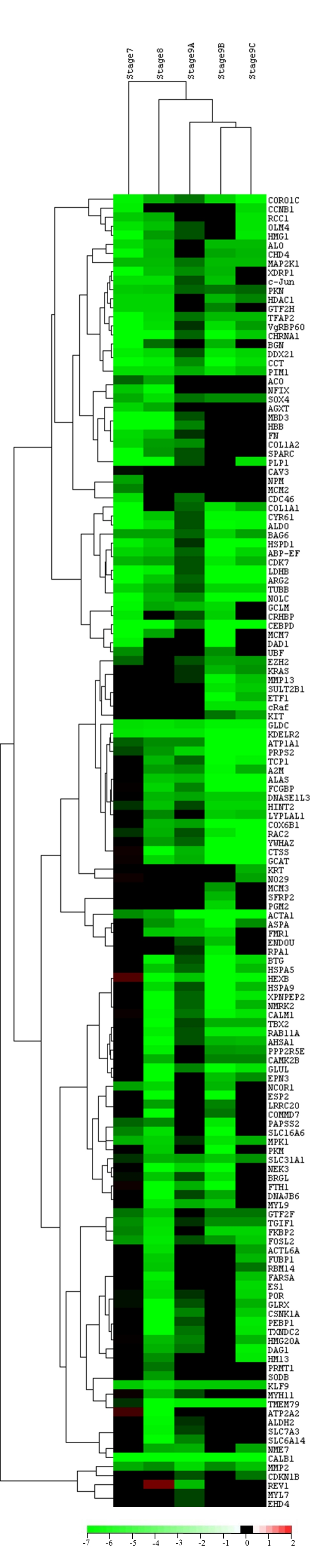

Supplement: Additional file 12: — Heat map with the expression profile (log2 of fold-change) of putative thyroid hormones (TH) responsive transcripts with differential expression between juvenile and metamorphic stages of Atlantic halibut. (PDF 312 kb) [file 12864_2016_2699_MOESM12_ESM.pdf]
