# Supplementary material for: The transcriptome of metamorphosing flatfish
Source: BMC Genomics. 2016 May 27;17:413. doi: 10.1186/s12864-016-2699-x (PMC4884423; doi:10.1186/s12864-016-2699-x)
Supplement: Additional file 6: — Significantly overrepresented Biological Process GO terms identified for the skin transcriptome (FDR < 0.05). (DOC 89 kb) [file 12864_2016_2699_MOESM6_ESM.doc]

**Additional file 6: Selected significantly overrepresented Biological Process GO terms in the skin transcriptome (FDR<0.05).**

| **Overrepresented in relation to head** | | | | | **Overrepresented in relation to GI tract** | | |
| --- | --- | --- | --- | --- | --- | --- | --- |
| ***GO term ID*** | | ***GO term description*** | | ***FDR*** | ***GO term ID*** | ***GO term description*** | ***FDR*** |
| GO:0003012 | | Muscle system process | | 0 | GO:3012 | Muscle system process | 0 |
| GO:0006936 | | Muscle contraction | | 0 | GO:3008 | System process | 0 |
| GO:0006941 | | Striated muscle contraction | | 2.68E-308 | GO:6941 | Striated muscle contraction | 0 |
| GO:0006096 | | Glycolysis | | 1.27E-265 | GO:6936 | Muscle contraction | 0 |
| GO:0006094 | | Gluconeogenesis | | 3.24E-265 | GO:71842 | Cellular component organization at cellular level | 0 |
| GO:0019319 | | Hexose biosynthetic process | | 6.81E-262 | GO:71841 | Cellular component organization or biogenesis at cellular level | 0 |
| GO:0046364 | | Monosaccharide biosynthetic process | | 2.01E-257 | GO:71840 | Cellular component organization or biogenesis | 0 |
| GO:0006007 | | Glucose catabolic process | | 2.07E-253 | GO:7275 | Multicellular organismal development | 0 |
| GO:0019320 | | Hexose catabolic process | | 6.52E-250 | GO:65007 | Biological regulation | 0 |
| GO:0046365 | | Monosaccharide catabolic process | | 3.57E-248 | GO:32502 | Developmental process | 0 |
| GO:0006006 | | Glucose metabolic process | | 1.60E-232 | GO:32501 | Multicellular organismal process | 0 |
| GO:0006754 | | ATP biosynthetic process | | 8.51E-228 | GO:48856 | Anatomical structure development | 0 |
| GO:0016051 | | Carbohydrate biosynthetic process | | 8.90E-224 | GO:50896 | Response to stimulus | 0 |
| GO:0016052 | | Carbohydrate catabolic process | | 1.36E-222 | GO:50794 | Regulation of cellular process | 0 |
| GO:0019318 | | Hexose metabolic process | | 6.30E-217 | GO:50789 | Regulation of biological process | 0 |
| GO:0009201 | | Ribonucleoside triphosphate biosynthetic process | | 6.85E-216 | GO:9653 | Anatomical structure morphogenesis | 0 |
| GO:0009145 | | Purine nucleoside triphosphate biosynthetic process | | 1.08E-215 | GO:9056 | Catabolic process | 0 |
| GO:0009206 | | Purine ribonucleoside triphosphate biosynthetic process | | 1.08E-215 | GO:16043 | Cellular component organization | 0 |
| GO:0009142 | | Nucleoside triphosphate biosynthetic process | | 9.10E-214 | GO:6807 | Nitrogen compound metabolic process | 0 |
| GO:0005996 | | Monosaccharide metabolic process | | 9.48E-211 | GO:6725 | Cellular aromatic compound metabolic process | 0 |
| GO:0009152 | | Purine ribonucleotide biosynthetic process | | 3.95E-209 | GO:71704 | Organic substance metabolic process | 0 |
| GO:0009260 | | Ribonucleotide biosynthetic process | | 1.13E-204 | GO:6139 | Nucleobase-containing compound metabolic process | 0 |
| GO:0001757 | | Somite specification | | 5.78E-195 | GO:44260 | Cellular macromolecule metabolic process | 0 |
| GO:0007379 | | Segment specification | | 1.05E-187 | GO:44238 | Primary metabolic process | 0 |
| GO:0015976 | | Carbon utilization | | 8.34E-180 | GO:9058 | Biosynthetic process | 0 |
| GO:0019438 | | Aromatic compound biosynthetic process | | 1.60E-178 | GO:34641 | Cellular nitrogen compound metabolic process | 0 |
| GO:0006164 | | Purine nucleotide biosynthetic process | | 2.20E-175 | GO:46483 | Heterocycle metabolic process | 0 |
| GO:0031448 | | Positive regulation of fast-twitch skeletal muscle fiber contraction | | 6.35E-175 | GO:6950 | Response to stress | 3.51E-289 |
| GO:0031446 | | Regulation of fast-twitch skeletal muscle fiber contraction | | 6.35E-175 | GO:55086 | Nucleobase-containing small molecule metabolic process | 1.70E-285 |
| GO:0014724 | | Regulation of twitch skeletal muscle contraction | | 6.35E-175 | GO:9117 | Nucleotide metabolic process | 9.75E-278 |
| GO:0009165 | | Nucleotide biosynthetic process | | 1.03E-174 | GO:6753 | Nucleoside phosphate metabolic process | 2.95E-274 |
| GO:0018130 | | Heterocycle biosynthetic process | | 1.28E-173 | GO:6096 | Glycolysis | 1.02E-263 |
| GO:0090407 | | Organophosphate biosynthetic process | | 2.63E-172 | GO:72521 | Purine-containing compound metabolic process | 8.60E-257 |
| GO:0072522 | | Purine-containing compound biosynthetic process | | 1.35E-170 | GO:6796 | Phosphate-containing compound metabolic process | 2.71E-256 |
| GO:0031443 | | Fast-twitch skeletal muscle fiber contraction | | 4.07E-170 | GO:6793 | Phosphorus metabolic process | 2.71E-256 |
| GO:0034654 | | Nucleobase-containing compound biosynthetic process | | 1.68E-168 | GO:44281 | Small molecule metabolic process | 2.19E-255 |
| GO:0045988 | | Negative regulation of striated muscle contraction | | 9.15E-167 | GO:8152 | Metabolic process | 1.98E-254 |
| GO:0014819 | | Regulation of skeletal muscle contraction | | 1.85E-165 | GO:9205 | Purine ribonucleoside triphosphate metabolic process | 1.27E-253 |
| GO:0045989 | | Positive regulation of striated muscle contraction | | 1.98E-159 | GO:9144 | Purine nucleoside triphosphate metabolic process | 3.74E-252 |
| GO:0045932 | | Negative regulation of muscle contraction | | 1.98E-159 | GO:9199 | Ribonucleoside triphosphate metabolic process | 1.32E-251 |
| GO:0031100 | | Organ regeneration | | 0.006 | GO:50881 | Musculoskeletal movement | 6.57E-156 |
| GO:0048729 | | Tissue morphogenesis | | 0.022 | GO:8544 | Epidermis development | 1.10E-25 |
|  | | | | | GO:61448 | Connective tissue development | 1,44E-23 |
|  |  | |  | | GO:35107 | Appendage morphogenesis | 5.59E-22 |
|  |  | |  | | GO:2520 | Immune system development | 5.27E-05 |
|  |  | |  | | GO:72331 | Signal transduction by p53 class mediator | 0.0061 |
|  |  | |  | | GO:14033 | Neural crest cell differentiation | 0.0062 |
|  |  | |  | | GO:43589 | Skin morphogenesis | 1.41E-02 |
|  |  | |  | | GO:43588 | Skin development | 1.71E-02 |
|  |  | |  | | GO:2764 | Immune response-regulating signaling pathway | 0.0239 |
|  |  | |  | | GO:2757 | Immune response-activating signal transduction | 0.0249 |
|  |  | |  | | GO:2684 | Positive regulation of immune system process | 0.0273 |
|  |  | |  | | GO:48066 | Developmental pigmentation | 0.0297 |
|  |  | |  | | GO:9725 | Response to hormone stimulus | 4.26E-70 |
|  |  | |  | | GO:48545 | Response to steroid hormone stimulus | 2.19E-58 |
|  |  | |  | | GO:43434 | Response to peptide hormone stimulus | 4.98E-26 |
|  |  | |  | | GO:30518 | Intracellular steroid hormone receptor signaling pathway | 0.0014 |
|  |  | |  | | GO:71375 | Cellular response to peptide hormone stimulus | 0.0164 |
|  |  | |  | | GO:71383 | Cellular response to steroid hormone stimulus | 0.0216 |
