# Supplementary material for: The transcriptome of metamorphosing flatfish
Source: BMC Genomics. 2016 May 27;17:413. doi: 10.1186/s12864-016-2699-x (PMC4884423; doi:10.1186/s12864-016-2699-x)
Supplement: Additional file 7: — Significantly overrepresented Biological Process GO terms identified for the GI-tract transcriptome (FDR < 0.05). (DOC 71 kb) [file 12864_2016_2699_MOESM7_ESM.doc]

**Additional file 7:** Selected significantly overrepresented Biological Process GO terms in the GI tract transcriptome (FDR<0.05).

| **Overrepresented in relation to head** | | | **Overrepresented in relation to skin** | | |
| --- | --- | --- | --- | --- | --- |
| ***GO term ID*** | ***GO term description*** | ***FDR*** | ***GO term ID*** | ***GO term description*** | ***FDR*** |
| GO:0032774 | RNA biosynthetic process | 0 | GO:32774 | RNA biosynthetic process | 0 |
| GO:0006351 | Transcription, DNA-dependent | 0 | GO:6351 | Transcription, DNA-dependent | 0 |
| GO:0009303 | Rrna transcription | 0 | GO:90304 | Nucleic acid metabolic process | 0 |
| GO:0008283 | Cell proliferation | 0 | GO:44249 | Cellular biosynthetic process | 0 |
| GO:0007586 | Digestion | 2.26E-238 | GO:9303 | Rrna transcription | 0 |
| GO:0010884 | Positive regulation of lipid storage | 5.38E-18 | GO:43170 | Macromolecule metabolic process | 0 |
| GO:0042157 | Lipoprotein metabolic process | 3.94E-15 | GO:9059 | Macromolecule biosynthetic process | 0 |
| GO:0034374 | Low-density lipoprotein particle remodeling | 2.49E-13 | GO:16070 | RNA metabolic process | 0 |
| GO:0010744 | Positive regulation of macrophage derived foam cell differentiation | 7.59E-11 | GO:34645 | Cellular macromolecule biosynthetic process | 0 |
| GO:0010886 | Positive regulation of cholesterol storage | 1.15E-10 | GO:8283 | Cell proliferation | 0 |
| GO:0010883 | Regulation of lipid storage | 1.52E-10 | GO:10467 | Gene expression | 0 |
| GO:0097006 | Regulation of plasma lipoprotein particle levels | 1.46E-09 | GO:7586 | Digestion | 8.26E-161 |
| GO:0019915 | Lipid storage | 1.92E-07 | GO:6508 | Proteolysis | 2.26E-47 |
| GO:0010885 | Regulation of cholesterol storage | 3.32E-07 | GO:42632 | Cholesterol homeostasis | 1.53E-18 |
| GO:0071827 | Plasma lipoprotein particle organization | 5.61E-07 | GO:55092 | Sterol homeostasis | 3.20E-18 |
| GO:0071825 | Protein-lipid complex subunit organization | 5.61E-07 | GO:8203 | Cholesterol metabolic process | 4.55E-13 |
| GO:0034369 | Plasma lipoprotein particle remodeling | 7.89E-07 | GO:16125 | Sterol metabolic process | 1.21E-11 |
| GO:0034368 | Protein-lipid complex remodeling | 7.89E-07 | GO:42157 | Lipoprotein metabolic process | 2.48E-11 |
| GO:0034367 | Macromolecular complex remodeling | 7.89E-07 | GO:10884 | Positive regulation of lipid storage | 3.74E-09 |
| GO:0042632 | Cholesterol homeostasis | 1.64E-06 | GO:55088 | Lipid homeostasis | 1.75E-07 |
| GO:0055092 | Sterol homeostasis | 1.64E-06 | GO:34374 | Low-density lipoprotein particle remodeling | 9.13E-07 |
| GO:0006642 | Triglyceride mobilization | 1.90E-06 | GO:97006 | Regulation of plasma lipoprotein particle levels | 2.12E-06 |
| GO:0010878 | Cholesterol storage | 4.59E-06 | GO:10883 | Regulation of lipid storage | 3.27E-06 |
| GO:0010743 | Regulation of macrophage derived foam cell differentiation | 6.75E-06 | GO:10743 | Regulation of macrophage derived foam cell differentiation | 4.25E-05 |
| GO:0042159 | Lipoprotein catabolic process | 2.48E-04 | GO:71827 | Plasma lipoprotein particle organization | 1.58E-04 |
| GO:0090077 | Foam cell differentiation | 6.03E-04 | GO:71825 | Protein-lipid complex subunit organization | 1.58E-04 |
| GO:0010742 | Macrophage derived foam cell differentiation | 6.03E-04 | GO:90077 | Foam cell differentiation | 1.91E-04 |
| GO:0019464 | Glycine decarboxylation via glycine cleavage system | 0.0054 | GO:10742 | Macrophage derived foam cell differentiation | 1.91E-04 |
| GO:0032309 | Icosanoid secretion | 0.0057 | GO:10744 | Positive regulation of macrophage derived foam cell differentiation | 2.09E-04 |
| GO:0006857 | Oligopeptide transport | 0.0061 | GO:10886 | Positive regulation of cholesterol storage | 3.92E-04 |
| GO:0006546 | Glycine catabolic process | 0.0061 | GO:10885 | Regulation of cholesterol storage | 8.13E-04 |
| GO:0061302 | Smooth muscle cell-matrix adhesion | 0.0065 | GO:35814 | Negative regulation of renal sodium excretion | 0.0016 |
| GO:0009071 | Serine family amino acid catabolic process | 0.0068 | GO:71436 | Sodium ion export | 0.0023 |
| GO:2000404 | Regulation of T cell migration | 0.0068 | GO:19915 | Lipid storage | 3.25E-03 |
| GO:0046464 | Acylglycerol catabolic process | 7.04E-03 | GO:51443 | Positive regulation of ubiquitin-protein ligase activity | 0.0033 |
| GO:0046461 | Neutral lipid catabolic process | 7.04E-03 | GO:19370 | Leukotriene biosynthetic process | 0.0045 |
| GO:0016125 | Sterol metabolic process | 7.78E-03 | GO:6030 | Chitin metabolic process | 0.0056 |
| GO:0006956 | Complement activation | 0.0081 | GO:51437 | Positive regulation of ubiquitin-protein ligase activity involved in mitotic cell cycle | 0.0060 |
| GO:0055089 | Fatty acid homeostasis | 0.0081 | GO:51351 | Positive regulation of ligase activity | 0.0060 |
| GO:0015889 | Cobalamin transport | 0.0086 | GO:6637 | Acyl-coa metabolic process | 0.0063 |
