# Supplementary material for: The transcriptome of metamorphosing flatfish
Source: BMC Genomics. 2016 May 27;17:413. doi: 10.1186/s12864-016-2699-x (PMC4884423; doi:10.1186/s12864-016-2699-x)
Supplement: Additional file 8: — Significantly overrepresented Biological Process GO terms identified for the head transcriptome (FDR < 0.05). (DOC 116 kb) [file 12864_2016_2699_MOESM8_ESM.doc]

**Additional file 8: Selected** significantly overrepresented Biological Process GO terms in the head transcriptome (FDR<0.05).

| **Overrepresented in relation to skin** | | | | **Overrepresented in relation to GI tract** | | |
| --- | --- | --- | --- | --- | --- | --- |
| ***GO term ID*** | ***GO term description*** | | ***FDR*** | ***GO term ID*** | ***GO term description*** | ***FDR*** |
| GO:0043170 | Macromolecule metabolic process | | 1.43E-118 | GO:0080090 | Regulation of primary metabolic process | 0 |
| GO:0044260 | Cellular macromolecule metabolic process | | 7.41E-112 | GO:0071842 | Cellular component organization at cellular level | 0 |
| GO:0050794 | Regulation of cellular process | | 2.07E-97 | GO:0071841 | Cellular component organization or biogenesis at cellular level | 0 |
| GO:0019538 | Protein metabolic process | | 2.78E-94 | GO:0071840 | Cellular component organization or biogenesis | 0 |
| GO:0044267 | Cellular protein metabolic process | | 4.16E-90 | GO:0006807 | Nitrogen compound metabolic process | 0 |
| GO:0009059 | Macromolecule biosynthetic process | | 2.04E-82 | GO:0007275 | Multicellular organismal development | 0 |
| GO:0034645 | Cellular macromolecule biosynthetic process | | 3.98E-81 | GO:0019222 | Regulation of metabolic process | 0 |
| GO:0006412 | Translation | | 3.93E-79 | GO:0060255 | Regulation of macromolecule metabolic process | 0 |
| GO:0010467 | Gene expression | | 5.45E-76 | GO:0006725 | Cellular aromatic compound metabolic process | 0 |
| GO:0031323 | Regulation of cellular metabolic process | | 3.47E-75 | GO:0071704 | Organic substance metabolic process | 0 |
| GO:0080090 | Regulation of primary metabolic process | | 2.01E-71 | GO:0065007 | Biological regulation | 0 |
| GO:0007166 | Cell surface receptor signaling pathway | | 2.17E-71 | GO:0007154 | Cell communication | 0 |
| GO:0023052 | Signaling | | 1.94E-68 | GO:0006412 | Translation | 0 |
| GO:0031326 | Regulation of cellular biosynthetic process | | 6.65E-68 | GO:0051716 | Cellular response to stimulus | 0 |
| GO:0019222 | Regulation of metabolic process | | 3.70E-67 | GO:0032502 | Developmental process | 0 |
| GO:0007154 | Cell communication | | 5.28E-67 | GO:0032501 | Multicellular organismal process | 0 |
| GO:0022613 | Ribonucleoprotein complex biogenesis | | 2.17E-66 | GO:0048856 | Anatomical structure development | 0 |
| GO:0009889 | Regulation of biosynthetic process | | 2.26E-66 | GO:0090304 | Nucleic acid metabolic process | 0 |
| GO:0071843 | Cellular component biogenesis at cellular level | | 8.55E-66 | GO:0006139 | Nucleobase-containing compound metabolic process | 0 |
| GO:0008104 | Protein localization | | 1.18E-63 | GO:0048731 | System development | 0 |
| GO:0010556 | Regulation of macromolecule biosynthetic process | | 1.20E-63 | GO:0044267 | Cellular protein metabolic process | 0 |
| GO:0042254 | Ribosome biogenesis | | 2.44E-63 | GO:0044260 | Cellular macromolecule metabolic process | 0 |
| GO:0051716 | Cellular response to stimulus | | 1.26E-62 | GO:0044249 | Cellular biosynthetic process | 0 |
| GO:0007399 | Nervous system development | | 1.11E-61 | GO:0044238 | Primary metabolic process | 0 |
| GO:2000112 | Regulation of cellular macromolecule biosynthetic process | | 1.42E-61 | GO:0050896 | Response to stimulus | 0 |
| GO:0009987 | Cellular process | | 7.34E-56 | GO:0050794 | Regulation of cellular process | 0 |
| GO:0007165 | Signal transduction | | 8.58E-55 | GO:0050789 | Regulation of biological process | 0 |
| GO:0050789 | Regulation of biological process | | 1.65E-54 | GO:0044085 | Cellular component biogenesis | 0 |
| GO:0065007 | Biological regulation | | 4.08E-53 | GO:0051179 | Localization | 0 |
| GO:0048522 | Positive regulation of cellular process | | 1.25E-52 | GO:0009653 | Anatomical structure morphogenesis | 0 |
| GO:0010468 | Regulation of gene expression | | 3.99E-52 | GO:0031323 | Regulation of cellular metabolic process | 0 |
| GO:0060255 | Regulation of macromolecule metabolic process | | 1.38E-51 | GO:0043170 | Macromolecule metabolic process | 0 |
| GO:0045184 | Establishment of protein localization | | 1.15E-50 | GO:0009059 | Macromolecule biosynthetic process | 0 |
| GO:0033036 | Macromolecule localization | | 1.83E-50 | GO:0009058 | Biosynthetic process | 0 |
| GO:0022008 | Neurogenesis | | 4.90E-50 | GO:0016070 | RNA metabolic process | 0 |
| GO:0070727 | Cellular macromolecule localization | | 9.63E-50 | GO:0016043 | Cellular component organization | 0 |
| GO:0048699 | Generation of neurons | | 1.36E-49 | GO:0034645 | Cellular macromolecule biosynthetic process | 0 |
| GO:0050877 | Neurological system process | | 2.01E-49 | GO:0034641 | Cellular nitrogen compound metabolic process | 0 |
| GO:0034613 | Cellular protein localization | | 5.57E-49 | GO:0023052 | Signaling | 0 |
| GO:0015031 | Protein transport | | 4.20E-48 | GO:0046483 | Heterocycle metabolic process | 0 |
| GO:0048666 | Neuron development | | 5.89E-40 | GO:0022008 | Neurogenesis | 6.69E-144 |
| GO:0031175 | Neuron projection development | | 5.03E-36 | GO:0048699 | Generation of neurons | 9.78E-139 |
| GO:0044281 | Small molecule metabolic process | | 6.64E-24 | GO:0048583 | Regulation of response to stimulus | 2.49E-136 |
| GO:0019637 | Organophosphate metabolic process | | 6.55E-18 | GO:0030182 | Neuron differentiation | 3.85E-130 |
| GO:0001501 | Skeletal system development | | 1.57E-17 | GO:0048666 | Neuron development | 1.66E-114 |
| GO:0007417 | Central nervous system development | | 1.38E-11 | GO:0031175 | Neuron projection development | 7.70E-111 |
| GO:0006950 | Response to stress | | 4.78E-10 | GO:0048812 | Neuron projection morphogenesis | 1.56E-97 |
| GO:0009056 | Catabolic process | | 3.13E-08 | GO:0007409 | Axonogenesis | 7.72E-86 |
| GO:0007420 | Brain development | | 5.71E-06 | GO:0007411 | Axon guidance | 2.66E-67 |
| GO:0030900 | Forebrain development | | 0.0001 | GO:0051216 | Cartilage development | 1.67E-54 |
| GO:0048840 | Otolith development | | 0.0010 | GO:0007420 | Brain development | 2.08E-51 |
| GO:0046879 | Hormone secretion | | 0.0013 | GO:0002520 | Immune system development | 1.70E-41 |
| GO:0009914 | Hormone transport | | 0.0014 | GO:0007268 | Synaptic transmission | 2.87E-34 |
| GO:0021578 | Hindbrain maturation | | 0.0023 | GO:0060350 | Endochondral bone morphogenesis | 3.02E-34 |
| GO:0048856 | Anatomical structure development | | 0.0028 | GO:0060537 | Muscle tissue development | 2.33E-32 |
| GO:0042445 | Hormone metabolic process | | 0.0028 | GO:0042246 | Tissue regeneration | 2.34E-32 |
| GO:0008152 | Metabolic process | | 3.97E-03 | GO:0060348 | Bone development | 7.72E-32 |
| GO:0021575 | Hindbrain morphogenesis | | 0.005 | GO:0060349 | Bone morphogenesis | 9.30E-32 |
| GO:0048170 | Positive regulation of long-term neuronal synaptic plasticity | | 0.005 | GO:0030900 | Forebrain development | 1.05E-25 |
| GO:0035284 | Brain segmentation | | 0.005 | GO:0030099 | Myeloid cell differentiation | 1.55E-25 |
| GO:0023041 | Neuronal signal transduction | | 0.008 | GO:0010975 | Regulation of neuron projection development | 3.59E-24 |
| GO:0021983 | Pituitary gland development | | 0.008 | GO:0001649 | Osteoblast differentiation | 5.68E-23 |
| GO:0032870 | Cellular response to hormone stimulus | | 0.012 | GO:0060828 | Regulation of canonical Wnt receptor signaling pathway | 8.48E-23 |
| GO:0070167 | Regulation of biomineral tissue development | | 0.013 | GO:0060322 | Head development | 2.45E-19 |
| GO:0045161 | Neuronal ion channel clustering | | 0.018 | GO:0060351 | Cartilage development involved in endochondral bone morphogenesis | 1.13E-17 |
| GO:0021846 | Cell proliferation in forebrain | | 0.020 | GO:0060323 | Head morphogenesis | 2.72E-17 |
| GO:0021533 | Cell differentiation in hindbrain | | 0.029 | GO:0001764 | Neuron migration | 9.86E-10 |
| GO:0097150 | Neuronal stem cell maintenance | | 0.029 | GO:0021537 | Telencephalon development | 2.57E-07 |
| GO:0030072 | Peptide hormone secretion | | 0.030 | GO:0006836 | Neurotransmitter transport | 2.86E-05 |
| GO:0042403 | Thyroid hormone metabolic process | | 0.044 | GO:0016358 | Dendrite development | 6.01E-05 |
| GO:0010033 | Response to organic substance | | 0.046 | GO:0009755 | Hormone-mediated signaling pathway | 0.0070 |
|  | | | | GO:0032400 | Melanosome localization | 0,0082 |
|  |  |  | | GO:0021697 | Cerebellar cortex formation | 0.0086 |
|  |  |  | | GO:0030878 | Thyroid gland development | 0.012 |
|  |  |  | | GO:0021510 | Spinal cord development | 2,60E-02 |
|  |  |  | | GO:0048545 | Response to steroid hormone stimulus | 2.63E-12 |
|  |  |  | | GO:0009725 | Response to hormone stimulus | 5.30E-10 |
|  |  |  | | GO:0009914 | Hormone transport | 7.58E-05 |
|  |  |  | | GO:0043434 | Response to peptide hormone stimulus | 7,35E-03 |
