# Supplementary material for: The transcriptome of metamorphosing flatfish
Source: BMC Genomics. 2016 May 27;17:413. doi: 10.1186/s12864-016-2699-x (PMC4884423; doi:10.1186/s12864-016-2699-x)
Supplement: Additional file 9: — List of the most representative metabolic pathways in the skin, GI-tract and head transcriptomes using KEGG analysis. (DOCX 16 kb) [file 12864_2016_2699_MOESM9_ESM.docx]

**Additional file 9.** List of the most representative metabolic pathways in the head, skin and GI-tract transcriptomes using KEGG analysis.

|  | **Number of enzyme codes (EC)** | | |  |
| --- | --- | --- | --- | --- |
| **KEGG pathway** | **Skin** | **GI-tract** | **Head** |  |
| Purine metabolism | 56 | 51 | 58 |  |
| Amino sugar and nucleotide sugar metabolism | 31 | 31 | 35 |  |
| Pyrimidine metabolism | 36 | 33 | 34 |  |
| Arginine and proline metabolism | 35 | 30 | 32 |  |
| Glycerophospholipid metabolism | 30 | 26* | 29 |  |
| Glycine, serine and threonine metabolism | 32 | 29 | 29 |  |
| Glycolysis / Gluconeogenesis | 28 | 27 | 26 |  |
| Alanine, aspartate and glutamate metabolism | 23 | 18 | 25 |  |
| Cysteine and methionine metabolism | 24 | 20 | 25 |  |
| Inositol phosphate metabolism | 22 | 20* | 24 |  |
| Pyruvate metabolism | 24 | 23 | 24 |  |
| Aminoacyl-tRNA biosynthesis | 22 | 22 | 23 |  |
| Valine, leucine and isoleucine degradation | 22 | 20 | 22 |  |
| Phosphatidylinositol signaling system | 20 | 18* | 20 |  |
| Sphingolipid metabolism | 19 | 17* | 20 |  |
| Citrate cycle (TCA cycle) | 19 | 18 | 19 |  |
| Glutathione metabolism | 19 | 18 | 19 |  |
| Starch and sucrose metabolism | 23 | 24 | 19★ |  |
| Drug metabolism - other enzymes | 18 | 18 | 18 |  |
| Glycosaminoglycan biosynthesis - chondroitin sulfate | 10 | 4🟂 | 11 |  |
| Glycosaminoglycan biosynthesis - heparan sulfate | 8 | 4🟂 | 10 |  |
| Steroid biosynthesis | 13 | 12 | 13 |  |
| Steroid degradation | 3 | 3 | 3 |  |
| Steroid hormone biosynthesis | 18 | 15* | 18 |  |

* - indicates lipid related metabolic pathways substantially modified between tissues

🟂 - indicates metabolic pathways involved in chondrogenic matrix generation substantially different between tissues

★ - indicates starch and sucrose metabolism is substantially different between tissues
