# Supplementary material for: The transcriptome of metamorphosing flatfish
Source: BMC Genomics. 2016 May 27;17:413. doi: 10.1186/s12864-016-2699-x (PMC4884423; doi:10.1186/s12864-016-2699-x)
Supplement: Additional file 10: — The list of the top most significantly up-regulated genes between premetamorphic stage 5 and the Juvenile stage determined using a linear model in Bayseq with a Benjamin-Hochberg adjustment for multiple testing analysis with a cut-off set at 0.05 (FDR < 0.05). Contig name, Gene name, Accession number (no.), Organism and E-value are shown for each gene. (DOCX 75 kb) [file 12864_2016_2699_MOESM10_ESM.docx]

**Additional file 10a:** List of top most significantly upregulated genes between premetamorphic stage 5 and juvenile stage as determined by a linear model in Bayseq with a Benjamin-Hochberg adjustment for multiple testing analysis with a cut-off set at 0.05 (FDR<0.05). Contig name, Gene name, Accession number (no.), Organism and E-value are shown for each gene.

| **Upregulated genes in premetamorphic stage 5 compared to juvenile** | |  |  |  |
| --- | --- | --- | --- | --- |
| ***Contig ID*** | ***Gene name*** | ***Accession no.*** | ***Organism*** | ***E-value*** |
| Contig07799 | Putative uncharacterized protein ART2-like | XP_004616260.1 | *Sorex araneus* | 3E-27 |
| Contig1693 | Ribosomal protein S23 | AEJ84339.1 | *Capra hircus* | 8E-30 |
| Contig26083 | Hypothetical protein BOS_1871 | DAA32622.1 | *Bos taurus* | 3E-30 |
| Contig26869 | Hypothetical protein LOC100559304 | XP_003230372.1 | *Anolis carolinensis* | 6E-21 |
| Contig04456 | TOM1-like protein 2 | CBN81216.1 | *Dicentrarchus labrax* | 0 |
| Contig14322 | CD59 glycoprotein-like | XP_003460314.1 | *Oreochromis niloticus* | 2E-43 |
| Contig1667 | Vinculin | CBN81495.1 | *Dicentrarchus labrax* | 0 |
| Contig20143 | Collagen alpha-1(I) chain-like | XP_003460741.1 | *Cavia porcellus* | 2E-31 |
| Contig1362 | Rrna promoter binding protein-like | XP_002724111.1 | *Oryctolagus cuniculus* | 9E-27 |
| Contig16982 | Ryanodine receptor 1-like | XP_004560674.1 | *Maylandia zebra* | 3E-73 |
| Contig17221 | Latrophilin-2 | XP_002663750.2 | *Danio rerio* | 2E-67 |
| Contig18157 | Neutral ceramidase-like | XP_003961257.1 | *Takifugu rubripes* | 1E-82 |
| Contig13137 | Dedicator of cytokinesis protein 7 isoform X6 | XP_005468811.1 | *Oreochromis niloticus* | 2E-157 |
| Contig1230 | Unnamed protein product | BAE33391.1 | *Mus musculus* | 5E-26 |
| Contig18278 | Unnamed protein product | BAE43022.1 | *Mus musculus* | 6E-40 |
| Contig25150 | Translation initiation factor IF-2-like | XP_004450492.1 | *Dasypus novemcinctus* | 4E-20 |
| Contig1002 | Keratin 1 | ABC88386.1 | *Hippoglossus hippoglossus* | 2E-43 |
| **Upregulated genes in juvenile compared to premetamorphic stage 5** | |  |  |  |
| Contig1352 | Actin alpha skeletal muscle | ACM41845.1 | *Epinephelus coioides* | 1E-114 |
| Contig1091 | AF500273_1 fast skeletal muscle alpha-actin | AAM21702.2 | *Gadus morhua* | 5E-72 |
| Contig1395 | Tetraspanin-12-like | XP_003448856.1 | *Oreochromis niloticus* | 2E-93 |
| Contig355 | Protein-glutamine gamma-glutamyltransferase 5-like | XP_003448488.1 | *Oreochromis niloticus* | 0 |
| Contig1315 | Sarcoplasmic/endoplasmic reticulum calcium atpase 1 | P70083.2 | *Makaira nigricans* | 0 |
| Contig1500 | Ribosomal protein, large, P0 | NP_001080134.1 | *Xenopus laevis* | 6E-50 |
| Contig1214 | Nucleoside diphosphate kinase A-like | XP_003442598.1 | *Oreochromis niloticus* | 4E-75 |
| Contig796 | Myosin light chain 2, isoform B | CAD32552.1 | *Hippoglossus hippoglossus* | 2E-86 |
| Contig1162 | Cytochrome oxidase subunit I | CAO79645.1 | *Hippoglossus hippoglossus* | 1E-112 |
| Contig20504 | Type-4 ice-structuring protein | Q8JI37.1 | *Paralichthys olivaceus* | 3E-40 |
| Contig995 | Myosin heavy chain | ADG29145.1 | *Epinephelus coioides* | 1E-136 |
| Contig595 | Transcription factor E2F4-like | XP_003447142.1 | *Oreochromis niloticus* | 1E-106 |
| Contig1137 | Heat shock protein 70 | NP_001098385.1 | *Oryzias latipes* | 0 |
| Contig28671 | Trypsinogen Flou2 | AAY23358.1 | *Hippoglossus hippoglossus* | 5E-43 |
| Contig18804 | Apolipoprotein E | ACF21982.1 | *Oplegnathus fasciatus* | 8E-87 |
| Contig1000 | Parvalbumin | BAF98925.1 | *Paralichthys olivaceus* | 1E-42 |
| Contig15768 | Ribosomal protein L18a | BAF98666.1 | *Solea senegalensis* | 1E-98 |
| Contig1625 | Putative 14 kda apolipoprotein | CAH57705.1 | *Platichthys flesus* | 3E-58 |
| Contig04453 | Trypsinogen 2 precursor | AAC32752.1 | *Pseudopleuronectes americanus* | 9E-32 |
| Contig25330 | Sarcoendoplasmic reticulum calcium atpase | ABG90496.1 | *Silurus lanzhouensis* | 2E-28 |
| Contig411 | Elongation factor 1 alpha isoform 2 | BAF64485.1 | *Solea senegalensis* | 0 |
| Contig29 | Alpha-actinin-3-like isoform 1 | XP_003447595.1 | *Oreochromis niloticus* | 0 |
| Contig1056 | Sarcoplasmic/endoplasmic reticulum calcium atpase 1-like isoform X1 | XP_003454037.1 | *Oreochromis niloticus* | 0 |
| Contig24482 | Actin, gamma-enteric smooth muscle-like isoform 1 | XP_003201904.1 | *Meleagris gallopavo* | 2E-26 |
| Contig1671 | Apolipoprotein AI precursor | CAH59609.1 | *Platichthys flesus* | 1E-102 |
| Contig1001 | 60S ribosomal protein L21-like | XP_005797910.1 | *Xiphophorus maculatus* | 3E-104 |
| Contig539 | Unnamed protein product | CAG02349.1 | *Tetraodon nigroviridis* | 1E-126 |
| Contig05820 | Fish-egg lectin | BAL61198.1 | *Oplegnathus fasciatus* | 2E-83 |
| Contig25352 | Alpha-cardiac actin | AAA37165.1 | *Mus musculus* | 1E-22 |
| Contig25161 | Alpha-actin | AAX18244.1 | *Acipenser transmontanus* | 1E-48 |
| Contig13800 | Myosin light chain 1, skeletal muscle isoform-like isoform X1 | XP_003445346.1 | *Oreochromis niloticus* | 8E-77 |
| Contig1172 | Ribosomal protein L6 | AEH76599.1 | *Epinephelus bruneus* | 2E-56 |
| Contig72 | Eukaryotic initiation factor 4A-I isoform X1 | XP_003458967.1 | *Oreochromis niloticus* | 0 |
| Contig26602 | Parvalbumin | AAY27426.1 | *Paralichthys olivaceus* | 7E-17 |
| Contig06809 | Heat shock protein 90 beta | AAP20179.1 | *Pagrus major* | 1E-117 |
| Contig1356 | Beta actin | ACZ63697.1 | *Hippoglossus hippoglossus* | 4E-74 |
| Contig615 | Carboxypeptidase B | BAC53789.1 | *Paralichthys olivaceus* | 1E-146 |
| Contig20859 | 60S ribosomal protein L3-like | XP_004071207.1 | *Oryzias latipes* | 2E-123 |
| Contig772 | AP-3 complex subunit mu-2 | XP_003448631.1 | *Oreochromis niloticus* | 0 |
| Contig21912 | Chymotrypsinogen 1 | BAL14136.1 | *Thunnus orientalis* | 5E-87 |
| Contig1030 | Elongation factor 1 alpha | ACE82251.1 | *Hippoglossus hippoglossus* | 1E-151 |
| Contig1360 | Sarcoplasmic/endoplasmic reticulum calcium atpase 1-like | XP_003458779.1 | *Oreochromis niloticus* | 1E-18 |
| Contig1385 | 60S ribosomal protein | AEB31272.1 | *Epinephelus bruneus* | 2E-83 |
| Contig1592 | 40S ribosomal protein S26 | ABU98965.1 | *Hippoglossus hippoglossus* | 3E-45 |
| Contig834 | Keratin, type I cytoskeletal 18-like | XP_003449152.1 | *Oreochromis niloticus* | 5E-27 |
| Contig25154 | Chymotrypsinogen 2 | BAL14137.1 | *Thunnus orientalis* | 2E-43 |
| Contig979 | 40S ribosomal protein Sa | AAP20147.1 | *Pagrus major* | 5E-74 |
| Contig399 | Rho guanine nucleotide exchange factor 6-like | XP_003445205.1 | *Oreochromis niloticus* | 0 |
| Contig1572 | Receptor for activated protein kinase C | AAT35603.1 | *Paralichthys olivaceus* | 1E-74 |
| Contig17281 | 60S ribosomal protein L19 | ACO09605.1 | *Osmerus mordax* | 6E-65 |
| Contig520 | Collagen alpha-1(I) chain-like | XP_003453458.1 | *Oreochromis niloticus* | 9E-71 |

**Additional file 10b:** List of top most significantly upregulated genes between metamorphic proclimax stage 8 and premetamorphic stage 5 as determined by a linear model in Bayseq with a Benjamin-Hochberg adjustment for multiple testing analysis with a cut-off set at 0.05 (FDR<0.05). Contig name, Gene name, Accession number (no.), Organism and E-value are shown for each gene.

| **Upregulated genes in metamorphic proclimax stage 8 compared to premetamorphic stage 5** | |  |  |  |
| --- | --- | --- | --- | --- |
| ***Contig ID*** | ***Gene name*** | ***Accession no.*** | ***Organism*** | ***E-value*** |
| Contig04550 | Lactate dehydrogenase-A | AAP44524.1 | *Chromis caudalis* | 1E-166 |
| Contig347 | Carboxypeptidase A2 | BAC53787.1 | *Paralichthys olivaceus* | 0 |
| Contig405 | Secretory carrier-associated membrane protein 3-like | XP_003969342.1 | *Takifugu rubripes* | 3E-83 |
| Contig28608 | Beta-enolase | ADG29136.1 | *Epinephelus coioides* | 1E-27 |
| Contig11326 | Phospholipase A2 | BAA23737.1 | *Pagrus major* | 1E-69 |
| Contig179 | Lumican-like | XP_003444990.1 | *Oreochromis niloticus* | 1E-155 |
| Contig10151 | Chymotrypsinogen | ADG29171.1 | *Epinephelus coioides* | 3E-82 |
| Contig1249 | Glyceraldehyde-3-phosphate dehydrogenase | ABY19518.1 | *Paralichthys olivaceus* | 2E-19 |
| Contig28338 | Parvalbumin I | 1206380A | *Electrophorus sp.* | 7E-12 |
| Contig06996 | Inositol monophosphatase 1-like isoform X1 | XP_003439317.1 | *Oreochromis niloticus* | 1E-135 |
| Contig16531 | Gelsolin-like | XP_003437831.1 | *Oreochromis niloticus* | 5E-74 |
| Contig1276 | Trypsinogen | AEM91638.1 | *Channa argus* | 5E-48 |
| Contig04519 | Myc box-dependent-interacting protein 1-like | XP_003446611.1 | *Oreochromis niloticus* | 3E-36 |
| Contig1607 | Chymotrypsinogen 1 | BAA82365.1 | *Paralichthys olivaceus* | 6E-22 |
| Contig22951 | Apolipoprotein B-100-like | XP_003446425.1 | *Oreochromis niloticus* | 9E-52 |
| Contig06549 | Keratin, type I cytoskeletal 18 | CBN80920.1 | *Dicentrarchus labrax* | 1E-154 |
| Contig03409 | Hypothetical protein LOC100330916 | XP_002660725.2 | *Danio rerio* | 1E-135 |
| Contig1422 | Fructose-bisphosphate aldolase A | NP_001167391.1 | *Salmo salar* | 2E-18 |
| Contig1087 | Phosphoglycerate mutase 2-like | XP_003444406.1 | *Oreochromis niloticus* | 1E-66 |
| Contig708 | Adenine nucleotide translocator s254 | NP_001037840.1 | *Takifugu rubripes* | 1E-68 |
| Contig12118 | Elastase-like serine protease | ACG50688.1 | *Paralichthys olivaceus* | 1E-141 |
| Contig553 | 15-hydroxyprostaglandin dehydrogenase [NAD(+)]-like | XP_003451463.1 | *Oreochromis niloticus* | 3E-73 |
| Contig13429 | Latisemin-like | XP_003971902.1 | *Takifugu rubripes* | 5E-132 |
| Contig03682 | Heparin cofactor II | ACC86113.1 | *Paralichthys olivaceus* | 0 |
| Contig12730 | Cytochrome c oxidase subunit VIIa-related protein, mitochondrial | CBN81583.1 | *Dicentrarchus labrax* | 5E-49 |
| Contig00337 | NAD(P) transhydrogenase, mitochondrial-like isoform X1 | XP_003456827.1 | *Oreochromis niloticus* | 0 |
| Contig02752 | Cytosolic phospholipase A2 zeta-like | XP_003446488.1 | *Oreochromis niloticus* | 0 |
| Contig06935 | Inosine-uridine preferring nucleoside hydrolase-like | XP_003977955.1 | *Takifugu rubripes* | 0 |
| Contig03255 | Protein disulfide-isomerase-like | XP_003456032.1 | *Oreochromis niloticus* | 0 |
| Contig08871 | Cytolysin Src-1-like | XP_003442214.1 | *Oreochromis niloticus* | 1E-74 |
| Contig05635 | Alpha-2-HS-glycoprotein-like | XP_004078952.1 | *Oryzias latipes* | 2E-111 |
| Contig14518 | Uncharacterized protein LOC100709362 | XP_003454247.1 | *Oreochromis niloticus* | 3E-27 |
| Contig15586 | Diazepam-binding inhibitor | AEK25827.1 | *Micropterus salmoides* | 2E-31 |
| Contig149 | GTP-binding protein PTD004 | ACO09273.1 | *Osmerus mordax* | 0 |
| **Upregulated genes in premetamorphic stage 5 compared to metamorphic proclimax stage 8** | |  |  |  |
| Contig05607 | Type I keratin isoform 2 | BAF56914.1 | *Solea senegalensis* | 1E-145 |
| Contig06282 | Keratin, type I cytoskeletal 13-like | XP_003453825.1 | *Oreochromis niloticus* | 1E-144 |

**Additional file 10c:** List of top most significantly upregulated genes between metamorphic climax stage 9A and premetamorphic stage 5 as determined by a linear model in Bayseq with a Benjamin-Hochberg adjustment for multiple testing analysis with a cut-off set at 0.05 (FDR<0.05). Contig name, Gene name, Accession number (no.), Organism and E-value are shown for each gene.

| **Upregulated genes in metamorphic climax stage 9A compared to premetamorphic stage 5** | |  |  |  |
| --- | --- | --- | --- | --- |
| ***Contig ID*** | ***Gene name*** | ***Accession no.*** | ***Organism*** | ***E-value*** |
| Contig10151 | Chymotrypsinogen | ADG29171.1 | *Epinephelus coioides* | 3E-82 |
| Contig06996 | Inositol monophosphatase 1-like isoform X1 | XP_003439317.1 | *Oreochromis niloticus* | 1E-135 |
| Contig1276 | Trypsinogen | AEM91638.1 | *Channa argus* | 5E-48 |
| Contig1422 | Fructose-bisphosphate aldolase A | NP_001167391.1 | *Salmo salar* | 2E-18 |
| Contig12118 | Elastase-like serine protease | ACG50688.1 | *Paralichthys olivaceus* | 1E-141 |
| Contig1087 | Phosphoglycerate mutase 2-like | XP_003444406.1 | *Oreochromis niloticus* | 1E-66 |
| Contig03409 | Hypothetical protein LOC100330916 | XP_002660725.2 | *Danio rerio* | 1E-135 |
| Contig708 | Adenine nucleotide translocator s254 | NP_001037840.1 | *Takifugu rubripes* | 1E-68 |
| Contig28671 | Trypsinogen Flou2 | AAY23358.1 | *Hippoglossus hippoglossus* | 5E-43 |
| Contig553 | 15-hydroxyprostaglandin dehydrogenase [NAD(+)]-like | XP_003451463.1 | *Oreochromis niloticus* | 3E-73 |
| Contig06935 | Inosine-uridine preferring nucleoside hydrolase-like | XP_003977955.1 | *Takifugu rubripes* | 0 |
| Contig05820 | Fish-egg lectin | BAL61198.1 | *Oplegnathus fasciatus* | 2E-83 |
| Contig00337 | NAD(P) transhydrogenase, mitochondrial-like isoform X1 | XP_003456827.1 | *Oreochromis niloticus* | 0 |
| Contig02752 | Cytosolic phospholipase A2 zeta-like | XP_003446488.1 | *Oreochromis niloticus* | 0 |
| Contig03255 | Protein disulfide-isomerase-like | XP_003456032.1 | *Oreochromis niloticus* | 0 |
| Contig05635 | Alpha-2-HS-glycoprotein-like | XP_004078952.1 | *Oryzias latipes* | 2E-111 |
| Contig08871 | Cytolysin Src-1-like | XP_003442214.1 | *Oreochromis niloticus* | 1E-74 |
| **Upregulated genes in premetamorphic stage 5 compared to metamorphic climax stage 9A** | |  |  |  |
| Contig05607 | Type I keratin isoform 2 | BAF56914.1 | *Solea senegalensis* | 1E-145 |
| Contig06282 | Keratin, type I cytoskeletal 13-like | XP_003453825.1 | *Oreochromis niloticus* | 1E-144 |
| Contig01282 | Uncharacterized protein LOC100691699 isoform X2 | XP_005449125.1 | *Oreochromis niloticus* | 0 |
| Contig00195 | Fibrocystin-L | XP_003447322.1 | *Oreochromis niloticus* | 0 |
| Contig923 | Alpha-amylase | ABJ97444.1 | *Xiphister atropurpureus* | 1E-148 |
| Contig03232 | Macrophage mannose receptor 1-like | XP_003437865.1 | *Oreochromis niloticus* | 3E-77 |
| Contig09466 | Zinc finger protein 628-like isoform X5 | XP_005455430.1 | *Oreochromis niloticus* | 8E-112 |
| Contig17770 | Ankyrin repeat and KH domain-containing protein 1 isoform X1 | XP_003445913.1 | *Oreochromis niloticus* | 8E-76 |
| Contig08520 | UDP-glucose:glycoprotein glucosyltransferase 2 | XP_003447406.1 | *Oreochromis niloticus* | 0 |
| Contig06464 | SH2 domain-containing protein 5-like | XP_004069380.1 | *Oryzias latipes* | 0 |
| Contig12963 | Hypothetical protein LOC100696747 | XP_003455026.1 | *Oreochromis niloticus* | 6E-47 |
| Contig03281 | Synaptotagmin-11-like | XP_003451315.1 | *Oreochromis niloticus* | 0 |
| Contig01305 | Chromobox protein homolog 2-like | XP_003442539.1 | *Oreochromis niloticus* | 1E-113 |
| lchead_c16028 | Contactin-associated protein-like 2 | XP_003456781.1 | *Oreochromis niloticus* | 1E-149 |
| Contig05135 | Tenascin-X-like | XP_003459083.1 | *Oreochromis niloticus* | 0 |
| Contig09575 | Myotubularin-related protein 7-like | XP_003455820.1 | *Oreochromis niloticus* | 1E-115 |
| Contig03314 | Cyclin-T2-like | XP_003443331.1 | *Oreochromis niloticus* | 9E-17 |
| Contig06850 | Cryptochrome-1-like | XP_003452063.1 | *Oreochromis niloticus* | 0 |
| Contig17960 | Low-density lipoprotein receptor-related protein 1-like | XP_003963209.1 | *Takifugu rubripes* | 2E-107 |
| Contig07286 | Phosphorylated CTD-interacting factor 1 | XP_003457750.1 | *Oreochromis niloticus* | 1E-106 |
| Contig02198 | Semaphorin-7A-like | XP_003450354.1 | *Oreochromis niloticus* | 1E-127 |
| Contig02216 | Uncharacterized protein LOC100693968 isoform X1 | XP_003446915.1 | *Oreochromis niloticus* | 0 |
| Contig06214 | Neural cell adhesion molecule L1-like isoform X1 | XP_003448281.1 | *Oreochromis niloticus* | 1E-174 |
| Contig07338 | Protein phosphatase Slingshot homolog 2-like isoform X2 | XP_005462386.1 | *Oreochromis niloticus* | 9E-116 |
| Contig132 | Glutamate decarboxylase 2-like | XP_003439331.1 | *Oreochromis niloticus* | 0 |
| Contig05591 | RNA-binding protein 10-like | XP_003448331.1 | *Oreochromis niloticus* | 1E-173 |
| Contig13972 | Hypothetical protein LOC100695447 | XP_003459280.1 | *Oreochromis niloticus* | 5E-66 |
| Contig07598 | Phosphoenolpyruvate carboxykinase | AAX21768.2 | *Acanthopagrus schlegelii* | 0 |
| Contig02885 | Microtubule-associated protein 1B-like | XP_005454597.1 | *Oreochromis niloticus* | 0 |
| Contig1744 | Cap-specific mrna (nucleoside-2'-O-)-methyltransferase 1-like | XP_003449946.1 | *Oreochromis niloticus* | 1E-169 |
| Contig08589 | Oxysterol-binding protein-related protein 8 isoform X1 | XP_003449817.1 | *Oreochromis niloticus* | 1E-166 |
| Contig03144 | Neuroplastin-like isoform X2 | XP_005450939.1 | *Oreochromis niloticus* | 1E-130 |
| lchead_c7007 | Uncharacterized protein c20orf194-like | XP_003447892.1 | *Oreochromis niloticus* | 4E-57 |
| Contig05034 | Sentrin-specific protease 6-like isoform X3 | XP_005452489.1 | *Oreochromis niloticus* | 0 |
| Contig07800 | Phosphatidylinositol 4-kinase type 2-beta-like | XP_003448041.1 | *Oreochromis niloticus* | 0 |
| Contig09966 | Oxysterol-binding protein-related protein 1-like | XP_003977651.1 | *Takifugu rubripes* | 0 |
| Contig23771 | Laminin subunit beta-1-like | XP_003448148.1 | *Oreochromis niloticus* | 4E-69 |
| Contig23056 | Pleckstrin homology-like domain family B member 2 | XP_003446815.1 | *Oreochromis niloticus* | 2E-40 |
| Contig10466 | NEDD8-activating enzyme E1 regulatory subunit-like isoformx1 | XP_003445754.1 | *Oreochromis niloticus* | 1E-117 |
| Contig17157 | Transmembrane protein 134 | ACQ58040.1 | *Anoplopoma fimbria* | 4E-86 |
| Contig16346 | Transformation/transcription domain-associated protein | XP_001919276.3 | *Danio rerio* | 3E-83 |
| Contig07774 | Protein FAM91A1-like | XP_003444196.1 | *Oreochromis niloticus* | 2E-97 |
| Contig17788 | Nuclear receptor ROR-beta-like | XP_003451344.1 | *Oreochromis niloticus* | 7E-13 |
| Contig21166 | RNA-binding protein Musashi homolog 1-like isoformx3 | XP_003441610.1 | *Oreochromis niloticus* | 4E-82 |
| Contig09528 | CUGBP Elav-like family member 3-like isoform 1 | XP_003450950.1 | *Oreochromis niloticus* | 1E-121 |
| Contig15911 | Zinc finger protein ubi-d4-like isoform X1 | XP_003444769.1 | *Oreochromis niloticus* | 6E-75 |
| Contig08795 | Inositol 1,4,5-trisphosphate receptor type 3-like | XP_003220443.1 | *Anolis carolinensis* | 1E-170 |
| Contig23670 | C2 domain-containing protein 5-like isoform X1 | XP_003440632.2 | *Oreochromis niloticus* | 2E-79 |
| Contig31354 | Fmvia | AAD52005.1 | *Morone saxatilis* | 2E-26 |
| Contig13284 | CAD protein-like | XP_003456748.1 | *Oreochromis niloticus* | 1E-146 |
| Contig14221 | Teneurin-4-like isoform 1 | XP_003450204.1 | *Oreochromis niloticus* | 1E-146 |
| Contig05301 | Fascin-like | XP_003443269.1 | *Oreochromis niloticus* | 0 |
| lchead_c76077 | Rho guanine nucleotide exchange factor 11-like | XP_003458910.1 | *Oreochromis niloticus* | 1E-57 |
| Contig18720 | ADP-ribosylation factor-like protein 3-like | XP_003441784.1 | *Oreochromis niloticus* | 3E-94 |
| Contig09378 | Neuronal migration protein doublecortin-like isoform 1 | XP_003446904.1 | *Oreochromis niloticus* | 1E-171 |
| Contig17353 | Ras-related protein Rab-40C-like isoform 1 | XP_003453881.1 | *Oreochromis niloticus* | 4E-40 |
| lchead_c76634 | Protein bassoon-like | XP_003973339.1 | *Takifugu rubripes* | 5E-49 |
| Contig09653 | Vascular endothelial growth factor receptor kdr-like | XP_003445218.1 | *Oreochromis niloticus* | 4E-58 |
| Contig18146 | Cytoplasmic phosphatidylinositol transfer protein 1-like isoform X1 | XP_003458369.1 | *Oreochromis niloticus* | 1E-117 |

**Additional file 10d:** List of top most significantly upregulated genes between metamorphic climax stage 9B and premetamorphic stage 5 as determined by a linear model in Bayseq with a Benjamin-Hochberg adjustment for multiple testing analysis with a cut-off set at 0.05 (FDR<0.05). Contig name, Gene name, Accession number (no.), Organism and E-value are shown for each gene.

| **Up regulated in metamorphic climax stage 9B compared to premetamorphic stage 5** | |  |  |  |
| --- | --- | --- | --- | --- |
| ***Contig ID*** | ***Gene name*** | ***Accession no.*** | ***Organism*** | ***E-value*** |
| Contig06286 | Serpin A3-5-like | XP_003445650.1 | *Oreochromis niloticus* | 1E-134 |
| Contig03409 | Hypothetical protein LOC100330916 | XP_002660725.2 | *Danio rerio* | 1E-135 |
| Contig12118 | Elastase-like serine protease | ACG50688.1 | *Paralichthys olivaceus* | 1E-141 |
| Contig1422 | Fructose-bisphosphate aldolase A | NP_001167391.1 | *Salmo salar* | 2E-18 |
| Contig708 | Adenine nucleotide translocator s254 | NP_001037840.1 | *Takifugu rubripes* | 1E-68 |
| Contig05820 | Fish-egg lectin | BAL61198.1 | *Oplegnathus fasciatus* | 2E-83 |
| Contig05635 | Alpha-2-HS-glycoprotein-like | XP_004078952.1 | *Oryzias latipes* | 2E-111 |
| Contig02752 | Cytosolic phospholipase A2 zeta-like | XP_003446488.1 | *Oreochromis niloticus* | 0 |
| Contig1284 | Keratin, type I cytoskeletal 13-like | XP_003442483.1 | *Oreochromis niloticus* | 1E-29 |
| Contig00708 | Von Willebrand factor A domain-containing protein 7-like isoform X1 | XP_003448088.2 | *Oreochromis niloticus* | 0 |
| **Up regulated in premetamorphic stage 5 compared to metamorphic climax stage 9B** | |  |  |  |
| Contig05607 | Type I keratin isoform 2 | BAF56914.1 | *Solea senegalensis* | 1E-145 |
| Contig494 | High choriolytic enzyme 1-like | XP_003456934.1 | *Oreochromis niloticus* | 4E-87 |

**Additional file 10e:** List of top most significantly upregulated genes between metamorphic climax stage 9C and premetamorphic stage 5 as determined by a linear model in Bayseq with a Benjamin-Hochberg adjustment for multiple testing analysis with a cut-off set at 0.05 (FDR<0.05). Contig name, Gene name, Accession number (no.), Organism and E-value are shown for each gene.

| **Up regulated in metamorphic climax stage 9C compared to premetamorphic stage 5** | |  |  |  |
| --- | --- | --- | --- | --- |
| ***Contig ID*** | ***Gene name*** | ***Accession no.*** | ***Organism*** | ***E-value*** |
| Contig10225 | Apolipoprotein A-IV4 precursor | NP_001027893.1 | *Takifugu rubripes* | 1E-109 |
| Contig179 | Lumican-like | XP_003444990.1 | *Oreochromis niloticus* | 1E-155 |
| Contig28338 | Parvalbumin I | 1206380A | *Electrophorus sp.* | 7E-12 |
| Contig28441 | Putative fast skeletal muscle troponin | AAP82940.1 | *Paralichthys olivaceus* | 4E-15 |
| Contig00901 | Immunoglobulin-like and fibronectin type III domain-containing protein 1-like | XP_003438838.1 | *Oreochromis niloticus* | 0 |
| Contig16531 | Gelsolin-like | XP_003437831.1 | *Oreochromis niloticus* | 5E-74 |
| Contig06996 | Inositol monophosphatase 1-like isoform X1 | XP_003439317.1 | *Oreochromis niloticus* | 1E-135 |
| Contig00664 | Calpain-11 | ACY78223.1 | *Hippoglossus hippoglossus* | 0 |
| Contig06286 | Serine protease inhibitor A3K-like | XP_003445650.1 | *Oreochromis niloticus* | 3E-164 |
| Contig08272 | Beta-2-glycoprotein 1 | ADX97142.1 | *Perca flavescens* | 1E-174 |
| Contig27668 | Apolipoprotein C-I-like | XP_004550997.1 | *Maylandia zebra* | 3E-14 |
| Contig00663 | Fibronectin 1 | CAQ13985.1 | *Danio rerio* | 0 |
| Contig708 | Adenine nucleotide translocator s254 | NP_001037840.1 | *Takifugu rubripes* | 1E-68 |
| Contig12118 | Elastase-like serine protease | ACG50688.1 | *Paralichthys olivaceus* | 1E-141 |
| Contig01049 | Sushi domain-containing protein 2-like | XP_003453945.1 | *Oreochromis niloticus* | 0 |
| Contig03409 | Hypothetical protein LOC100330916 | XP_002660725.2 | *Danio rerio* | 1E-135 |
| Contig00274 | Desmocollin-2-like | XP_003437940.1 | *Oreochromis niloticus* | 0 |
| Contig28671 | Trypsinogen Flou2 | AAY23358.1 | *Hippoglossus hippoglossus* | 5E-43 |
| Contig553 | 15-hydroxyprostaglandin dehydrogenase [NAD(+)]-like | XP_003451463.1 | *Oreochromis niloticus* | 3E-73 |
| Contig03291 | Hyaluronan-binding protein 2, partial | CBN81454.1 | *Dicentrarchus labrax* | 0 |
| Contig03682 | Heparin cofactor II | ACC86113.1 | *Paralichthys olivaceus* | 0 |
| Contig06935 | Inosine-uridine preferring nucleoside hydrolase-like | XP_003977955.1 | *Takifugu rubripes* | 0 |
| Contig00037 | Alpha-tectorin | CBN81411.1 | *Dicentrarchus labrax* | 0 |
| Contig00708 | Von Willebrand factor A domain-containing protein 7-like isoform X1 | XP_003448088.2 | *Oreochromis niloticus* | 0 |
| Contig05820 | Fish-egg lectin | BAL61198.1 | *Oplegnathus fasciatus* | 2E-83 |
| Contig1284 | Keratin, type I cytoskeletal 13-like | XP_003442483.1 | *Oreochromis niloticus* | 1E-29 |
| Contig07352 | Dentin sialophosphoprotein-like | XP_003447913.1 | *Oreochromis niloticus* | 7E-12 |
| Contig01673 | Collagen alpha-1(IV) chain-like | XP_003961761.1 | *Takifugu rubripes* | 3E-159 |
| Contig08806 | Intermediate light meromyosin | ABR19833.1 | *Ctenopharyngodon idella* | 1E-137 |
| Contig05635 | Alpha-2-HS-glycoprotein-like | XP_004078952.1 | *Oryzias latipes* | 2E-111 |
| Contig02752 | Cytosolic phospholipase A2 zeta-like | XP_003446488.1 | *Oreochromis niloticus* | 0 |
| Contig21676 | Beta-microseminoprotein-like | XP_003452718.1 | *Oreochromis niloticus* | 7E-33 |
| Contig05350 | Uncharacterized protein LOC102080835 | XP_005476593.1 | *Oreochromis niloticus* | 2E-71 |
| Contig659 | Epidermis-type lipoxygenase 3-like | XP_003449933.1 | *Oreochromis niloticus* | 0 |
| Contig02069 | Na/Pi cotransport system protein | AAB16821.1 | *Pseudopleuronectes americanus* | 0 |
| **Upregulated genes in premetamorphic stage 5 compared to metamorphic climax stage9C** | |  |  |  |
| Contig05607 | Type I keratin isoform 2 | BAF56914.1 | *Solea senegalensis* | 1E-145 |
| Contig494 | High choriolytic enzyme 1-like | XP_003456934.1 | *Oreochromis niloticus* | 4E-87 |
| Contig01773 | Meprin A subunit beta-like | XP_003450581.1 | *Oreochromis niloticus* | 0 |
| Contig00039 | Fatty acid synthase isoform 2 | XP_003454104.1 | *Oreochromis niloticus* | 0 |
| Contig01282 | Hypothetical protein LOC100691699 | XP_003439216.1 | *Oreochromis niloticus* | 1E-166 |

**Additional file 10f:** List of significantly upregulated genes between metamorphic climax stage 9A and prometamorphic stage 7 as determined by a linear model in Bayseq with a Benjamin-Hochberg adjustment for multiple testing analysis with a cut-off set at 0.05 (FDR<0.05). Contig name, Gene name, Accession number (no.), Organism and E-value are shown for each gene.

| **Upregulated genes in metamorphic climax stage 9A compared to prometamorphic stage 7** | |  |  |  |
| --- | --- | --- | --- | --- |
| ***Contig ID*** | ***Gene name*** | ***Accession no.*** | ***Organism*** | ***E-value*** |
| Contig1159 | Collagen alpha-1(I) chain-like | XP_003460741.1 | *Cavia porcellus* | 1E-62 |

**Additional file 10g:** List of significantly upregulated genes between metamorphic climax stage 9A and metamorphic proclimax stage 8 as determined by a linear model in Bayseq with a Benjamin-Hochberg adjustment for multiple testing analysis with a cut-off set at 0.05 (FDR<0.05). Contig name, Gene name, Accession number (no.), Organism and E-value are shown for each gene.

| **Upregulated genes in metamorphic climax stage 9A compared to metamorphic proclimax stage 8** | |  |  |  |
| --- | --- | --- | --- | --- |
| ***Contig ID*** | ***Gene name*** | ***Accession no.*** | ***Organism*** | ***E-value*** |
| Contig02895 | Oreochromis niloticus UPF0661 TPR repeat-containing protein C16D10.05c-like | XM_005472393.1 | *Oreochromis niloticus* | 4E-177 |

**Additional file 10h:** List of significantly upregulated genes between metamorphic climax stage 9A and metamorphic climax stage 9C as determined by a linear model in Bayseq with a Benjamin-Hochberg adjustment for multiple testing analysis with a cut-off set at 0.05 (FDR<0.05). Contig name, Gene name, Accession number (no.), Organism and E-value are shown for each gene.

| **Upregulated genes in metamorphic climax stage 9A compared to metamorphic climax stage 9C** | |  |  |  |
| --- | --- | --- | --- | --- |
| ***Contig ID*** | ***Gene name*** | ***Accession no.*** | ***Organism*** | ***E-value*** |
| Contig00039 | Fatty acid synthase isoform 2 | XP_003454104.1 | *Oreochromis niloticus* | 0 |
| **Upregulated genes in metamorphic climax stage 9C compared to metamorphic climax stage 9A** | |  |  |  |
| Contig02069 | Na/Pi cotransport system protein | AAB16821.1 | *Pseudopleuronectes americanus* | 0 |
| Contig24460 | Estrogen-regulated protein | ACX94453.1 | *Sparus aurata* | 1E-28 |
| lchead_c7171 | Cytochrome c oxidase subunit 4 isoform 2, mitochondrial | P80971.2 | *Thunnus obesus* | 2E-32 |
| Contig21525 | Alpha-globin 1 | ACI28515.1 | *Hippoglossus hippoglossus* | 6E-70 |
| Contig07132 | Betaine-homocysteine S-methyltransferase 1 | ACQ58374.1 | *Anoplopoma fimbria* | 0 |
| Contig02419 | Epinephelus coioides hypoxia-inducible factor 4 alpha (HIF-4a) mrna | AY735011.1 | *Epinephelus coioides* | 0 |
| Contig08214 | Takifugu rubripes Krueppel-like factor 3-like (LOC101072211) | XM_003978083.1 | *Takifugu rubripes* | 4E-113 |
| Contig02675 | Dehydrogenase/reductase SDR family member 7C-A-like | XP_003438705.1 | *Oreochromis niloticus* | 1E-149 |

**Additional file 10i:** List of significantly upregulated genes between metamorphic climax stage 9B and metamorphic proclimax stage 8 as determined by a linear model in Bayseq with a Benjamin-Hochberg adjustment for multiple testing analysis with a cut-off set at 0.05 (FDR<0.05). Contig name, Gene name, Accession number (no.), Organism and E-value are shown for each gene.

| **Upregulated genes in metamorphic climax stage 9B compared to metamorphic proclimax stage 8** | |  |  |  |
| --- | --- | --- | --- | --- |
| ***Contig ID*** | ***Gene name*** | ***Accession no.*** | ***Organism*** | ***E-value*** |
| lcgut_c20403 | Warm temperature acclimation-related 65 kda protein | ACU86959.1 | *Paralichthys olivaceus* | 5E-41 |

**Additional file 10j:** List of significantly upregulated genes between metamorphic climax stage 9C and prometamorphic stage 7 as determined by a linear model in Bayseq with a Benjamin-Hochberg adjustment for multiple testing analysis with a cut-off set at 0.05 (FDR<0.05). Contig name, Gene name, Accession number (no.), Organism and E-value are shown for each gene.

| **Upregulated genes in metamorphic climax stage 9C compared to prometamorphic stage 7** | |  |  |  |
| --- | --- | --- | --- | --- |
| ***Contig ID*** | ***Gene name*** | ***Accession no.*** | ***Organism*** | ***E-value*** |
| Contig02675 | Dehydrogenase/reductase SDR family member 7C-A-like | XP_003438705.1 | *Oreochromis niloticus* | 1E-149 |
| Contig02069 | Na/Pi cotransport system protein | AAB16821.1 | *Pseudopleuronectes americanus* | 0 |
| Contig07132 | Betaine--homocysteine S-methyltransferase 1 | ACQ58374.1 | *Anoplopoma fimbria* | 0 |
| lcgut_c20403 | Warm temperature acclimation-related 65 kda protein | ACU86959.1 | *Paralichthys olivaceus* | 5E-41 |
| Contig21525 | Alpha-globin 1 | ACI28515.1 | *Hippoglossus hippoglossus* | 6E-70 |
| Contig00708 | Von Willebrand factor A domain-containing protein 7-like isoform X1 | XP_003448088.2 | *Oreochromis niloticus* | 0 |
| Contig02581 | Ryanodine receptor 3 | XP_001922113.2 | *Danio rerio* | 0 |

**Additional file 10k:** List of top most significantly upregulated genes between prometamorphic stage 7 and juvenile stage as determined by a linear model in Bayseq with a Benjamin-Hochberg adjustment for multiple testing analysis with a cut-off set at 0.05 (FDR<0.05). Contig name, Gene name, Accession number (no.), Organism and E-value are shown for each gene.

| **Upregulated genes in prometamorphic stage 7 compared to juvenile** | |  |  |  |
| --- | --- | --- | --- | --- |
| ***Contig ID*** | ***Gene name*** | ***Accession no.*** | ***Organism*** | ***E-value*** |
| Contig1085 | DNA-directed RNA polymerase I subunit RPA43-like isoform X1 | XP_003457324.1 | *Oreochromis niloticus* | 1E-119 |
| Contig26841 | Hypothetical protein | XP_002723892.1 | *Oryctolagus cuniculus* | 5E-26 |
| **Upregulated genes in juvenile compared to prometamorphic stage 7** | |  |  |  |
| Contig1356 | Beta actin | ACZ63697.1 | *Hippoglossus hippoglossus* | 4E-74 |
| Contig899 | 40S ribosomal protein S3 | AAT01919.1 | *Pseudopleuronectes americanus* | 8E-46 |
| Contig14523 | Chitinase1 | BAD15059.1 | *Paralichthys olivaceus* | 1E-134 |
| Contig04218 | Acidic mammalian chitinase-like | XP_003459087.1 | *Oreochromis niloticus* | 0 |
| Contig10095 | AF156788_1 pepsinogen A form iib precursor | AAD56284.1 | *Pseudopleuronectes americanus* | 1E-178 |
| Contig06093 | Betain homocystein methyltransferase | CBA10400.1 | *Platichthys flesus* | 0 |
| Contig32428 | Hypothetical protein EGM_09444, partial | EHH59357.1 | *Macaca fascicularis* | 1E-11 |
| Contig1703 | Tubulin alpha-5 chain-like | XP_003641692.1 | *Gallus gallus* | 6E-67 |
| Contig1301 | Flocculation protein FLO11-like isoform X5 | XP_004538412.1 | *Maylandia zebra* | 7E-50 |
| Contig30057 | 40S ribosomal protein S15-like | XP_003437814.1 | *Oreochromis niloticus* | 7E-15 |
| Contig21525 | Alpha-globin 1 | ACI28515.1 | *Hippoglossus hippoglossus* | 6E-70 |
| Contig12585 | Elastase 3 precursor | BAA82369.2 | *Paralichthys olivaceus* | 1E-150 |
| Contig09675 | Ubiquitin carboxyl-terminal hydrolase 28-like | XP_003453773.1 | *Oreochromis niloticus* | 1E-129 |
| Contig00708 | Von Willebrand factor A domain-containing protein 7-like isoform X1 | XP_003448088.2 | *Oreochromis niloticus* | 0 |
| Contig02265 | Creatine kinase U-type, mitochondrial-like | XP_003455474.1 | *Oreochromis niloticus* | 0 |
| Contig08947 | Unnamed protein product | CAG06445.1 | *Tetraodon nigroviridis* | 1E-139 |
| Contig16328 | Actin, partial | AEM53400.1 | *Pontoscolex corethrurus* | 5E-24 |
| Contig26844 | Ras-related protein Rab-11B-like | XP_003461453.1 | *Cavia porcellus* | 4E-33 |
| Contig05598 | CCAAT/enhancer-binding protein delta-like | XP_003438154.1 | *Oreochromis niloticus* | 1E-111 |
| Contig15968 | Myosin-binding protein H-like | XP_003448314.1 | *Oreochromis niloticus* | 2E-39 |
| Contig01329 | Arrestin domain-containing protein 2-like isoformx2 | XP_003449080.1 | *Oreochromis niloticus* | 0 |
| Contig07184 | Myelin basic protein-like | XP_003443685.1 | *Oreochromis niloticus* | 8E-29 |
| Contig09813 | Uncharacterized protein LOC100690208 isoform X1 | XP_003450545.1 | *Oreochromis niloticus* | 1E-110 |
| Contig20583 | Cellular retinol-binding protein type II | AEM37665.1 | *Epinephelus bruneus* | 1E-65 |
| Contig14695 | Tenebrosin-C-like | XP_003448747.1 | *Oreochromis niloticus* | 1E-60 |
| Contig23899 | Tributyltin binding protein type 2 | BAF56478.1 | *Paralichthys olivaceus* | 2E-56 |
| Contig02070 | Protein TFG-like | XP_003451650.1 | *Oreochromis niloticus* | 1E-106 |
| Contig18680 | Uncharacterized protein LOC101071481 | XP_003977893.1 | *Oryzias latipes* | 2E-24 |
| Contig04032 | Argininosuccinate lyase-like | XP_003447016.1 | *Oreochromis niloticus* | 0 |
| Contig07430 | Claudin 31 | AAT64077.1 | *Takifugu rubripes* | 4E-81 |
| Contig19916 | L-rhamnose-binding lectin CSL2-like | XP_003455367.1 | *Oreochromis niloticus* | 4E-68 |
| Contig19678 | Putative ferric-chelate reductase 1 | AEH76583.1 | *Epinephelus bruneus* | 7E-37 |
| Contig06206 | Inhibitor of nuclear factor kappa B alpha | ABO40445.1 | *Siniperca chuatsi* | 1E-131 |

**Additional file 10l:** List of top most significantly upregulated genes between metamorphic proclimax stage 8 and Juvenile stage as determined by a linear model in Bayseq with a Benjamin-Hochberg adjustment for multiple testing analysis with a cut-off set at 0.05 (FDR<0.05). Contig name, Gene name, Accession number (no.), Organism and E-value are shown for each gene.

| **Upregulated genes in juvenile compared to metamorphic proclimax stage 8** | |  |  |  |
| --- | --- | --- | --- | --- |
| ***Contig ID*** | ***Gene name*** | ***Accession no.*** | ***Organism*** | ***E-value*** |
| Contig1303 | Cytochrome oxidase subunit I | CAO79645.1 | *Hippoglossus hippoglossus* | 2E-34 |
| Contig1356 | Beta actin | ACZ63697.1 | *Hippoglossus hippoglossus* | 4E-74 |
| Contig00796 | Kelch-like protein 31-like | XP_003441041.1 | *Oreochromis niloticus* | 0 |
| Contig1808 | Cytochrome c oxidase subunit III | YP_001403135.1 | *Hippoglossus hippoglossus* | 9E-51 |
| Contig14523 | Chitinase1 | BAD15059.1 | *Paralichthys olivaceus* | 1E-134 |
| Contig03270 | S-adenosylmethionine synthase isoform type-1-like | XP_003448758.1 | *Oreochromis niloticus* | 0 |
| Contig1525 | Ubiquitin | XP_001664267.1 | *Aedes aegypti* | 5E-31 |
| Contig26146 | 40S ribosomal protein S15 | ACN10008.1 | *Salmo salar* | 2E-24 |
| Contig01545 | Aspartate aminotransferase, cytoplasmic-like | XP_003454182.1 | *Oreochromis niloticus* | 0 |
| Contig06359 | Actin, alpha, cardiac muscle 1a | NP_001001409.2 | *Danio rerio* | 0 |
| Contig904 | Eukaryotic translation initiation factor 1B | NP_955882.1 | *Danio rerio* | 8E-42 |
| Contig27668 | Unknown | AAT45249.1 | *Sparus aurata* | 3E-11 |
| Contig1306 | Sarcoplasmic/endoplasmic reticulum calcium atpase 1-like isoform X1 | XP_004575013.1 | *Maylandia zebra* | 4E-37 |
| Contig10095 | Pepsinogen A form IIb precursor | AAD56284.1 | *Pseudopleuronectes americanus* | 1E-178 |
| Contig1207 | Chitinase 3 | BAL14138.1 | *Thunnus orientalis* | 9E-76 |
| Contig02050 | 5-aminolevulinate synthase, nonspecific, mitochondrial-like isoform X1 | XP_003454600.1 | *Oreochromis niloticus* | 0 |
| Contig968 | Ribosomal protein S2-like isoform 3 | XP_002727213.1 | *Rattus norvegicus* | 3E-27 |
| Contig06606 | 60S ribosomal protein L3-like | XP_003456398.1 | *Oreochromis niloticus* | 0 |
| Contig1651 | Chymotrypsinogen 1 | BAL14136.1 | *Thunnus orientalis* | 5E-52 |
| Contig04737 | Protein phosphatase 1 catalytic subunit beta isoform | ABC94584.1 | *Scophthalmus maximus* | 0 |
| Contig08734 | Muscle specific ring finger protein 1-like | NP_001133124.1 | *Salmo salar* | 1E-161 |
| Contig08133 | Induced myeloid leukemia cell differentiation protein Mcl-1 homolog | XP_003450317.1 | *Oreochromis niloticus* | 4E-64 |
| Contig1494 | Beta-actin | ABM92344.1 | *Laternula elliptica* | 2E-44 |
| Contig477 | Nascent polypeptide-associated complex subunit alpha, muscle-specific form isoform X4 | XP_005448685.1 | *Oreochromis niloticus* | 4E-25 |
| Contig1703 | Tubulin alpha-5 chain-like | XP_003641692.1 | *Gallus gallus* | 6E-67 |
| Contig30057 | 40S ribosomal protein S15-like | XP_003437814.1 | *Oreochromis niloticus* | 7E-15 |
| Contig21525 | Alpha-globin 1 | ACI28515.1 | *Hippoglossus hippoglossus* | 6E-70 |
| Contig21976 | Rasgap-activating-like protein 1 | XP_003445666.1 | *Oreochromis niloticus* | 7E-12 |
| Contig12585 | Elastase 3 precursor | BAA82369.2 | *Paralichthys olivaceus* | 1E-150 |
| Contig09675 | Ubiquitin carboxyl-terminal hydrolase 28-like | XP_003453773.1 | *Oreochromis niloticus* | 1E-129 |
| Contig02265 | Creatine kinase U-type, mitochondrial-like | XP_003455474.1 | *Oreochromis niloticus* | 0 |
| Contig27523 | 40S ribosomal protein S3 | AAT01919.1 | *Pseudopleuronectes americanus* | 2E-19 |
| Contig16328 | Actin, partial | AEM53400.1 | *Pontoscolex corethrurus* | 5E-24 |
| Contig495 | Actin, alpha 1, skeletal muscle | NP_001006709.1 | *Xenopus (Silurana) tropicalis* | 0 |
| Contig09238 | Homocysteine-responsive endoplasmic reticulum-resident ubiquitin-like domain member 2 protein-like | XP_003450304.1 | *Oreochromis niloticus* | 3E-50 |
| Contig26844 | Ras-related protein Rab-11B-like | XP_003461453.1 | *Cavia porcellus* | 4E-33 |
| Contig09231 | Hematological and neurological expressed 1 protein-like | XP_003452909.1 | *Oreochromis niloticus* | 1E-58 |
| Contig30975 | Cytochrome b | YP_001403141.1 | *Hippoglossus hippoglossus* | 2E-20 |
| Contig09962 | Fish-egg lectin | BAL61198.1 | *Oplegnathus fasciatus* | 2E-58 |
| Contig23899 | Tributyltin binding protein type 2 | BAF56478.1 | *Paralichthys olivaceus* | 2E-56 |

**Additional file 10m:** List of top most significantly upregulated genes between metamorphic climax stage 9A and juvenile stage as determined by a linear model in Bayseq with a Benjamin-Hochberg adjustment for multiple testing analysis with a cut-off set at 0.05 (FDR<0.05). Contig name, Gene name, Accession number (no.), Organism and E-value are shown for each gene.

| **Upregulated genes in metamorphic climax stage 9A compared to juvenile** | |  |  |  |
| --- | --- | --- | --- | --- |
| ***Contig ID*** | ***Gene name*** | ***Accession no.*** | ***Organism*** | ***E-value*** |
| Contig26888 | ATP synthase subunit beta | XP_003627732.1 | *Medicago truncatula* | 2E-38 |
| **Upregulated genes in juvenile compared to metamorphic climax stage 9A** | |  |  |  |
| Contig1352 | Actin alpha skeletal muscle | ACM41845.1 | *Epinephelus coioides* | 1E-114 |
| Contig1500 | Ribosomal protein, large, P0 | NP_001080134.1 | *Xenopus laevis* | 6E-50 |
| Contig1162 | Cytochrome oxidase subunit I | CAO79645.1 | *Hippoglossus hippoglossus* | 1E-112 |
| Contig20504 | Type-4 ice-structuring protein | Q8JI37.1 | *Paralichthys olivaceus* | 3E-40 |
| Contig28671 | Trypsinogen Flou2 | AAY23358.1 | *Hippoglossus hippoglossus* | 5E-43 |
| Contig25330 | Sarcoendoplasmic reticulum calcium atpase | ABG90496.1 | *Silurus lanzhouensis* | 2E-28 |
| Contig1056 | Sarcoplasmic/endoplasmic reticulum calcium atpase 1-like isoform X1 | XP_003454037.1 | *Oreochromis niloticus* | 0 |
| Contig24482 | Actin, gamma-enteric smooth muscle-like isoform 1 | XP_003201904.1 | *Meleagris gallopavo* | 2E-26 |
| Contig05820 | Fish-egg lectin | BAL61198.1 | *Oplegnathus fasciatus* | 2E-83 |
| Contig25352 | Alpha-cardiac actin | AAA37165.1 | *Mus musculus* | 1E-22 |
| Contig1356 | Beta actin | ACZ63697.1 | *Hippoglossus hippoglossus* | 4E-74 |
| Contig500 | Ferritin high chain | CAR66078.1 | *Trematomus bernacchii* | 4E-93 |
| Contig899 | 40S ribosomal protein S3 | AAT01919.1 | *Pseudopleuronectes americanus* | 8E-46 |
| Contig1808 | Cytochrome c oxidase subunit III | YP_001403135.1 | *Hippoglossus hippoglossus* | 9E-51 |
| Contig14523 | Chitinase1 | BAD15059.1 | *Paralichthys olivaceus* | 1E-134 |
| Contig00193 | Kelch-like protein 31-like | XP_003458597.1 | *Oreochromis niloticus* | 0 |
| Contig1525 | Ubiquitin | XP_001664267.1 | *Aedes aegypti* | 5E-31 |
| Contig26146 | 40S ribosomal protein S15 | ACN10008.1 | *Salmo salar* | 2E-24 |
| Contig01545 | Aspartate aminotransferase, cytoplasmic-like | XP_003454182.1 | *Oreochromis niloticus* | 0 |
| Contig06359 | Actin, alpha, cardiac muscle 1a | NP_001001409.2 | *Danio rerio* | 0 |
| Contig10095 | AF156788_1 pepsinogen A form iib precursor | AAD56284.1 | *Pseudopleuronectes americanus* | 1E-178 |
| Contig1062 | Cytochrome b | YP_001403141.1 | *Hippoglossus hippoglossus* | 2E-62 |
| Contig06093 | Betain homocystein methyltransferase | CBA10400.1 | *Platichthys flesus* | 0 |
| Contig06606 | 60S ribosomal protein L3-like | XP_003456398.1 | *Oreochromis niloticus* | 0 |
| Contig08242 | Leukocyte elastase inhibitor-like isoform X1 | XP_003457347.1 | *Oreochromis niloticus* | 5E-77 |
| Contig08734 | Muscle specific ring finger protein 1-like | NP_001133124.1 | *Salmo salar* | 1E-161 |
| Contig08657 | Myosin-10-like | XP_003448012.1 | *Oreochromis niloticus* | 1E-145 |
| Contig08133 | Induced myeloid leukemia cell differentiation protein Mcl-1 homolog | XP_003450317.1 | *Oreochromis niloticus* | 4E-64 |
| Contig05461 | Nebulin-like | ACH85357.1 | *Salmo salar* | 7E-153 |
| Contig477 | Nascent polypeptide-associated complex subunit alpha, muscle-specific form isoform X4 | XP_005448685.1 | *Oreochromis niloticus* | 4E-25 |
| Contig1703 | Tubulin alpha-5 chain-like | XP_003641692.1 | *Gallus gallus* | 6E-67 |
| Contig03898 | Cathepsin L | ABJ99858.1 | *Hippoglossus hippoglossus* | 0 |
| Contig21525 | Alpha-globin 1 | ACI28515.1 | *Hippoglossus hippoglossus* | 6E-70 |
| Contig12585 | Elastase 3 precursor | BAA82369.2 | *Paralichthys olivaceus* | 1E-150 |
| Contig495 | Actin, alpha 1, skeletal muscle | NP_001006709.1 | *Xenopus tropicalis* | 0 |
| Contig26844 | Ras-related protein Rab-11B-like | XP_003461453.1 | *Cavia porcellus* | 4E-33 |
| Contig07011 | Ubiquitin carboxyl-terminal hydrolase 28-like | XP_003453773.1 | *Oreochromis niloticus* | 1E-124 |
| Contig09813 | Uncharacterized protein LOC100690208 | XP_003450545.1 | *Oreochromis niloticus* | 6E-140 |

**Additional file 10n:** List of top most significantly upregulated genes between metamorphic climax stage 9B and Juvenile stage as determined by a linear model in Bayseq with a Benjamin-Hochberg adjustment for multiple testing analysis with a cut-off set at 0.05 (FDR<0.05). Contig name, Gene name, Accession number (no.), Organism and E-value are shown for each gene.

| **Upregulated genes in metamorphic climax stage 9B compared to juvenile** | |  |  |  |
| --- | --- | --- | --- | --- |
| ***Contig ID*** | ***Gene name*** | ***Accession no.*** | ***Organism*** | ***E-value*** |
| Contig00749 | Cdh1-d | AAL31950.1 | *Gallus gallus* | 2E-27 |
| Contig26841 | Hypothetical protein | XP_002723892.1 | *Oryctolagus cuniculus* | 5E-26 |
| **Upregulated genes in juvenile compared to metamorphic climax stage 9B** | |  |  |  |
| Contig1303 | Cytochrome oxidase subunit I | CAO79645.1 | *Hippoglossus hippoglossus* | 2E-34 |
| Contig1356 | Beta actin | ACZ63697.1 | *Hippoglossus hippoglossus* | 4E-74 |
| Contig1808 | Cytochrome c oxidase subunit III | YP_001403135.1 | *Hippoglossus hippoglossus* | 9E-51 |
| Contig434 | WD repeat-containing protein 20-like | XP_003459593.1 | *Oreochromis niloticus* | 1E-173 |
| Contig08734 | Muscle specific ring finger protein 1-like | NP_001133124.1 | *Salmo salar* | 1E-161 |
| Contig08133 | Induced myeloid leukemia cell differentiation protein Mcl-1 homolog | XP_003450317.1 | *Oreochromis niloticus* | 4E-64 |
| Contig32428 | Hypothetical protein EGM_09444, partial | EHH59357.1 | *Macaca fascicularis* | 1E-11 |
| Contig1703 | Tubulin alpha-5 chain-like | XP_003641692.1 | *Gallus gallus* | 6E-67 |
| Contig21976 | Rasgap-activating-like protein 1 | XP_003445666.1 | *Oreochromis niloticus* | 7E-12 |
| Contig12585 | Elastase 3 precursor | BAA82369.2 | *Paralichthys olivaceus* | 1E-150 |
| Contig02012 | Protein Tob1-like | XP_003452855.1 | *Oreochromis niloticus* | 1E-162 |
| Contig09675 | Ubiquitin carboxyl-terminal hydrolase 28-like | XP_003453773.1 | *Oreochromis niloticus* | 1E-129 |
| Contig13939 | Proteasome subunit beta type-6-like | XP_003456484.1 | *Oreochromis niloticus* | 1E-113 |
| Contig22705 | Ribosomal protein L29 | CBN80862.1 | *Dicentrarchus labrax* | 8E-21 |
| Contig14585 | Heat shock protein beta-11-like | XP_003446493.1 | *Oreochromis niloticus* | 2E-71 |
| Contig03419 | Twinfilin-2-like | XP_003448434.1 | *Oreochromis niloticus* | 0 |
| Contig27523 | 40S ribosomal protein S3 | AAT01919.1 | *Pseudopleuronectes americanus* | 2E-19 |
| Contig16328 | Actin, partial | AEM53400.1 | *Pontoscolex corethrurus* | 5E-24 |
| Contig07754 | Membrane-spanning 4-domains subfamily A member 4A | ACO09823.1 | *Osmerus mordax* | 1E-38 |
| Contig26844 | Ras-related protein Rab-11B-like | XP_003461453.1 | *Cavia porcellus* | 4E-33 |
| Contig01134 | Transmembrane protease serine 9-like | XP_005469283.1 | *Oreochromis niloticus* | 0 |
| Contig05598 | CCAAT/enhancer-binding protein delta-like | XP_003438154.1 | *Oreochromis niloticus* | 1E-111 |
| Contig21640 | ATP synthase-coupling factor 6, mitochondrial precursor | ACQ58964.1 | *Anoplopoma fimbria* | 8E-48 |
| Contig938 | Ras-related protein Rab-10-like | XP_003446295.1 | *Oreochromis niloticus* | 1E-110 |
| Contig02215 | Galectin-3-like | XP_003445459.1 | *Oreochromis niloticus* | 1E-57 |
| Contig27091 | 60S ribosomal protein L34 | AEG78366.1 | *Epinephelus coioides* | 1E-25 |
| Contig30975 | Cytochrome b | YP_001403141.1 | *Hippoglossus hippoglossus* | 2E-20 |
| Contig07880 | Calumenin-A-like | XP_003448805.1 | *Oreochromis niloticus* | 1E-106 |
| Contig08853 | Uncharacterized protein | CBN82110.1 | *Dicentrarchus labrax* | 3E-54 |
| Contig07526 | Vesicle-associated membrane protein-associated protein A | ACQ58821.1 | *Anoplopoma fimbria* | 1E-107 |
| Contig21841 | CD59 glycoprotein-like | XP_004085111.1 | *Oryzias latipes* | 9E-28 |
| Contig14695 | Tenebrosin-C-like | XP_003448747.1 | *Oreochromis niloticus* | 1E-60 |
| Contig01833 | Signal transducer and activator of transcription 1 | ABS19629.1 | *Paralichthys olivaceus* | 0 |

**Additional file 10o:** List of top most significantly upregulated genes between metamorphic climax stage 9C and Juvenile stage as determined by a linear model in Bayseq with a Benjamin-Hochberg adjustment for multiple testing analysis with a cut-off set at 0.05 (FDR<0.05). Contig name, Gene name, Accession number (no.), Organism and E-value are shown for each gene.

| **Upregulated genes in metamorphic climax stage 9C compared to juvenile** | |  |  |  |
| --- | --- | --- | --- | --- |
| ***Contig ID*** | ***Gene name*** | ***Accession no.*** | ***Organism*** | ***E-value*** |
| Contig1159 | Collagen alpha-1(I) chain-like | XP_003460741.1 | *Cavia porcellus* | 1E-62 |
| Contig14322 | CD59 glycoprotein-like | XP_003460314.1 | *Oreochromis niloticus* | 2E-43 |
| Contig1362 | Rrna promoter binding protein-like | XP_002724111.1 | *Oryctolagus cuniculus* | 9E-27 |
| Contig13553 | 1-acyl-sn-glycerol-3-phosphate acyltransferase epsilon-like | XP_003441167.1 | *Oreochromis niloticus* | 2E-93 |
| **Upregulated genes in juvenile compared to metamorphic climax stage 9C** | |  |  |  |
| Contig08242 | Leukocyte elastase inhibitor-like isoform X1 | XP_003457347.1 | *Oreochromis niloticus* | 5E-77 |
| Contig08657 | Myosin-10-like | XP_003448012.1 | *Oreochromis niloticus* | 1E-145 |
| Contig08133 | Induced myeloid leukemia cell differentiation protein Mcl-1 homolog | XP_003450317.1 | *Oreochromis niloticus* | 4E-64 |
| Contig32428 | Hypothetical protein EGM_09444, partial | EHH59357.1 | *Macaca fascicularis* | 1E-11 |
| Contig477 | Nascent polypeptide-associated complex subunit alpha, muscle-specific form isoform X4 | XP_005448685.1 | *Oreochromis niloticus* | 4E-25 |
| Contig1703 | Tubulin alpha-5 chain-like | XP_003641692.1 | *Gallus gallus* | 6E-67 |
| Contig30057 | 40S ribosomal protein S15-like | XP_003437814.1 | *Oreochromis niloticus* | 7E-15 |
| Contig08838 | Carbonyl reductase-like 20beta-hydroxysteroid dehydrogenase | ACK99046.1 | *Solea senegalensis* | 1E-130 |
| Contig13939 | Proteasome subunit beta type-6-like | XP_003456484.1 | *Oreochromis niloticus* | 1E-113 |
| Contig27523 | 40S ribosomal protein S3 | AAT01919.1 | *Pseudopleuronectes americanus* | 2E-19 |
| Contig16328 | Actin, partial | AEM53400.1 | *Pontoscolex corethrurus* | 5E-24 |
| Contig09238 | Homocysteine-responsive endoplasmic reticulum-resident ubiquitin-like domain member 2 protein-like | XP_003450304.1 | *Oreochromis niloticus* | 3E-50 |
| Contig26844 | Ras-related protein Rab-11B-like | XP_003461453.1 | *Cavia porcellus* | 4E-33 |
| Contig01134 | Polyserase-2-like | XP_003442512.1 | *Oreochromis niloticus* | 1E-126 |
| Contig07011 | Ubiquitin carboxyl-terminal hydrolase 28-like | XP_003453773.1 | *Oreochromis niloticus* | 1E-124 |
| Contig09231 | Hematological and neurological expressed 1 protein-like | XP_003452909.1 | *Oreochromis niloticus* | 1E-58 |
| lchead_c50924 | Beta-crystallin A2-like isoform 1 | XP_003443383.1 | *Oreochromis niloticus* | 1E-114 |
| Contig01786 | Tyrosine aminotransferase | XP_003439428.1 | *Oreochromis niloticus* | 0 |
| Contig07880 | Calumenin-A-like | XP_003448805.1 | *Oreochromis niloticus* | 1E-106 |
| Contig01833 | Signal transducer and activator of transcription 1 | ABS19629.1 | *Paralichthys olivaceus* | 0 |
| Contig875 | Alpha tubulin | AAG15324.1 | *Notothenia coriiceps* | 7E-99 |
| Contig07730 | Translation initiation factor eif-2B precursor | AAF61750.1 | *Sciaenops ocellatus* | 0 |
| Contig18680 | Uncharacterized protein LOC101071481 | XP_003977893.1 | *Takifugu rubripes* | 2E-24 |
| Contig31578 | Unnamed protein product | CAF90797.1 | *Tetraodon nigroviridis* | 9E-12 |
| Contig01775 | Cullin-associated NEDD8-dissociated protein 1-like isoform X1 | XP_003451898.1 | *Oreochromis niloticus* | 0 |
| Contig05847 | 14-3-3 protein beta/alpha-1 | NP_001117940.1 | *Oncorhynchus mykiss* | 1E-108 |
| Contig03424 | Phenylalanine-4-hydroxylase | XP_003448110.1 | *Oreochromis niloticus* | 0 |
| Contig19916 | L-rhamnose-binding lectin CSL2-like | XP_003455367.1 | *Oreochromis niloticus* | 4E-68 |
| Contig19678 | Putative ferric-chelate reductase 1 | AEH76583.1 | *Epinephelus bruneus* | 7E-37 |
| Contig1780 | 40S ribosomal protein s5 | ADX97209.1 | *Perca flavescens* | 4E-20 |
| Contig22533 | ATP synthase subunit alpha, mitochondrial-like | XP_003213404.1 | *Meleagris gallopavo* | 1E-20 |
| Contig12770 | NHP2-like protein 1 | ACQ58311.1 | *Anoplopoma fimbria* | 3E-55 |
| Contig04309 | Purine nucleoside phosphorylase 5a | NP_998476.1 | *Danio rerio* | 1E-153 |
