# Supplementary material for: The transcriptome of metamorphosing flatfish
Source: BMC Genomics. 2016 May 27;17:413. doi: 10.1186/s12864-016-2699-x (PMC4884423; doi:10.1186/s12864-016-2699-x)
Supplement: Additional file 11: — Expression profile of putative TH-responsive transcripts that had a differential expression between Atlantic halibut metamorphic stages (7–9C) and the juvenile. Expression levels are represented as log2 of fold-change (juvenile/metamorphic stages expression levels). (DOCX 41 kb) [file 12864_2016_2699_MOESM11_ESM.docx]

**Additional file 11**: Expression profile of putative TH-responsive transcripts with differential expression between juvenile and metamorphic stages. Expression levels profile is measured as the log2 of fold change (juvenile/metamorphic stages expression levels).

|  |  | **log2 fold change (juvenile versus metamorphic stage)** | | | | |
| --- | --- | --- | --- | --- | --- | --- |
| **Gene name** | **Gene symbol** | **Stage7** | **Stage8** | **Stage9A** | **Stage9B** | **Stage9C** |
| Coronin, actin binding protein, 1C | CORO1C | -4.8 | -3.5 | -2.3 | -4.2 | -5.2 |
| Cyclin B1 | CCNB1 | -4.7 | 0 | 0 | 0 | -4.3 |
| Regulator of chromosome condensation 1 | RCC1 | -4 | -3.5 | 0 | 0 | -4.5 |
| Olfactomedin 4 | OLM4 | -4.5 | -3.8 | -1.6 | 0 | -4.5 |
| High mobility group protein-1 | HMG1 | -6.5 | -3.1 | -1.6 | 0 | -4.6 |
| Alkaline phosphatase | ALO | -4.3 | -3.7 | 0 | -3.7 | -3.7 |
| Chromodomain helicase DNA binding protein 4 | CHD4 | -4.9 | -3.6 | 0 | -3.3 | -3.6 |
| Mitogen-activated protein kinase kinase 1 | MAP2K1 | -3.7 | -3.7 | -2.3 | -3.7 | -3.7 |
| Ubiquilin | XDRP1 | -5 | -3.8 | -2.8 | -3.6 | 0 |
| C-Jun protein | c-Jun | -4.4 | -4.4 | -1.6 | -3.8 | 0 |
| Protein kinase | PKN | -4 | -3.9 | -2 | -2.3 | -2 |
| Histone deacetylase 1 | HDAC1 | -4.2 | -4.2 | 0 | -3.6 | -2.6 |
| General transcription factor IIH | GTF2H | -4.2 | -4.2 | 0 | -2.6 | 0 |
| Transcription factor AP-2 | TFAP2 | -5.3 | -4.3 | -2.3 | -3.7 | -3.7 |
| hnRNP I-related RNA transport protein  (Polypyrimidine Tract Binding Protein 1) | VgRBP60 | -5 | -4.4 | -1 | -4.4 | -3.1 |
| Acetylcholine receptor subunit 1A | CHRNA1 | -5.8 | -5.4 | -2 | -5.2 | -4 |
| Biglycan | BGN | -5.2 | -2.2 | 0 | -3.6 | 0 |
| RNA helicase II/Gu | DDX21 | -4.7 | -4.3 | -1.6 | -4.6 | -4.2 |
| Chaperonin subunit CCT | CCT | -9.1 | -4.7 | -2.8 | -6 | -4.4 |
| Proto-Oncogene Serine/Threonine-Protein Kinase Pim-1 | PIM1 | -4.4 | -4.4 | -3.5 | -4.4 | -3.8 |
| Aconitase | ACO | -2 | -3.3 | 0 | 0 | 0 |
| NFI-X2 transcription factor | NFIX | -3.9 | -4.9 | 0 | 0 | 0 |
| SRY (sex determining region Y)-box 4 | SOX4 | -3.4 | -4.4 | -2.3 | -2.8 | -2.8 |
| Putative alanine:glyoxylate aminotransferase | AGXT | -4.2 | -3.4 | 0 | 0 | 0 |
| Methyl-CpG binding domain protein 3 | MBD3 | -5 | -5 | -1.6 | 0 | 0 |
| Larval beta-globin | HBB | -5.8 | -5.8 | -2.6 | 0 | 0 |
| Fibronectin | FN | -4.3 | -3.7 | -1 | 0 | 0 |
| Alpha2 Collagen type 1 | COL1A2 | -4.2 | -3.1 | -2.8 | 0 | 0 |
| Secreted protein, acidic, cysteine-rich (osteonectin) | SPARC | -6.4 | -3 | -1.6 | 0 | 0 |
| Myelin proteolipid protein | PLP1 | -6.2 | -5 | -1.6 | 0 | -4.4 |
| Caveolin-3 | CAV3 | -0.3 | 0 | 0 | 0 | 0 |
| Nucleophosmin/nucleoplasmin | NPM | -3.2 | 0 | 0 | 0 | 0 |
| Minichromosome maintenance complex component 2 | MCM2 | -2.6 | 0 | 0 | 0 | 0 |
| Minichromosome Maintenance Complex Component (MCM5/CDC46) | CDC46 | -4.4 | 0 | -2.3 | 0 | 0 |
| Alpha1 Collagen type 1 | COL1A1 | -5.2 | 0 | -2 | -4.5 | -3.4 |
| Cysteine-Rich, Angiogenic Inducer, 61 | CYR61 | -5.5 | -3.9 | -1.6 | -4.9 | -4.9 |
| Aldolase | ALDO | -7.3 | -4.4 | -1.6 | -5.8 | -4.9 |
| BCL2-Associated Athanogene 6 | BAG6 | -3.2 | -3.2 | -2 | -4.2 | -3.2 |
| Heat shock 60 kDa protein (chaperonin) | HSPD1 | -4.7 | -4 | -1 | -7 | -5.6 |
| Poly A-binding protein ABP-EF | ABP-EF | -4.2 | -3.8 | -2 | -5 | -4.2 |
| Cyclin-dependent kinase 7 | CDK7 | -3.6 | -3.2 | -1.6 | -4.6 | -3.6 |
| Lactate dehydrogenase B | LDHB | -6.3 | -4.7 | -1.6 | -7.3 | -5.7 |
| Arginase type II | ARG2 | -7.4 | -3.9 | -2 | -7.4 | -6.1 |
| Tubulin beta | TUBB | -4.2 | -3.3 | -1.6 | -4.2 | -4.2 |
| Nucleolar and coiled-body phosphoprotein | NOLC | -5 | -3.6 | -2.8 | -5 | -5 |
| Glutamate-cysteine ligase , modifier subunit | GCLM | -4.9 | -3.3 | -3.7 | -4.3 | 0 |
| Corticotropin releasing hormone binding protein | CRHBP | -4.6 | 0 | -1.6 | -4.1 | 0 |
| CCAAT/Enhancer Binding Protein (C/EBP),Delta | CEBPD | -7.9 | -6.1 | -2.8 | -8.3 | -5.1 |
| Minichromosome maintenance complex component 7 | MCM7 | -5.4 | -3.3 | 0 | -6.1 | 0 |
| Defender against cell death 1 | DAD1 | -4.8 | 0 | 0 | -5.2 | 0 |
| RNA polymerase I TF UBF | UBF | -2.8 | 0 | 0 | -2.8 | 0 |
| Enhancer of zeste homolog 2 | EZH2 | -2 | 0 | -1.6 | -3.2 | -3.1 |
| Kirsten rat sarcoma viral oncogene homolog | KRAS | 0 | 0 | -1 | -3.6 | -2.9 |
| Collagenase 3 | MMP13 | 0 | 0 | -1 | -4.8 | -3.8 |
| Sulfotransferase family, cytosolic, 2B, member 1 | SULT2B1 | 0 | 0 | 0 | -4.5 | -4 |
| Eukaryotic peptide chain release factor subunit 1 | ETF1 | 0 | 0 | 0 | -5.1 | -3.6 |
| V-Raf-1 Murine Leukemia Viral Oncogene Homolog 1 | cRaf | 0 | 0 | 0 | -4.5 | -4.5 |
| COX assembly mitochondrial protein homolog | KIT | 0 | 0 | 0 | -2.3 | -3.3 |
| Glycine dehydrogenase | GLDC | -4.4 | -4.5 | -4.3 | -4.8 | -4.8 |
| KDEL (Lys-Asp-Glu-Leu) endoplasmic reticulum proteinretention receptor 2 | KDELR2 | -4.5 | -4.6 | -4.6 | -5.9 | -5.9 |
| Sodium/potassium-transporting ATPase subunit alpha-1 isoform a | ATP1A1 | -1.9 | -2.8 | -2.8 | -6.1 | -5.2 |
| Ribose-phosphate pyrophosphokinase 2 isoform 2 | PRPS2 | -1.4 | -3 | -4.2 | -5.2 | -5.2 |
| T-Complex Protein 1 Subunit Alpha | TCP1 | 0 | -3.6 | -2 | -5.2 | -4.8 |
| Alpha-2-macroglobulin precursor | A2M | 0 | -3.1 | -2.8 | -4.9 | -3.5 |
| Aminolevulinate, Delta-, Synthase 1 | ALAS | 0 | -3.9 | -3.7 | -6.1 | -5.2 |
| Fc fragment of IgG binding protein | FCGBP | 0.1 | -4.2 | -2.6 | -6.2 | -5.3 |
| Deoxyribonuclease I-like 3 | DNASE1L3 | 0 | -3.6 | -3.3 | -4 | -4 |
| Histidine triad nucleotide-binding protein 2, mitochondrial precursor | HINT2 | -1 | -3.8 | -1.4 | -4.2 | -4.2 |
| Lysophospholipase-like protein 1 | LYPLAL1 | 0 | 2.7 | 0 | -4 | -3.8 |
| Cytochrome c oxidase subunit 6B1 | COX6B1 | 0 | -3.6 | -3.2 | -5 | -5.1 |
| Ras-related C3 botulinum toxin substrate 2 | RAC2 | -1 | -3.5 | -1.6 | -4.5 | -5.5 |
| 14-3-3 protein zeta/delta | YWHAZ | 0 | -3.1 | -2 | -5 | -6.6 |
| Cathepsin S | CTSS | 0.3 | -5.2 | -3.6 | -5.3 | -6 |
| 2-amino-3-ketobutyrate coenzyme A ligase, mitochondrial isoform 2 precursor | GCAT | 0.2 | -4.2 | -3.5 | -6.7 | -7.7 |
| Gastrula stage epidermal type I cytokeratin | KRT | 0 | 0 | 0 | 0 | -3.5 |
| Nucleoplasmin-like protein NO29 | NO29 | 0.3 | 0 | 0 | 0 | -3.2 |
| Minichromosome maintenance deficient 3 | MCM3 | 0 | 0 | 0 | -3 | 0 |
| Secreted frizzled-related protein 2 | SFRP2 | 0 | 0 | 0 | -3.6 | 0 |
| Phosphoglucomutase-2 | PGM2 | 0 | 0 | 0 | -4.2 | 0 |
| Actin, aortic smooth muscle | ACTA1 | -2.8 | -3.3 | -7.3 | -6.8 | -6.3 |
| Aspartoacylase | ASPA | 0 | -2.9 | -3.9 | -6.9 | -2.8 |
| Fragile X mental retardation protein 1 | FMR1 | 0 | -3.9 | -4 | -4.3 | 0 |
| poly(U)-specific endoribonuclease isoform 2 | ENDOU | 0 | 0 | -1.6 | -3.8 | 0 |
| Replication protein A | RPA1 | 0 | 0 | -1 | -4.7 | 0 |
| B-cell translocation gene 1, anti-proliferative | BTG | 0 | -4.8 | -1.6 | -6.4 | -4.3 |
| Heat shock 70 kDa protein 5 | HSPA5 | 0 | -3.9 | -2 | -5 | -3.7 |
| Beta-hexosaminidase subunit beta preproprotein | HEXB | 1.6 | -5 | -3.9 | -6.9 | -4.9 |
| Stress-70 protein, mitochondrial precursor | HSPA9 | 0 | -4.3 | -1.6 | -5 | -3.7 |
| X-prolyl aminopeptidase (aminopeptidase P) 2, membrane-bound | XPNPEP2 | 0 | -5.2 | -2 | -5.2 | -4.6 |
| Nicotinamide riboside kinase 2 isoform 2 | NMRK2 | 0 | 6 | 2 | 6 | 3.6 |
| Calmodulin | CALM1 | 0.2 | -4.9 | -2.3 | -4.9 | -4.5 |
| T-box transcription factor 2 | TBX2 | 0 | -5 | -1.4 | -3.5 | -3.5 |
| Ras-related protein Rab-11A isoform 1 | RAB11A | 0 | -4.7 | -1.6 | -4.6 | -4.6 |
| Activator of 90 kDa heat shock protein ATPase homolog 1 | AHSA1 | 0 | 0 | -1.6 | -3.6 | -3.7 |
| Protein phosphatase-2A B'epsilon subunit | PPP2R5E | 0 | -4.7 | 0 | 3 | -3.1 |
| Calcium/calmodulin-dependent protein kinase type II subunit beta isoform 3 | CAMK2B | 0 | -3.5 | 0 | -2.6 | -2.6 |
| Glutamine synthetase | GLUL | 0 | -5.7 | -2.6 | -5 | -4.5 |
| Epsin 3 | EPN3 | 0 | -6.2 | 0 | -3.8 | -2.8 |
| Nuclear receptor corepressor 1 | NCOR1 | -3.2 | -4.2 | 0 | -3.6 | 0 |
| Embryonic serine protease-2 | ESP2 | 0 | -6.1 | 0 | -5.2 | 0 |
| Leucine rich repeat containing 20 | LRRC20 | 0 | -3.2 | 0 | -2.6 | 0 |
| COMM domain containing 7 | COMMD7 | 0 | -7.2 | 0 | -2.3 | 0 |
| 3'-phosphoadenosine 5'-phosphosulfate synthase 2 | PAPSS2 | -2 | -2.7 | 0 | -4.6 | 0 |
| Monocarboxylate transporter 7 | SLC16A6 | -2.6 | -4.4 | 0 | -6 | 0 |
| MAPK MPK1 | MPK1 | -3.5 | -4.1 | -1 | -4.5 | -3.5 |
| Thyroid hormone binding protein/pyruvate kinase type M2 | PKM | 0 | -4 | 0 | -5.8 | 0 |
| High affinity copper uptake protein 1 | SLC31A1 | -1 | -3.5 | -3.2 | -3.9 | -2.9 |
| Serine/threonine-protein kinase Nek3 | NEK3 | 0 | -5.2 | -4.2 | -6.5 | 0 |
| Bubblegum-Related Protein (Acyl-CoA Synthetase Bubblegum Family Member 2 or Long-Chain-Fatty-Acid--CoA Ligase ) | BRGL | -0.3 | -3.9 | -1.5 | -4.4 | 0 |
| Ferritin heavy chain | FTH1 | 0.3 | -5.6 | -3.5 | -5.5 | 0 |
| DnaJ homolog subfamily B member 6 isoform b | DNAJB6 | 0 | -5.3 | -1.4 | -3.3 | 0 |
| Myosin regulatory light polypeptide 9 isoform a | MYL9 | 0 | -4.6 | -3.5 | -4 | 0 |
| General transcription factor IIF | GTF2F | -2.3 | -3.9 | 0 | -2.3 | -2.3 |
| TGFB-induced factor homeodomain | TGIF1 | -2.8 | -4.4 | -1 | -2.8 | -2.8 |
| Peptidyl-prolyl cis-trans isomerase FKBP2 precursor | FKBP2 | -2.6 | -4.5 | 0 | -4.2 | -4.2 |
| FOS-like antigen 2 | FOSL2 | -3 | -4.6 | -1.6 | -3 | -4 |
| Actin-Like 6A | ACTL6A | 0 | -4.4 | 0 | 0 | -3.2 |
| Far upstream element (FUSE) binding protein 1 | FUBP1 | 0 | -4 | 0 | 0 | -3.6 |
| RNA binding motif protein 14 | RBM14 | 0 | -3.9 | 0 | 0 | -2.3 |
| Phenylalanyl-tRNA synthetase, alpha subunit | FARSA | 0 | -4.7 | 0 | 0 | -3.8 |
| Es1 protein isoform Ia precursor | ES1 | 0 | -4.4 | 0 | 0 | -4.3 |
| P450 (cytochrome) oxidoreductase | POR | 0.3 | -4.3 | -1 | 0 | -4.2 |
| Glutaredoxin-1 | GLRX | -0.3 | -6.3 | -1.6 | 0 | -3.5 |
| Casein kinase 1-& isoform | CSNK1A | 0 | -5.2 | -2.6 | 0 | -4 |
| phosphatidylethanolamine-binding protein 1 preproprotein | PEBP1 | 0 | -5.2 | -1.6 | 0 | -4.6 |
| Thioredoxin domain-containing protein 2 isoform 1 | TXNDC2 | 0 | -5.2 | -2.3 | 0 | -4.5 |
| High mobility group 20A | HMG20A | 0.1 | -3.7 | -2.6 | 0 | -3.6 |
| Dystroglycan 1 | DAG1 | 0 | -3.5 | -2.3 | 0 | -4.7 |
| Histocompatibility (minor) 13 | HM13 | 0 | -2.8 | 0 | 0 | -4.6 |
| Protein Arginine Methyltransferase 1 | PRMT1 | 0 | -2.3 | 0 | 0 | 0 |
| Cu-Zn superoxide dismutase | SODB | 0 | -3.1 | 0 | 0 | 0 |
| Kruppel-like factor 9 | KLF9 | -4 | -4.6 | -4 | -4 | -4 |
| Myosin-11 isoform SM2A | MYH11 | 0.2 | -3.7 | -1.5 | 0 | 0 |
| Transmembrane protein 79 | TMEM79 | -1 | -5.2 | -4.7 | -4.7 | -4.7 |
| Sarcoplasmic/endoplasmic reticulum calcium ATPase 2 isoform b | ATP2A2 | 1.3 | -7.3 | 0 | 0 | 0 |
| Aldehyde dehydrogenase, mitochondrial isoform 1 precursor | ALDH2 | 0 | -4.9 | -1 | 0 | 0 |
| Solute carrier family 7 (cationic amino acid transporter, y+system), member 3 | SLC7A3 | 0 | -4.8 | -1.3 | 0 | 0 |
| Sodium- and chloride-dependent neutral and basic amino acid transporter B(0+) | SLC6A14 | 0 | 4.1 | -2.6 | 0 | 0 |
| Nucleoside diphosphate kinase 7 isoform a | NME7 | 0 | -3.4 | -3.5 | 0 | -3.3 |
| Calbindin 1, 28 kDa | CALB1 | -5 | -5.5 | -6.2 | -5 | 5 |
| Collagenase 4 | MMP2 | -3.4 | -2.8 | -4.3 | -2.8 | -3.8 |
| Cyclin-dependent kinase inhibitor 1B | CDKN1B | 0 | 0 | -1.6 | 0 | -2.3 |
| REV1, polymerase (DNA directed) | REV1 | 0 | 2.3 | -3.7 | 0 | 0 |
| Myosin regulatory light chain 2, atrial isoform | MYL7 | 0 | 0 | -1.3 | 0 | 0 |
| EH domain-containing protein 4 | EHD4 | 0 | 0 | -1.3 | 0 | 0 |
