# Supplementary material for: The transcriptome of metamorphosing flatfish
Source: BMC Genomics. 2016 May 27;17:413. doi: 10.1186/s12864-016-2699-x (PMC4884423; doi:10.1186/s12864-016-2699-x)
Supplement: Additional file 13: — Reactome pathway analysis for the 145 differential expressed TH-responsive transcripts identified when Atlantic halibut metamorphic stages were compared with the juvenile. Reactome analysis was performed using INTREPROSCAN accession numbers obtained from the functional annotation of the Atlantic halibut transcriptome with Blast2GO. (DOCX 18 kb) [file 12864_2016_2699_MOESM13_ESM.docx]

**Additional file 13**: Reactome pathway analysis for the 145 differential expressed THs-responsive transcripts between juvenile and metamorphic stages. Reactome analysis was performed using the INTREPROSCAN accession numbers obtained from the functional annotation with Blast2GO.

| **Pathway** | **Species** | **IDs in pathway (%)** | **Enrichment (pval)** | **FDR** |
| --- | --- | --- | --- | --- |
|  |  |  |  |  |
| Circadian Clock | *Homo sapiens* | 6 (16%) | 5.00E-10 | 1.10E-08 |
| Cellular responses to stress | *Homo sapiens* | 52 (21%) | 1.54E-06 | 1.69E-05 |
| Cell Cycle | *Homo sapiens* | 75 (15%) | 1.00E-05 | 6.66E-05 |
| DNA Repair | *Homo sapiens* | 14 (13%) | 2.69E-05 | 1.35E-04 |
| Disease | *Homo sapiens* | 176 (15%) | 2.34E-04 | 9.08E-04 |
| Membrane Trafficking | *Homo sapiens* | 26 (19%) | 3.03E-04 | 9.08E-04 |
| DNA Replication | *Homo sapiens* | 14 (14%) | 3.86E-04 | 1.16E-03 |
| Apoptosis | *Homo sapiens* | 25 (16%) | 1.86E-03 | 3.73E-03 |
| Developmental Biology | *Homo sapiens* | 135 (34%) | 1.11E-02 | 2.22E-02 |
| Metabolism | *Homo sapiens* | 141 (9%) | 1.27E-02 | 2.55E-02 |
| Metabolism of proteins | *Homo sapiens* | 89 (15%) | 1.27E-02 | 2.55E-02 |
| Signal Transduction | *Homo sapiens* | 291 (16%) | 1.73E-02 | 3.47E-02 |
