# Supplementary material for: The transcriptome of metamorphosing flatfish
Source: BMC Genomics. 2016 May 27;17:413. doi: 10.1186/s12864-016-2699-x (PMC4884423; doi:10.1186/s12864-016-2699-x)
Supplement: Additional file 14: — Relative gene expression analysis of transcripts involved in the TH cascade during Atlantic halibut development (stage 5 to juvenile; n = 5 per stage) using quantitative RT-PCR (qPCR). Thyroglobulin (Tg), TH receptor alpha A (TRαA), TH receptor alpha B (TRαB), TH receptor beta (TRβ), deiodinase 3 (DIO3), deiodinase 2 (DIO2), deiodinase 1 (DIO1), monocarboxylate transporter 8 (MCT8), and monocarboxylate transporter 10 (MCT10) gene expression. Results are presented as mean ± SEM of the candidate gene expression, normalized using the geometric mean of the reference genes RPS4 and EF1A1. Significant difference (p < 0.05; one-way ANOVA) of normalized transcript expression between stages are indicated by different letters. (PDF 200 kb) [file 12864_2016_2699_MOESM14_ESM.pdf]

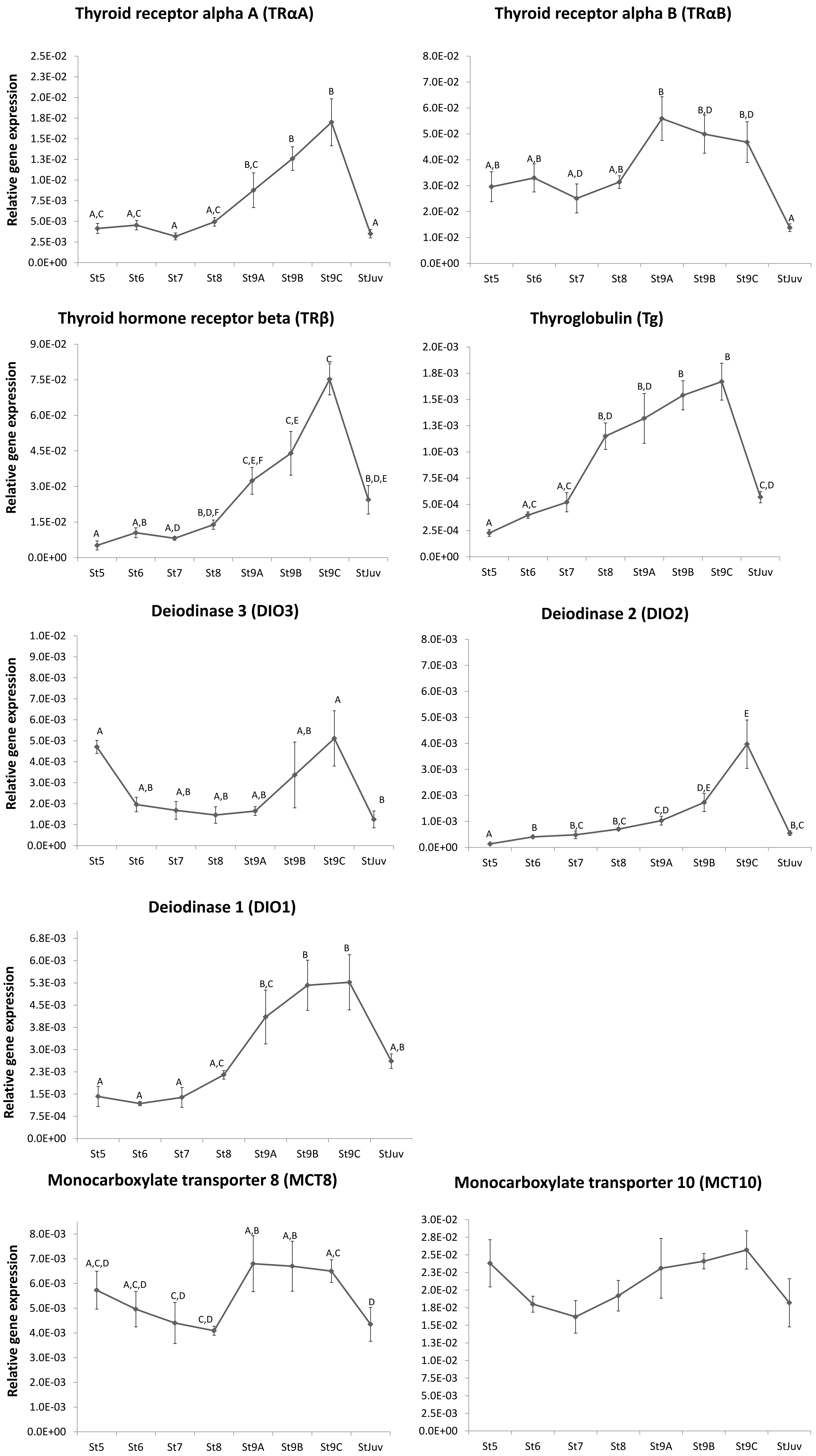
Thyroid receptor alpha A (TRαA)
Thyroid receptor alpha B (TRαB)
Thyroid hormone receptor beta (TRβ)
Thyroglobulin (Tg)
Deiodinase 3 (DIO3)
Deiodinase 2 (DIO2)
Deiodinase 1 (DIO1)
Monocarboxylate transporter 8 (MCT8)
Monocarboxylate transporter 10 (MCT10)
Relative gene expression
St5
St6
St7
St8
St9A
St9B
St9C
StJuv
