# Supplementary material for: The transcriptome of metamorphosing flatfish
Source: BMC Genomics. 2016 May 27;17:413. doi: 10.1186/s12864-016-2699-x (PMC4884423; doi:10.1186/s12864-016-2699-x)
Supplement: Additional file 15: — Quantitative RT-PCR (qPCR) of the relative expression of ribosomal protein L7 (RPL7), 40S ribosomal protein S30 (FAU); alpha-globin 1 (Gloα1), carboxypeptidase A2 (Cpa2), apolipoprotein A-I (ApoAI) and type I keratin isoform 2 (Krt1i2). Analysis of the indicated transcripts was performed in whole Atlantic halibut larvae during development (stage 5 to juvenile; n = 5). The results are presented as mean ± SEM of the normalized expression, using the geometric mean of the reference genes RPS4 and EFIAI. Different letters represent significantly different mean values (p < 0.05; one-way ANOVA). (PDF 188 kb) [file 12864_2016_2699_MOESM15_ESM.pdf]

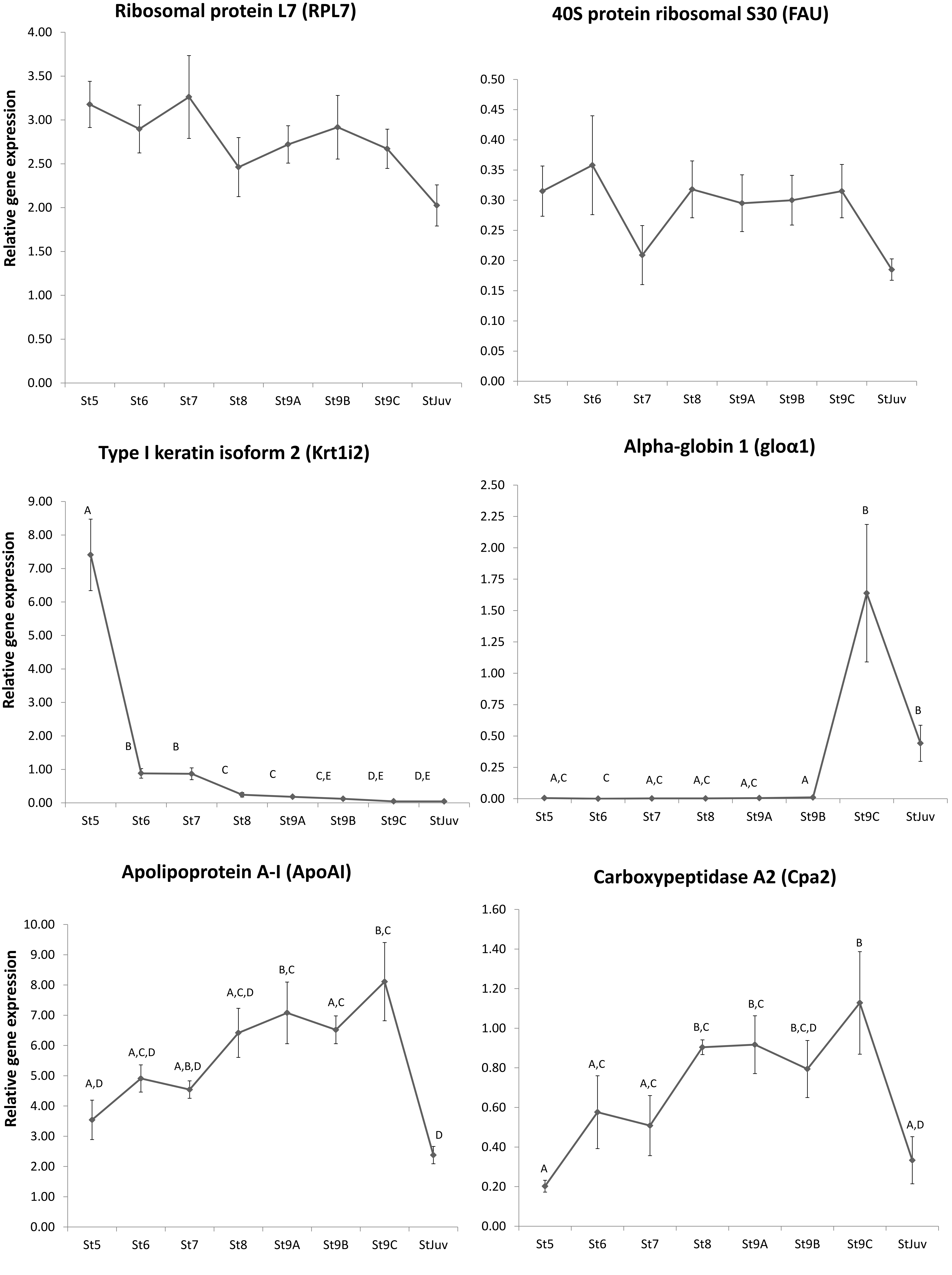

Ribosomal protein L7 (RPL7)
40S protein ribosomal S30 (FAU)
Type I keratin isoform 2 (Krt1i2)
Alpha-globin 1 (gloα1)
Apolipoprotein A-I (ApoAI)
Carboxypeptidase A2 (Cpa2)
Relative gene expression
St5
St6
St7
St8
St9A
St9B
St9C
StJuv
A
B
B
C
C
C,E
D,E
D,E
A,C
C
A,C
A,C
A,C
A
B
B
A,D
A,C,D
A,B,D
A,C,D
B,C
A,C
B,C
D
A
A,C
A,C
B,C
B,C
B,C,D
B
A,D
