# Supplementary material for: The transcriptome of metamorphosing flatfish
Source: BMC Genomics. 2016 May 27;17:413. doi: 10.1186/s12864-016-2699-x (PMC4884423; doi:10.1186/s12864-016-2699-x)
Supplement: Additional file 20: — Specific primers used for qPCR gene expression analysis. Gene symbol, name and function are shown. The annealing temperature (Ta °C), amplicon length (bp), R2 and qPCR efficiency (%) are indicated for each primer pair. (DOCX 17 kb) [file 12864_2016_2699_MOESM20_ESM.docx]

**Additional file 20.** Specific primers used for qPCR gene expression analysis. Gene symbol, name and function are shown. The annealing temperature (TaºC), amplicon length (bp), R^2^ and qPCR efficiency (%) are indicated for each primer pair.

| **Gene symbol** | **Gene name** | **Function** | **Relevance** | **Primer sequence(5’→3’)** | **Annealing temperature (Ta)** | **Amplicon length** | **R^2^** | **PCR efficiency** |
| --- | --- | --- | --- | --- | --- | --- | --- | --- |
| EFIAI | Elongation factor 1 alpha | Protein synthesis | Genes used as reference | F: AAGAGGACCATCGAGAAGTT  R: GTCTCAAACTTCCACAGAGC | 60ºC | 140 | 0.99 | 85% |
| RPS4 | 40S ribosomal protein S4 | Structural component of the small 40S ribosomal subunit |  | F: CAAGTTTGATACTGCCAACCTGTG  R: GGAGAGCCTGGTAGCGAAGC | 60ºC | 172 | 0.99 | 90% |
| RPL7 | Ribosomal protein L7 | Structural component of the small 60S ribosomal subunit | Genes without modification during development | F: TTCTCGGTGGACGCAATGG  R: GCCAGCATCTTCTTTGACACG | 60ºC | 120 | 0.99 | 100% |
| FAU | 40S ribosomal protein S30 | Structural component of the small 40S ribosomal subunit |  | F: TTGAGGTGACCGGACAGGAAAC  R: CACAGGATGCCAGGGAGGAATCA | 60ºC | 135 | 0.99 | 89% |
| Gloα1 | Alpha-globin 1 | Oxygen-transporting protein | Genes with modification during development: putative THs responsive | F: CCCACTGGAAGGACCTGAGC  R: CACAGCATACTCAACTCCACCC | 62ºC | 83 | 0.99 | 100% |
| Cpa2 | Carboxypeptidase A2 | Pancreatic carboxypeptidase activity |  | F: CCTAAGAGTCAATGTGCGGTCG  R: GGAGCGTGGCACTCGGATG | 62ºC | 136 | 0.99 | 92% |
| ApoAI | Apolipoprotein AI | Major protein component of high density lipoprotein (HDL) in plasma |  | F: GCTGACTTCCGTGCCTCTG  R: TATCTGCTGAGTGCTTGGTATG | 62ºC | 193 | 0.99 | 97% |
| Krt1i2 | Type I keratin isofom 2 | Major structural proteins in epithelial cells |  | F: GCTGGAGGCGGCTTCAAC  R: CCTCGGGTTTGGTCTTGCTC | 62ºC | 181 | 0.99 | 100% |
| MCT8 | Monocarboxylate transporter 8 | Transport of iodo-thyronines (T_3_ and T_4_) | Genes involved in THs production, availability and metabolism | F: TTCGGCTGGCTGGTGGTGCTC  R: CGTCTGGGTCTGCGTGCTCCTTC | 60ºC | 115 | 0.99 | 90% |
| MCT10 | Monocarboxylate transporter 10 | Aromatic amino acid transporter. Can transport THs |  | F: GCTCCGAGAACGATGACGAC  R: GTGAAGACGCTGACGATGG | 60ºC | 100 | 0.99 | 94% |
| DIO1 | Deiodinase 1 | Activation and inactivation of THs by deiodination |  | F: CCAAAAACTCCTCGTCTATGTCT  R: CTTGGTGAGGCTTGGTGAAATAA | 60ºC | 108 | 0.99 | 90% |
| DIO2 | Deiodinase 2 | Major role in activation of THs by deiodination |  | F: TGGACGCATACAAGCAGGTG  R: TGGCACATTGGTCACATTACTG | 60ºC | 106 | 0.99 | 99% |
| DIO3 | Deiodinase 3 | Major role in inactivation of THs by deiodination |  | F: ACTCCAGATAGATAGATTGTGTC  R: TAAACAAACCTTTTTTTCCTGAA | 60ºC | 178 | 0.99 | 95% |
| Tg | Thyroglobulin | Production of THs |  | F: CCAGCAACAAGAAGACATCCC  R: CGGACAGTGAGGAGCAGAG | 62ºC | 151 | 0.99 | 95% |
| TRαA | Thyroid receptor alpha A | THs receptor activity |  | F: GAATCGGGAGAAGAGGAAGCG  R: GACCCTGACCGATATCATCCGA | 62ºC | 179 | 0.99 | 100% |
| TRαB | Thyroid receptor alpha B |  |  | F: TCCAGAGCCCACCGGCGCC  R: GGGGAGGCCGATTTTGTCT | 62ºC | 129 | 0.99 | 99% |
| TRβ | Thyroid receptor beta |  |  | F: CAAGCGTCCATGGCAAATACAC  R: CAAAGTCCACCACTCGGGTT | 62ºC | 106 | 0.99 | 100% |
